# Advanced Pharmaceutical Nanotechnologies Applied for Chinese Herbal Medicines

**DOI:** 10.1002/advs.202500167

**Published:** 2025-06-20

**Authors:** Jiameng Li, Ya‐Li Zhang, Tong Jin, Zhaokui Jin, Mengliang Zhu, Guangchao Qing, Jinchao Zhang, Zhisen Wang, Yan Mu, Jin Li, Qian Hua, Xing‐Jie Liang

**Affiliations:** ^1^ State Key Laboratory of Respiratory Disease Guangzhou Institute of Respiratory Health the Key Laboratory of Advanced Interdisciplinary Studies the First Affiliated Hospital of Guangzhou Medical University Guangzhou 510120 China; ^2^ CAS Key Laboratory for Biomedical Effects of Nanomaterials and Nanosafety Chinese Academy of Sciences and National Center for Nanoscience and Technology of China Beijing 100190 China; ^3^ School of Life Sciences Beijing University of Chinese Medicine Beijing 102488 China; ^4^ School of Biomedical Engineering Guangzhou Medical University Guangzhou 511436 China; ^5^ College of Chemistry & Materials Science Key Laboratory of Medicinal Chemistry and Molecular Diagnosis of Ministry of Education Chemical Biology Key Laboratory of Hebei Province Hebei University Baoding 071002 China; ^6^ Shijiazhuang Zangnuo Tibetan Medicine Research Institute Shijiazhuang 051430 China; ^7^ School of Life Sciences Beijing University of Chinese Medicine Liangxiang Higher Education Park Beijing 47839 China; ^8^ School of Nanoscience and Techonlogy University of Chinese Academy of Sciences Beijing 100049 China

**Keywords:** bioactive materials, chinese herbal medicines, material basis, nanocarriers, self‐assembly

## Abstract

Over centuries of clinical practice, Chinese herbal medicines (CHMs) have gained widespread recognition for their efficacy in treating various diseases. However, their complex material basis and relatively mild therapeutic efficacy limit their modernization and quality control. Recently, the application of pharmaceutical nanotechnology to CHMs has not only enhanced their efficacy, but also helped elucidate their material basis, thereby substantially advancing their modernization. Nano‐modified CHMs have shown improvements in multiple aspects, such as bioavailability, targeting ability, toxicity reduction, and sustained release. In this review, nano‐strategies for emerging, revolutionary, and promising pathways for modernizing CHMs are revealed. First, the development, application potential, particularities, and limitations of CHMs are reviewed. Subsequently, a systematic and comprehensive analysis of the two nano‐strategies for optimizing CHMs are presented, highlighting the distinct characteristics of the carrier‐free and carrier‐based approaches. The specific advantages of these strategies, including improved bioavailability, increased targeting ability, reduced toxicity, and controlled release, are discussed. The novel research directions resulting from the application of pharmaceutical nanotechnology to CHMs are also explored, such as elucidating CHM treatment theories, combining traditional Chinese medicine topical therapies, drug screening, and expanding innovative drug formulations. Finally, the challenges and opportunities in this field are addressed to inspire future research.

## Graphical abstract

This review offers a comprehensive overview of pharmaceutical nanotechnology strategies to address limitations associated with the complex material basis and relative mild therapeutic efficacy of Chinese herbal medicines, and summarizes the advantages of these strategies. In addition, it also concludes with recommendations for future challenges and developments in this field.

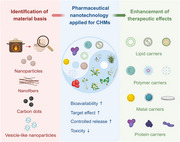

## 1 Introduction

Chinese herbal medicines (CHMs), derived from ancient medical practices with distinct theories and methodologies, show substantial potential for individualized treatment compared with that of modern medicine, due to centuries of clinical testing and refinement.^[^ ^1^ ^]^ Additionally, CHMs have proven to be irreplaceable in modern society.^[^ ^2^ ^]^ Containing abundant natural compounds with diverse scaffolds and functional groups, CHMs can treat diseases through intricate mechanisms of action characterized by multiple components, targets, and pathways. These compounds, also known as natural ingredients from CHMs (NICHMs), are currently one of the primary sources of new drugs, such as artemisinin for malaria^[^ ^3^ ^]^ and polycystic ovarian syndrome,^[^ ^4^ ^]^ artesunate for cardiac fibrosis,^[^ ^5^ ^]^ paclitaxel for tumors,^[^ ^6^ ^]^ and berberine for antimicrobial purposes.^[^ ^7^ ^]^ However, the limitations of the complex material basis and the moderate therapeutic efficacy of CHMs severely restrict their further promotion and development.^[^ ^8^ ^]^ Therefore, CHMs have immense potential for human health^[^ ^9^ ^]^ and addressing these challenges is essential for their modernization.

The integration of pharmaceutical nanotechnology has provided promising strategies to overcome the challenges of CHMs.^[^ ^10^ ^]^ Pharmaceutical nanotechnology^[^ ^11^ ^]^ involves the use of techniques to convert raw pharmaceuticals into nanoparticles (NPs) or formulate nanocarrier delivery systems.^[^ ^12^ ^]^ Common nanocarriers include lipid‐, polymer‐, inorganic‐, and protein‐based carriers. Liposomes were initially described in 1964,^[^ ^13^ ^]^ therefore, nanodrugs have become a prominent area of biomedical research.^[^ ^14^ ^]^ During the COVID‐19 pandemic, the successful application of mRNA vaccines was enabled by advancements in pharmaceutical nanotechnology.^[^ ^15^ ^]^ Further, the 2023 Nobel Prize in Physiology or Medicine garnered interest in the field of nanomedicine by acknowledging innovations in mRNA vaccines. To date, more than 60 nanodrugs have been approved for clinical use worldwide,^[^ ^16^ ^]^ predominantly for cancer therapy, with additional applications in the treatment of hematological diseases, anti‐infective therapies, and neurological disorders.^[^ ^17^ ^]^ Notably, nanostructures exhibit unique spatial dimensions and surface chemical properties, such as high specific surface area, porous structures, and amphiphilicity,^[^ ^18^ ^]^ which make them effective in addressing the challenges associated with traditional small‐molecule or biomacromolecule drugs, such as low bioavailability, poor biocompatibility, susceptibility to degradation, and low targeting efficiency.^[^ ^19^ ^]^ Thus, the application of pharmaceutical nanotechnology to CHMs is a highly promising strategy for overcoming their limitations.^[^ ^20^ ^]^

Researchers have undertaken extensive efforts to optimize CHMs using pharmaceutical nanotechnology as a feasible and highly anticipated strategy. In late 1900s, Chinese scholars first proposed the concept of nano‐modified CHMs. Nano‐modified CHMs are formulated from raw CHM materials, extracts, or NICHMs using pharmaceutical nanotechnology. Recently, researchers have discovered that nanostructures, specifically composite spheres, cubes, and tetragonal bipyramids, are frequently formed during CHM decoctions.^[^ ^21^ ^]^ Moreover, vesicle‐like nanostructures were identified in fresh herbal materials,^[^ ^22^ ^]^ and carbon dots (CDs) derived from carbonized CHMs were discovered. Inspired by naturally self‐assembled herbal nanomaterials (SHNs), researchers have begun to artificially induce the self‐assembly of NICHMs to enhance their therapeutic efficacy. Various types of nanocarriers have been introduced to improve the performance of CHMs, with promising outcomes. Notably, these nanostructures inherit the therapeutic effects of original herbs while providing extra benefits derived from nano‐modification, exhibiting increased bioavailability, enhanced target specificity, enable controlled release, and reduced toxicity compared with those of their original materials. Furthermore, they can improve the therapeutic outcomes of acupuncture or patches, which are hallmark topical therapies in traditional Chinese medicine (TCM),^[^ ^23^ ^]^ by improving the drug delivery systems of medical devices. Nanomaterials can contribute to the scientific elucidation of traditional concepts in CHMs, diversify dosage forms, and facilitate drug screening. Therefore, the application of pharmaceutical nanotechnology to CHMs presents opportunities in healthcare.

**Scheme 1:**
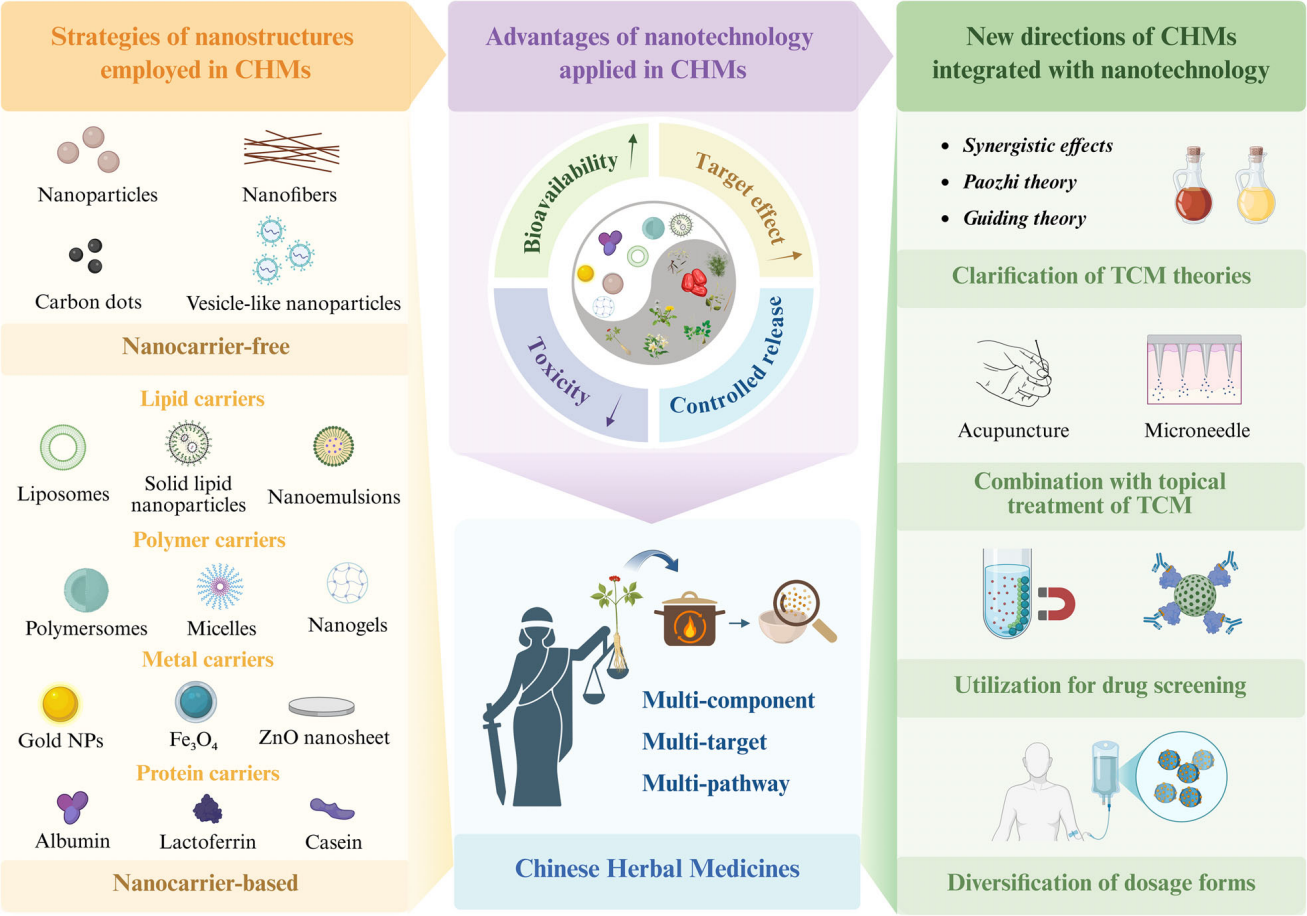
Schematic representations of the strategies, advantages, and new directions of pharmaceutical nanotechnology applied in CHMs.

Overall, pharmaceutical nanotechnology has emerged as a promising strategy to address the limitations associated with CHMs.^[^ ^24^ ^]^ However, to the best of our knowledge, a systematic review clarifying the specific challenges that this technology can address in the CHM field and its potential for future applications is currently lacking. This review provides valuable insights to guide future research. Herein, we examined the contributions of pharmaceutical nanotechnology in overcoming two major challenges in the modernization of CHMs, the complex material basis and relatively mild therapeutic efficacy, through nanocarrier‐free and nanocarrier‐based strategies. Subsequently, the advantages of nanostructures for addressing the limitations of CHMs were explored. We also investigated how this advanced technology has been employed to address other challenges associated with CHMs, including the scientific elucidation of traditional concepts, optimization of topical therapies, diversification of dosage forms, and facilitation of drug screening. Finally, we discuss the remaining challenges in the field of CHM and the potential of pharmaceutical nanotechnology to address these issues (**Scheme** **1**).

## 2 Origins and Potential of CHMs

Rooted in over 2000 years of clinical practice documented in classical texts, such as *the Yellow Emperor's Inner Classic*, a unique medical system was developed for CHMs.^[^ ^25^ ^]^ Unlike the modern medical model, TCM uses CHMs through a process of “syndrome differentiation and individualized treatment,” adhering to the principle of “sovereign‐minister‐assistant‐messenger” in the establishment of personalized prescriptions.^[^ ^26^ ^]^ This concept can be regarded as a precursor to modern ideas of polypharmacy and synergistic drug interactions. With the advancements in science and technology, CHMs are increasingly being scientifically applied.

### 2.1 Development of CHMs

CHMs originated in primitive societies where humans primarily relied on hunting and foraging for natural plants for sustenance. During the search for food, certain plants were discovered to alleviate pain and discomfort. Consequently, many of the early CHM substances were foods that also had medicinal properties, known as “food‐medicine homology.”^[^ ^27^ ^]^ As society advanced, people began to consciously collect, cultivate, and domesticate plants with therapeutic effects and document their findings for future generations. Most CHMs are derived directly from nature, including plants,^[^ ^28^ ^]^ animals,^[^ ^2^, ^29^ ^]^ and minerals.^[^ ^30^ ^]^ In modern times, NICHMs have been increasingly identified, representing an important extension of traditional CHM forms.^[^ ^31^ ^]^

With the advancements in science and technology, CHMs continue to contribute significantly to the global health industry. In 2015, Youyou Tu was awarded the Nobel Prize for her discovery of artemisinin, which validated the scientific value of the ancient *Elbow‐Width Emergency Prescriptions*.^[^ ^32^ ^]^ In 2016 and 2018, arsenic trioxide, derived from traditional “realgar” (arsenic sulfide), was approved by the European Union and the US Food and Drug Administration (FDA) as a first‐line therapy for acute promyelocytic leukemia.^[^ ^33^ ^]^ During the COVID‐19 pandemic in 2020, the World Health Organization (WHO) released a report titled, “WHO Expert Meeting on Evaluation of Traditional Chinese Medicine in the Treatment of COVID‐19,” which highlighted that CHMs can effectively treat COVID‐19, reduce the progression from mild to severe cases, shorten viral clearance time, and improve clinical outcomes for patients with mild and moderate COVID‐19. Recently, in randomized clinical trials, the CHM formula, Tongxinluo, was identified as a potential therapy for alleviating acute myocardial infarction.^[^ ^34^ ^]^ These achievements mark the transformation of CHMs from an Eastern traditional practice to a globally shared health solution through “scientification.”

### 2.2 Particularities of CHMs in Treating Diseases

CHMs exhibit multi‐component, multi‐target, and multi‐pathway features in disease treatment,^[^ ^35^ ^]^ which are distinct from those of modern medicines. The main causes of these differences are as follows.

First, in terms of origin, CHMs, as natural remedies, are environmentally friendly, diverse, and renewable. Moreover, because most CHMs are derived from plants, they contain a wide range of small‐molecule compounds with diverse structural frameworks and multiple pharmacological activities.^[^ ^36^ ^]^ Second, during application, CHMs are characterized by the use of multi‐herb combinations, known as formulas,^[^ ^37^ ^]^ which are regarded as an integrated “whole” for therapeutic purposes.^[^ ^38^ ^]^ Importantly, these combinations are not arbitrary but are formulated according to the TCM principle of “sovereign‐minister‐assistant‐messenger,” with specific proportions of herbs by weight.^[^ ^39^ ^]^ The sovereign herb plays a primary role in treating the disease, the minister herb supports and enhances the efficacy of the sovereign herb, the assistant herb addresses secondary symptoms related to the disease, and the messenger herb harmonizes the interactions among different herbs. Together, these components exert multiple therapeutic effects, providing comprehensive symptom relief in a manner analogous to “team‐based collaboration.”^[^ ^40^ ^]^ Therefore, these differences in origin and usage principles provide a unique CHM biological effect profile that is distinct from that of modern medicines.

Specifically, a single active component of a CHM can target multiple sites, whereas multiple active components can act on the same target.^[^ ^41^ ^]^ The changes in the targets will, in turn, further affect the signaling pathways. These synergistic and additive effects provide a distinctive treatment paradigm compared with those of modern medicine.^[^ ^42^ ^]^ For example, Tongxinluo, an antioxidant CHM formulation composed of 12 herbs, is considered a novel neuroprotective agent with anti‐inflammatory and antioxidant properties.^[^ ^34^ ^]^ Previous neuroprotective agents have specific targeted sites or demonstrated clear benefits for a particular pathway in preclinical studies. However, in cerebral ischemia, several damaging pathways within the ischemic cascade can simultaneously deteriorate and interact with each other. Fortunately, the multi‐pathway neuroprotection of Tongxinluo, which targets several aspects of ischemic injury (with its 12 chemical constituents believed to exert vasodilatory, antiplatelet, anticoagulant, thrombolytic, and lipid‐lowering effects), may offer advantages over strategies targeting a single pathway. In summary, CHMs serve as important complements to modern medicine, and their comprehensive regulatory characteristics of “multi‐component, multi‐target, and multi‐pathway” interactions have broad prospects for treating complex diseases.

### 2.3 Limitations of CHMs

#### 2.3.1 Complex Material Basis

The material basis of CHMs refers to the chemical constituents or groups of constituents within the herbs that are responsible for their therapeutic effects.^[^ ^43^ ^]^ Although the multi‐component synergistic action endows CHMs with significant advantages and unique therapeutic properties, the complex chemical composition also poses challenges in the elucidation of biological mechanisms, quality control, and standardization. The purpose of elucidating the material basis of CHMs is to control the quality of CHMs^[^ ^44^ ^]^ and develop of new drugs.^[^ ^45^ ^]^

Since the early 1900s, researchers have continuously applied the latest analytical techniques of their time to investigate the material basis of CHMs. Initially, phytochemical methods were used to isolate individual compounds from CHM extracts, identify their chemical structures, and subsequently test their biological activities. This classic method has been extensively used. For example, falcarinophthalide A, a highly promising lead compound in *Angelica sinensis*, exhibits in vitro anti‐osteoporotic activity.^[^ ^46^ ^]^ However, owing to their poor water solubility, the bioavailability of most NICHMs is low. Consequently, many pharmacological NICHMs do not achieve their full therapeutic potential when administered as single agents, which limits the broader application of CHMs in the health domain. Subsequently, researchers have hypothesized that the material basis of CHMs may also manifest in the form of total constituents or extracts. For instance, total flavonoids from *Inula japonica* exhibit anti‐inflammatory and antioxidant effects^[^ ^47^ ^]^ whereas extracts from *Salsola collina* combats aging.^[^ ^42^ ^]^ Recently, with the help of nanotechnology, various nanostructures derived from CHMs, such as particles, micelles, vesicles, and nanogels, have been discovered and found to possess distinct therapeutic effects compared with that of their individual components. NPs in CHM decoctions may represent the primary therapeutic forms responsible for their efficacy, such as the NPs identified in the QY305 decoction.^[^ ^48^ ^]^ Moreover, amphiphilic NPs formed by the self‐assembly of berberine and chlorogenic acid exhibited superior anti‐inflammatory effects compared to their respective free compounds.^[^ ^49^ ^]^ Therefore, nanostructures have recently emerged as new and crucial insight into the material basis of CHMs.

#### 2.3.2 Relatively Mild Therapeutic Efficacy

CHMs often exert their therapeutic effects through multi‐target network regulation and systemic fine‐tuning, and are characterized by mild cumulative actions. These characteristics confer several advantages, including the enhanced safety, ability to effectively restore homeostasis over the long term, and avoidance of drug resistance. However, in addition to limitations in bioavailability, the therapeutic effects of CHMs are generally less immediate than those of the single‐target, precise interventions of modern medicines, falling short in the treatment of certain acute and severe conditions. Hence, enhancing the potency of CHMs and expanding their application to acute and severe conditions are urgent challenges that must be addressed.

One effective strategy is the structural modification of NICHMs, which is an important method for innovative drug development in the 21st century. The structural modification of camptothecin has led to the development of anticancer drugs, such as irinotecan and topotecan.^[^ ^50^ ^]^ Additionally, strategies for improving the solubility of NICHMs primarily include the addition of a basic side chain, disruption of aromaticity, interference with hydrogen bonding, and certain subtle structural changes.^[^ ^51^ ^]^ Unfortunately, the complex structures of NICHMs pose challenges for their structural modification and total synthesis. Recently, motivated by nanomedicine, the application of nanocarriers has had an unexpected positive impact on the efficacy of NICHMs. For example, paclitaxel bound to albumin NPs is the preferred drug for ovarian cancer treatment. Besides, inspired by the discovery of nanostructures in CHM decoctions, researchers have increasingly explored the self‐assembly of NICHMs to enhance their therapeutic efficacy. Therefore, nanotechnology has considerable potential for enhancing the efficacy of CHMs.

After outlining the fundamental characteristics of CHMs in disease treatment, the main limitations of their modernization, and the potential of pharmaceutical nanotechnology to optimize CHMs, we will delve into the specific details and provide a comprehensive introduction to the two most representative and extensively studied strategies in the optimization of CHMs using pharmaceutical nanotechnology, nanocarrier‐free and nanocarrier‐based approaches, with an emphasis on their assembly methods and research progress.

## 3 Carrier‐Free Nanostructures Employed in CHMs

Carrier‐free nanostructures are formed at various stages of CHM preparation, including fresh herb extraction, processing, boiling, storage, and even ingestion. In the 1970s, researchers discovered micro‐ to nanoscale particulate matter in CHM preparations from Yunnan Baiyao.^[^ ^52^ ^]^ The discovery of carrier‐free nanostructures served two purposes. It reflects the material basis of the therapeutic efficacy of CHMs. For example, SHNs represent the material basis for CHM decoctions, processed materials, finished products, and in vivo bioactive substances. CDs can be considered the material basis of carbonized CHMs, whereas vesicle‐like NPs can be considered the material basis of fresh CHMs. Additionally, these findings can guide the concentrated extraction and preparation of the therapeutic substances in CHMs, thereby contributing to the enhancement of their efficacy.

The following section introduces the application of carrier‐free nanostructures in CHMs and emphasizes the unique assembly strategies for three categories of nanomaterials: SHNs, CDs, and vesicle‐like NPs. This discussion provides a clear perspective and reference for future research on different types of carrier‐free nanomaterials derived from CHMs.

### 3.1 Self‐Assembled Herbal Nanomaterials

**Figure 1:**
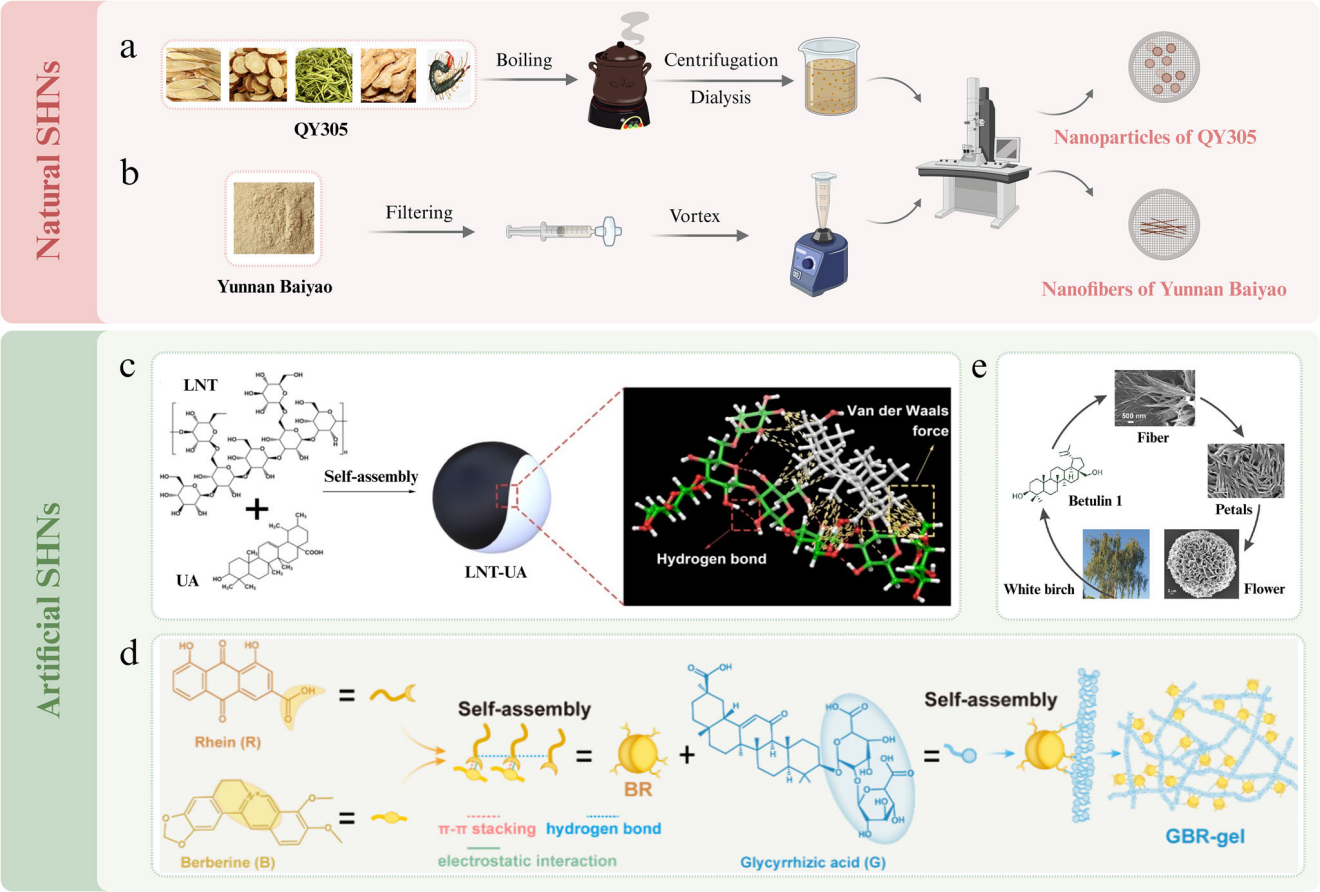
Self‐assembled herbal nanomaterials. Natural SHNs. a) Formula QY305 forms nanoparticles of uniform size through boiling, centrifugation, and dialysis processes.^[^ ^48^ ^]^ b) Yunan Baiyao is dissolved in water, filtered by filter membrane, and vortexed, resulting in a solution containing nanofibers.^[^ ^54^ ^]^ Artificial self‐assembled herbal nanomaterials. c) Ursolic acid (UA) co‐self‐assembles with lentinan (LNT) via van der Waals forces and hydrogen bonding. Reproduced with permission.^[^ ^55^ ^]^ Copyright 2022, Ivyspring. d) GBR‐gel consists of three components: Rhein and berberine (BR) self‐assemble through hydrogen bonding, π–π stacking, and electrostatic interactions. Fibrous structures self‐assembled by glycyrrhizic acid is bonded to the surface by BR particles and creating GBR‐gel. Reproduced with permission.^[^ ^56^ ^]^ Copyright 2024, ACS. e) Betulin spontaneously self‐assembles in various media, developing flower‐like architectures ranging from nanoscale to microscale through fibrillar network formation. These structures can entrap fluorophores. Reproduced with permission.^[^ ^57^ ^]^ Copyright 2015, ACS.

SHNs are organized structures formed through the spontaneous arrangement of components via non‐covalent interactions, including hydrogen bonding, electrostatic forces, van der Waals forces, π–π interactions, hydrophobic interactions, and coordination interactions (**Figure** **1**).^[^ ^53^ ^]^ Theses nanomaterials can be classified into two types: the natural and the artificial. Natural SHNs result from molecular recognition and self‐assembly of components during preparation or in vivo processes. In contrast, artificial SHNs, inspired by their natural counterparts, are synthesized by controlling reaction conditions, such as temperature, pH, and salt ion concentration, to induce the self‐assemble actions of known NICHMs into nanostructures, thereby enhancing therapeutic efficacy.

#### 3.1.1 Natural SHNs

In clinical practice, CHM prescriptions are typically prepared by boiling, with historical records tracing the origins of the Yin and Shang dynasties in *Tangye Jingfa* attributed to Yi Yin.^[^ ^58^ ^]^ The boiling process assists in the extraction and transformation of compounds through physical and chemical mechanisms, creating optimal conditions for the formation of SHNs.^[^ ^59^ ^]^ Zhuang et al.^[^ ^60^ ^]^ identified SHNs in 60 herbal and 24 formula decoctions. The natural SHNs in the CHM decoctions were predominantly spherical. Notably, under specific conditions, a broader spectrum of morphologies was observed. Xiang et al.^[^ ^21^ ^]^ examined nearly 40 types of herbal decoctions and demonstrated that these decoctions could generate a variety of inorganic–organic assembled hierarchical SHNs through simple freeze‐thaw processes or the addition of appropriate ions. In addition to the spherical SHNs, other morphologies, such as cubic, tetrahedral, fibrous, and disc‐like structures were observed. Furthermore, these SHNs are not restricted to plant‐based CHM decoctions; animal‐based CHM decoctions can also produce unique assembled products. For instance, the decoction of *Periostracum cicadae* formed distinctive SHNs following freeze‐thaw processes. These findings indicated that SHNs are ubiquitous in CHM decoctions.

SHNs are fundamental pharmacological components of CHM decoctions. Turkish galls,^[^ ^61^ ^]^ composed of 99.15% gallic catechols, along with minor amounts of resin and protein, have been used for ulcerative colitis (UC) treatment since the seventh century, as documented in *Tang Materia Medica*. Gallic decoctions contain numerous SHNs with regular morphologies, demonstrating strong antibacterial properties, potent antioxidant activities, and potential applications in pH‐responsive antibacterial therapy.^[^ ^62^ ^]^ Similarly, SHNs in the QY305 decoction, with a size of 240.2 ± 6.4 nm, alleviated cutaneous adverse reactions and diarrhea caused by epidermal growth factor receptor inhibitors (Figure 1a).^[^ ^48^ ^]^ SHNs found in a Qingxuechushi mixture (a formula primarily used to treat acute dermatitis, rashes, and psoriasis), with sizes ranging from 200 to 600 nm, contain compounds, such as baicalin, paeoniflorin, and liquiritin, which exhibit therapeutic effects in a psoriasis mouse model.^[^ ^63^ ^]^ Moreover, SHNs derived from *Astragalus membranaceus* and *A. sinensis* in the Danggui Buxue decoction may constitute the chemical basis for the molecular mechanisms underlying the treatment of isoproterenol‐induced myocardial fibrosis.^[^ ^64^ ^]^ Collectively, these findings indicate that SHNs are the most active components of the original decoctions.

In addition to the boiling step, SHNs can also form during other preparation stages of CHMs. According to TCM principles, raw herbs must undergo *paozhi* (specific preparation methods) to become suitable for clinical use.^[^ ^65^ ^]^ Common *paozhi* techniques, such as processing with heat and adjuvants,^[^ ^66^ ^]^ can induce changes in the chemical composition or structure of components, resulting in the formation of SHNs.^[^ ^59^ ^]^ For instance, a novel polysaccharide, VBCP2.5, isolated from vinegar‐baked *Radix Bupleuri*, was found to form micelles at a concentration of 52.574 µg mL^−1^, exhibiting immuno‐enhancing effects on macrophages.^[^ ^67^ ^]^ SHNs can also form during the storage of CHM formulations because of the aggregation of lipophilic components (e.g., alkaloids, aglycones, and volatile oils), surfactants (e.g., saponins, phospholipids, and sterols), and high‐molecular‐weight substances (e.g., starch, proteins, and tannins). Lenaghan et al.^[^ ^54^ ^]^ observed uniform bioactive nanofibers in Yunnan Baiyao (Figure 1b). These fibers, ranging from 86 to 726 nm in length and 20 to 29 nm in diameter with an average height of 3.93 nm, were found in bundles that often overlapped or were in close proximity. In addition, SHNs are formed during the in vivo metabolism of CHMs. Gardenia blue SHNs were found in rat feces following the administration of geniposide, which was metabolized into methylamine and genipin by β‐glucosidase in vivo.^[^ ^68^ ^]^ The pigments formed supramolecular assemblies with spherical nanostructures averaging 3.3 nm in diameter. During assembly, genimethylamine polymerized into dimers, which then self‐assembled through π–π stacking, hydrophobic, and electrostatic interactions. In a word, based on the aforementioned structural and pharmacological features, nanoscale structures have been observed in various processes of CHM products and are the existing critical material basis for their pharmacological efficacy.

#### 3.1.2 Artificial SHNs

Inspired by natural SHNs, current research has increasingly focused on artificial SHNs with well‐defined components, controllable sizes, and enhanced therapeutic efficacy (Table 1). Artificial SHNs can be categorized as follows:

**Table 1:** Self‐assembled herbal nanomaterials derived from NICHMs with diverse morphologies for treating different diseases.

NICHMs	Structures of NICHMs	Nanostructures	Co‐assembled NICHMs	Structures of co‐assembled NICHMs	Diseases treated	Refs.
Ursolic acid	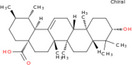	Spherical nanoparticles	Lentinan	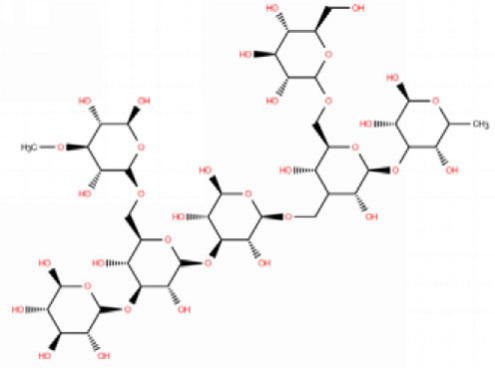	Colorectal cancer	[55]
Rhein	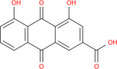	Hydrogel	Glycyrrhizic acid, berberine	–	Traumatic brain injury	[56]
		Hydrogel	–	–	Neural inflammation	[70]
Berberine	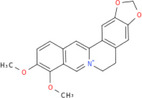	Spherical nanoparticles	Magnolol	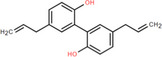	Ulcerative colitis	[69]
		Spherical nanoparticles	Baicalin	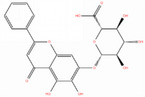	Antibacteria	[72]
		Nanofibers	Wogonoside	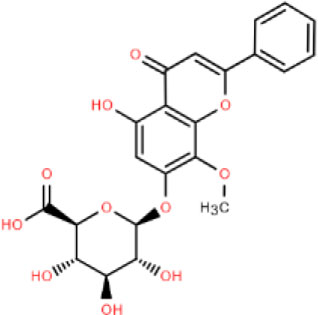	Antibacteria	[72]
		Spherical nanoparticles	Tannic acid	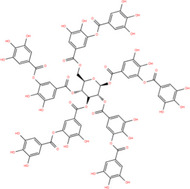	Ulcerative colitis	[73]
		Spherical nanoparticles	Chlorogenic acid	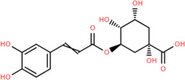	Ulcerative colitis	[74]
		Spherical nanoparticles	Hesperetin	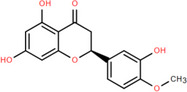	Ulcerative colitis	[75]
		Linear nanoparticles	Aristolochic acid	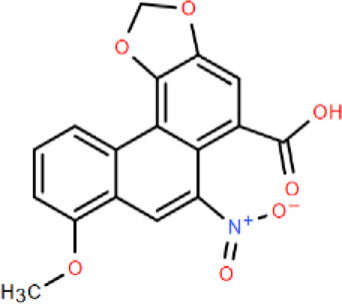	Reduction of acute nephrotoxicity	[76]
Glycyrrhizic acid	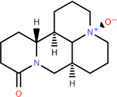	Micelles	Oxymatrine	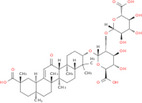	Anti‐photoaging	[71]
		Micelles	Tanshinone IIA	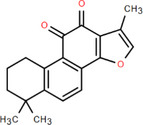	Glioblastoma	[77]
		Micelles	Baicalin	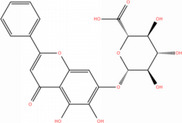	–	[78]
		Micelles	Norcantharidin	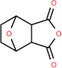	Anticancer	[79]
Aconitine	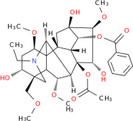	Spherical nanoparticles	Licorice protein	–	Reduction of toxicity	[80]
Celastrol	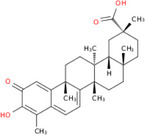	Spherical nanoparticles	Erianin	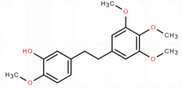	Breast cancer	[81]
Oleanolic acid	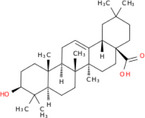	Micelles	–	–	Anticancer	[82]
Curcumin	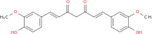	Spherical nanoparticles	Tannic acid	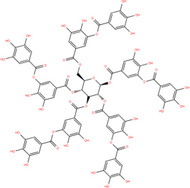	Radioprotection	[83]

*Spherical NPs*: Berberine and magnolol can form spherical NPs through self‐assembly in aqueous solutions via electrostatic attraction and π–π stacking, exhibiting anti‐inflammatory effects for treating UC.^[^ ^69^ ^]^ Lentinan and ursolic acid self‐assembled into SHNs to treat colorectal cancer via hydrogen bonding and van der Waals forces (Figure 1c).^[^ ^55^ ^]^

*Hydrogel*: Rhein underwent self‐assembly to form a uniform orange–red hydrogel for the treatment of neural inflammation.^[^ ^70^ ^]^ The resulting scaffold comprised a 3D network of nanofibers with an average diameter of ≈30 nm and lengths exceeding several micrometers. Similarly, Rhein, berberine, and glycyrrhizic acid were used to form a self‐assembled hydrogel for the treatment of traumatic brain injury (Figure 1d).^[^ ^56^ ^]^ Initially, particles were formed via π−π stacking between the quinoline ring of berberine and the anthraquinone ring of Rhein, with electrostatic interactions stabilizing the structure. Subsequently, berberine‐Rhein NPs, held by hydrogen bonds, self‐assembled within a fibrous structure formed by glycyrrhizic acid. Ultimately, the self‐assembly process resulted in a multicomponent structure, with berberine‐Rhein NPs adhering to the surface via cross‐linking.

*Micelles*: The self‐assembly of glycyrrhizic acid and oxymatrine into micelles enhances the skin permeability of the signaling peptides and their anti‐photoaging efficacy.^[^ ^71^ ^]^ In particular, micelles with a glycyrrhizic acid‐to‐oxymatrine molar ratio of 1:3 exhibited the highest viscosity and intermolecular interactions.

*Others*: Betulin, extracted from the bark of white birch, can spontaneously self‐assemble in different media, forming flower‐like nanostructures through the creation of fibrillar networks (Figure 1e).^[^ ^57^ ^]^ NICHMs can self‐assemble into nanostructures and exhibit excellent therapeutic potential.

The self‐assembly conditions can influence the formation of artificial SHNs, further affecting their pharmacological activity. The morphology of SHNs is crucial to their efficacy. Berberine and flavonoid glycosides can self‐assemble into spherical nanostructures and nanofibers, driven primarily by electrostatic and hydrophobic interactions.^[^ ^72^ ^]^ Spherical nanostructures exhibited enhanced antibacterial activity, whereas nanofibers exhibited weaker effects compared with the free berberine. These differences result from the distinct spatial configurations and self‐assembly mechanisms. Additionally, pH influences the self‐assembly process. The optimal pH range for Rhein gel formation was 8.0 to 9.4. When the pH exceeded 9.4, the hydrogel collapsed and formed a blood‐red solution with short, ribbon‐like structures. Within the pH range of 8.0 to 6.8, the sample remained a viscous gel containing long fibers and several short fibers distributed on the surface, rather than a translucent gel. When the pH dropped below 6.8, precipitate formation occurred, accompanied by the formation of short, rod‐like structures. These findings indicate that nanogel formation is closely related to the degree of carboxyl deprotonation of the Rhein molecule which is influenced by pH. Thus, artificial SHNs enhance the therapeutic efficacy of free NICHMs and exhibit properties that are not observed with the free ones, suggesting a considerable potential for further development.

### 3.2 CHMs‐Derived CDs

**Figure 2:**
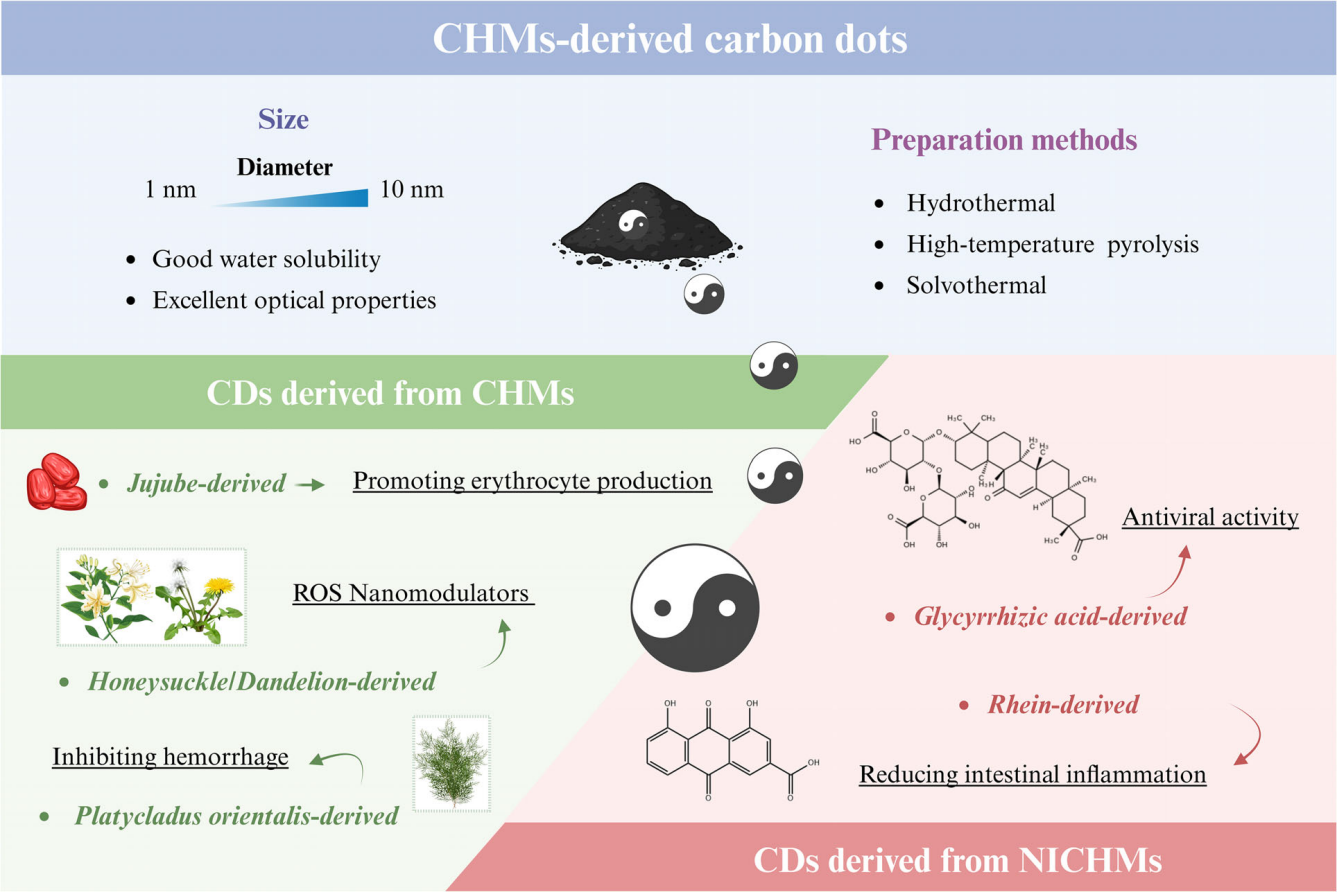
CHMs‐derived carbon dots. It ranges in size from 1 to 10 nm, exhibiting good water solubility and excellent optical properties. For CHMs‐derived carbon dots, common preparation methods include hydrothermal, high‐temperature pyrolysis, solvothermal, and microwave‐assisted techniques. These carbon dots can be sourced from CHMs and NICHMs, each demonstrating distinct pharmacological activities.

Carbonized CHMs have been used for over 2000 years and play a unique role in TCM clinical practice.^[^ ^84^ ^]^ They are widely used to treat hemorrhagic conditions and exhibit antidiarrheal and antiulcer properties.^[^ ^85^ ^]^ Recently, carbonized CHMs with diameters smaller than 10 nm, referred to as CHM‐derived CDs, have been successfully prepared (**Figure** **2**).^[^ ^86^ ^]^ This advancement provides a novel material foundation and perspective for carbonized CHMs.

The synthesis of CHM‐CDs primarily uses two methods: the top‐down approach, which entails the disassembly of CHMs through physical or chemical processes, and the bottom‐up approach, which involves the polymerization or carbonization of NICHMs. These CDs retained the intrinsic pharmacological activity of their parent CHMs and exhibited unique optical properties.

#### 3.2.1 CDs Derived from CHMs

Uniformly spherical CHM‐derived CDs, which possess abundant biological activities, serve as meaningful markers for distinguishing them from other CDs.

*Enhancement of hematopoietic function*: Jujube‐derived CDs were synthesized using the hydrothermal method, with an average size of 2.48 nm, stimulated the self‐renewal of erythroid progenitor cells, which aided in anemia treatment.^[^ ^87^ ^]^ CDs synthesized from *Platyclad cacumen* via a one‐step hydrothermal extraction method accelerated hemostasis by activating platelets and stimulating the coagulation pathway.^[^ ^88^ ^]^ In addition to plant‐based CHMs, animal‐derived CHMs can also be used to fabricate CDs. For example, donkey‐hide gelatin‐derived CDs can activate erythropoiesis and eliminate oxidative stress via a hydrothermal step in the treatment of aplastic anemia.^[^ ^89^ ^]^

*Anti‐inflammatory activity*: CDs from safflower and *Angelica*, characterized by their abundant hydrophilic functional groups, were used as water‐based lubricant additives.^[^ ^90^ ^]^ The hydrophilic groups enhanced the lubricant stability, whereas the spherical carbon cores functioned as nanoball bearings, reducing surface friction and providing superior lubrication properties. These features make them effective in reducing inflammation within the joint capsules and enhancing lubrication between the joint surfaces. The CD‐based ROS nano‐modulators were synthesized using honeysuckle, taxus leaves, and dandelion via a solvothermal method, yielding average diameters of 5.2 ± 0.5, 2.7 ± 0.5, and 7.6 ± 0.5 nm, respectively.^[^ ^91^ ^]^ Phenolic hydroxyl‐containing CDs derived from honeysuckle and dandelion exhibited appropriate redox potentials, enabling them to scavenge cytotoxic ROS while remaining inert toward essential ROS. This property allows for the efficient treatment of chronic inflammation without disrupting vital ROS signaling pathways. The surface C─N/C═N bonds in CDs derived from taxus leaves and dandelion conferred suitable band structures, enhancing absorption in the red region and promoting the efficient generation of O_2_^·−^ upon light irradiation for sterilization. Zhang et al.^[^ ^92^ ^]^ synthesized honeysuckle‐derived CDs using both hydrothermal and carbonization methods, and observed marked differences in their surface functional groups of the resulting CDs. X‐ray photoelectron spectroscopy revealed that the primary distinctions were in nitrogen‐containing groups, with the presence of amino groups being essential for the superoxide dismutase‐like activity and the anti‐inflammatory effects of honeysuckle‐derived CDs.

*Other activities*: CDs synthesized from the CHM formula Xuefu Zhuyu Decoction, specifically *Persicae semen* and *Carthami flos*, were prepared using a green hydrothermal method without organic solvents.^[^ ^93^ ^]^ The resulting CDs were spherical NPs with good dispersibility and mostly had a size range of 2–5 nm, which demonstrated blood‐brain barrier (BBB) permeability and neuronal protection in a mouse model. CDs derived from *Typhae pollen* were synthesized using a simple one‐step pyrolysis method, which alleviated acute kidney injury.^[^ ^94^ ^]^ Importantly, the pharmacological activities of CDs are significantly influenced by their preparation methods. Therefore, CHM‐derived CDs exhibit diverse therapeutic effects, providing a scientific basis for the application of carbonized CHMs.

Importantly, in addition to their pharmacological activities, CHM‐derived CDs exhibit outstanding bioimaging performance. As early as 2012, Zhou et al.^[^ ^95^ ^]^ synthesized water‐soluble fluorescent CDs from watermelon rind, a CHM known for its ability to improve fasting blood glucose levels and liver metabolism.^[^ ^96^ ^]^ This study marks a pioneering step toward the use of CHM‐derived CDs as high‐performance optical imaging probes. The ginger‐derived CDs, with an average particle size of 2.3 nm, exhibited biocompatibility and emitted strong blue fluorescence.^[^ ^97^ ^]^ These CDs have been efficiently applied in both in vitro biological imaging and in vivo experiments, showing anti‐inflammatory activity and promoting wound healing effect. Similarly, CDs derived from the natural herb, *Gynostemma*, are suitable for biological imaging in zebrafish owing to their excellent fluorescence stability and biocompatibility.^[^ ^98^ ^]^ These CDs also displayed antioxidant stress properties in both in vitro and in vivo studies by promoting the mRNA expression of zebrafish‐related genes that encode antioxidant proteins to enhance oxidative stress resistance. In summary, CDs derived from CHMs not only retained pharmacological activities similar to those of carbonized CHMs, but also achieved superior bioimaging capabilities. This dual functionality enables the integration of CHMs into a unified device system.

#### 3.2.2 CDs Derived from NICHMs

The synthesis of CDs can enhance the therapeutic efficacy of the original NICHMs or introduce novel effects to them. For example, glycyrrhizic acid‐derived CDs synthesized via a heating method showed enhanced antiviral activity against influenza A virus through multisite inhibition mechanisms.^[^ ^99^ ^]^ The absence of a discernible crystal lattice in these CDs indicates an amorphous polymeric nature. Similarly, another study demonstrated that glycyrrhizic acid‐derived CDs existed high biocompatibility and effectively prohibited the replication of porcine reproductive and respiratory syndrome viruses.^[^ ^100^ ^]^ Ginsenoside‐derived CDs were synthesized using a one‐step hydrothermal method with reaction times ranging from 1 to 10 h for the treatment of neuroblastoma.^[^ ^101^ ^]^ The CDs prepared for 3 h exhibited minimal lattice structures, likely owing to the short reaction time and incomplete nanostructure formation. After 5 h, CDs displayed a defined lattice structure with a lattice spacing of 0.219 nm. Prolonging the reaction time to 6 and 10 h resulted in more pronounced lattice structures, with lattice spacings of 0.218 and 0.207 nm, respectively. These findings suggest that longer reaction times facilitate more regular self‐assembly of ginsenoside molecules, leading to well‐defined and complete CD structures. Similar to that derived from CHMs, CDs synthesized from NICHMs can also be used for biological imaging. For example, Rhein‐derived CDs not only own enhanced solubility to improve the therapeutic efficacy of UC, but also emit red/NIR‐I light, making them suitable for biological imaging applications.^[^ ^102^ ^]^ In summary, the formation of CDs both augments the therapeutic efficacy of their parent CHMs or NICHMs and introduces optical properties that are not present in traditional ones. These features demonstrate the successful integration of traditional medicine with modern technological advancements.

### 3.3 Vesicle‐Like Nanoparticles from CHMs

The 2013 Nobel Prize in Physiology or Medicine was awarded for the contribution to the field of vesicle transport systems and has subsequently drawn considerable attention to plant‐derived vesicles. Vesicle‐like NPs derived from CHMs are natural structures formed through the self‐assembly of primary and secondary metabolites in plants.^[^ ^103^ ^]^ These vesicles have a membrane structure with a lipid bilayer that serves as the core framework for encapsulating proteins, nucleic acids, and other biologically active substances. They primarily facilitate intercellular communication and enable the efficient and specific transfer of materials and information.^[^ ^86^ ^]^ These properties enable them to cross various biological barriers, leading to their increasing recognition in intestinal flora regulation, anti‐inflammatory therapies, immunomodulation, and anti‐infective treatments.^[^ ^104^ ^]^ Moreover, these nanovesicles represent an important material basis in fresh CHMs.

#### 3.3.1 Regulating Intestinal Flora Balance

**Figure 3:**
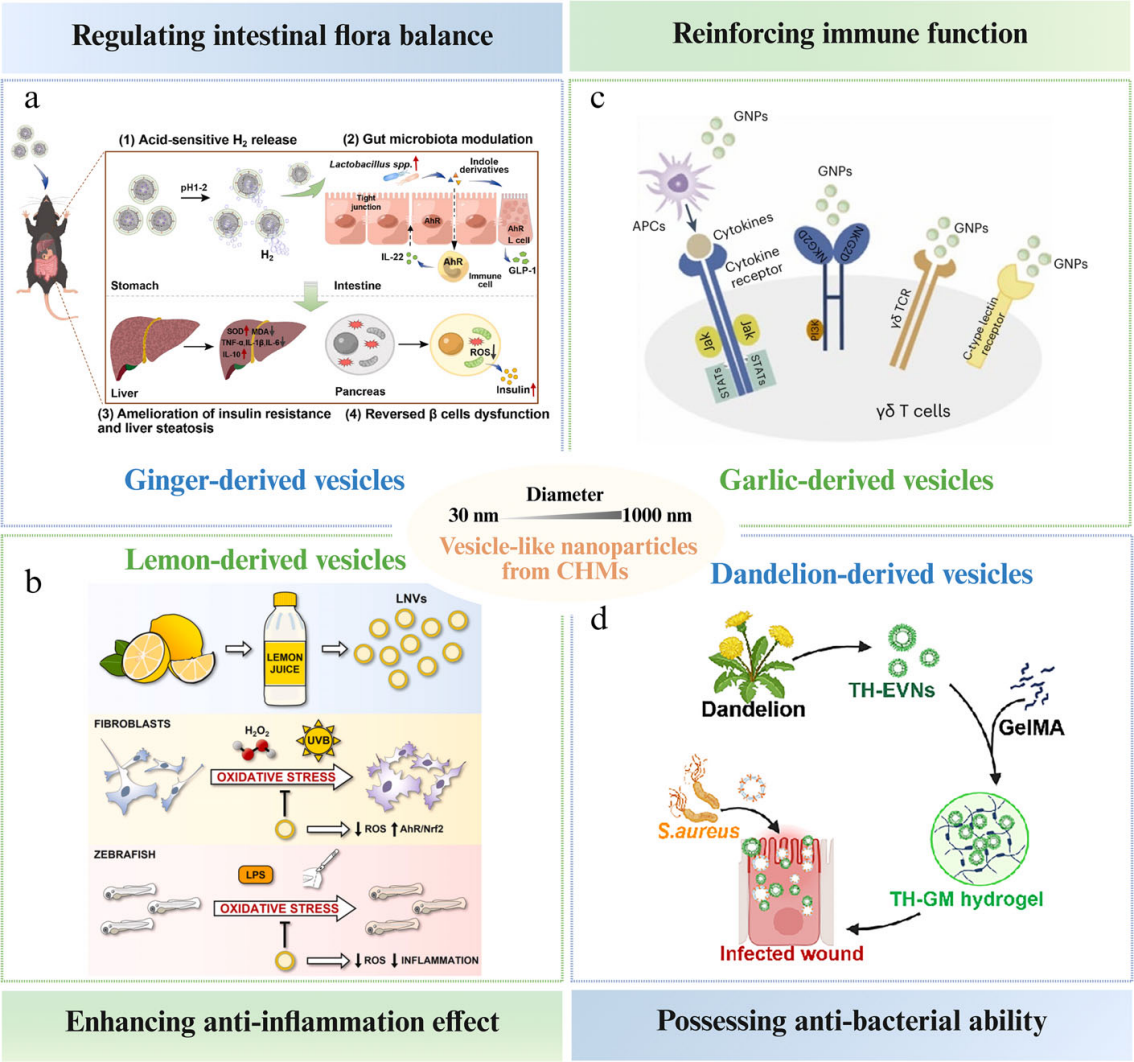
Vesicle‐like nanoparticles from CHMs. a) Regulating intestinal flora balance. For improving insulin resistance and pancreatic β‐cell dysfunction, ginger exosomes with integrating gut‐microbiota remodeling, and hollow mesoporous silica nanoparticles encapsulating ammonia borane with antioxidant therapies, are combined to form a biomimetic, acid‐responsive nano‐hydrogen producer (HMS/A@GE). It could ameliorate insulin resistance, reduce liver steatosis, and reverse β‐cells dysfunction. Reproduced with permission.^[^ ^109^ ^]^ Copyright 2025, Elsevier. b) Enhancing anti‐inflammation effect. Lemon juice‐derived nanovesicles are isolated and characterized, showing high antioxidant and anti‐inflammatory effects in human dermal fibroblasts and zebrafish embryos. Reproduced with permission.^[^ ^115^ ^]^ Copyright 2023, Elsevier. c) Reinforcing immune function. Garlic‐derived nanoparticles (GNPs) are used to activate and expand endogenous γδ T cells. GNPs activate γδ T cells via direct pathways (e.g., C‐type lectin receptor signaling) or indirect pathways (such as cytokines). Reproduced with permission.^[^ ^116^ ^]^ Copyright 2024, Nature. d) Possessing anti‐bacterial ability. Dandelion‐derived extracellular vesicle‐like nanoparticles exhibit anti‐bacterial ability by binding specifically to *S. aureus* exotoxins. These nanoparticles, loaded into a gelatin methacryloyl hydrogel, form a TH‐EVNs‐loaded dressing, offering a potential therapy for *S. aureus* exotoxin‐associated trauma. Reproduced with permission.^[^ ^120^ ^]^ Copyright 2024, Elsevier.

Ginger is cultivated and widely used worldwide^[^ ^105^ ^]^ particularly in Southeast Asia and tropical regions, where it is valued not only as a spice but for its therapeutic properties as well.^[^ ^106^ ^]^ Ginger‐derived vesicles containing microRNAs that target diverse genes in *Lactobacillus rhamnosus* were selectively taken up by *Lactobacillaceae* in a lipid‐dependent manner.^[^ ^107^ ^]^ These microRNAs have the potential to modulate gut microbiota, enhance intestinal barrier function, and alleviate colitis in mouse models. Furthermore, preliminary human studies conducted in Jiangsu, China, revealed increased levels of *Lactobacillus* in the feces of 28 of 58 volunteers who consumed ginger‐derived vesicles. Other researchers have developed ginger‐derived vesicles coated with ZIF‐8 NPs for siRNA therapy targeting UC.^[^ ^108^ ^]^ These vesicles, with a size of 80.36 nm and surface potential of −15.92 mV, remained intact in acidic environments for at least 12 h, providing sufficient time to traverse the stomach and reach colon tissues. Their acid resistance was attributed to a protective barrier formed by a combination of lipids, proteins, glycoproteins, and polysaccharides on the vesicle membrane surface, as well as acid‐base buffering substances within the vesicles that regulate internal pH levels and maintain a stable environment. Building on the advantages of ginger‐derived vesicles, a biomimetic oral hydrogen nanogenerator was developed to manage type 2 diabetes mellitus to regulate intestinal flora and enhance biocompatibility (**Figure** **3**a).^[^ ^109^ ^]^ Garlic‐derived vesicles train *Akkermansia muciniphila* in the gut to produce healthy outer membrane vesicles.^[^ ^110^ ^]^ These vesicles influence brain microglial cells through the gut‐brain axis, thereby reducing brain inflammation caused by high‐fat diets. In addition, pueraria‐derived vesicles alleviated osteoporosis by enhancing autophagy through the gut microbiota‐mediated reduction of trimethylamine‐N‐oxide.^[^ ^111^ ^]^ Fourier transform infrared spectroscopy revealed that the characteristic peaks of the pueraria‐derived vesicles differed from those of the puerarin standard, revealing substantial differences between the two substances.

#### 3.3.2 Anti‐Inflammation

Turmeric‐derived vesicles alleviate colitis symptoms by reducing inflammatory cytokine expression in M1 cells and promoting M1‐to‐M2 cell transition.^[^ ^112^ ^]^ Unlike dried turmeric, which contains high levels of curcuminoids, such as curcumin, demethoxycurcumin, and bisdemethoxycurcumin, turmeric‐derived vesicles are primarily composed of fatty acids, diacylglycerols, triacylglycerols, phosphatidylcholine, and phosphatidylethanolamine. These lipids play a crucial role in the formation of nanovesicles and maintenance of spherical structures. Lemons, a well‐known medicinal food, are a source of nanovesicles with anti‐inflammatory effect.^[^ ^113^ ^]^ Thirty compounds, including flavonoids and organic acids, such as hesperidin, eriocitrin, quercetin, and rutin, were identified in lemon‐derived vesicles using LC‐UV‐MS/MS.^[^ ^114^ ^]^ Another study showed that lemon‐derived vesicles, primarily consisting of vesicles ≈80 nm in diameter and 30 nm in height, exhibited anti‐inflammatory effects by activating the Aryl hydrocarbon receptor/nuclear factor E2‐related factor 2 (AhR/Nrf2) signaling pathway (Figure 3c).^[^ ^115^ ^]^

#### 3.3.3 Reinforcing Immune Function

Vesicles derived from garlic, a common medicinal food, enhance the efficacy of immune checkpoint blockade therapy for solid tumors by inducing γδ‐T cells to produce interferon‐γ in the gut (Figure 3b).^[^ ^116^ ^]^ The vesicles had an average size of ≈120 nm and exhibited a uniform spherical morphology, with garlic‐specific proteins constituting 26.5% of the total protein. Ginseng‐derived vesicles effectively reprogram colorectal tumor‐associated macrophages, stimulate T cell infiltration into the tumor microenvironment, and reduce immune checkpoint expression, thereby enhancing anti‐tumor immune responses.^[^ ^117^ ^]^ Earlier studies have shown that vesicles derived from fresh ginseng inhibit melanoma growth and improve the efficacy of immune checkpoint inhibitors by reprogramming macrophages and altering cold tumor environments.^[^ ^118^ ^]^

#### 3.3.4 Others

Other pharmacological effects of vesicle‐like NPs derived from CHMs include anti‐infective, anti‐tumor, and neurogenesis‐promoting effects. A biomimetic nanoplatform integrating ginger‐derived vesicles with electrodynamic Pd‐Pt nanosheets is proposed.^[^ ^119^ ^]^ The extracted vesicles from ginger exhibited a distinct saucer‐like shape, which is characteristic of typical extracellular vesicles. The incorporation of ginger‐derived vesicles enhanced the ability of the nanoplatform to prolong blood circulation, avoid immune clearance, and accumulate at infection sites, while enabling the nanoplatform to enter bacterial cells in a vesicle lipid‐dependent manner. Vesicles derived from *Dandelion* protected host cells from *Staphylococcus aureus* exotoxin infection in mice (Figure 3d).^[^ ^120^ ^]^ The majority of dandelion‐derived vesicles were ≈187 nm in diameter and contained a total of 112 proteins and 353 lipid species identified through multiple omics analyses. The vesicles derived from *Brucea javanica* fruits were isolated and characterized by their cup‐shaped morphology and uniform distribution without aggregation.^[^ ^121^ ^]^ These vesicles demonstrated efficacy as a nanoplatform for delivering functional miRNAs from *B. javanica*, inducing molecular interference in 4T1 breast cancer cells, which triggered apoptosis through the ROS‐mediated PI3K/Akt/mTOR pathway, highlighting their potential for cancer therapy. Three sizes of ginseng‐derived vesicles were identified: 241.1 ± 3.8 nm, 144.1 ± 2.8 nm, and 340.1 ± 15.9 nm.^[^ ^122^ ^]^ All vesicles exhibited a typical cup‐shaped morphology, similar biocompatibility, and consistent miRNA profiles. These vesicles show marked efficiency in promoting the neural differentiation of bone marrow‐derived mesenchymal stem cells by effectively transferring their incorporated miRNAs, underscoring their substantial potential in neural regenerative medicine.

As a result, vesicle‐like NPs represent novel bioactive materials identified in fresh CHMs. They are distinct from the NICHMs that have garnered considerable attention in the past, differing both structurally and compositionally. Characterized by their multi‐component and multi‐functional nature, these nanovesicles are readily absorbed by living organisms, and can function as biotherapeutic agents and drug delivery vehicles. Such properties may help address the issue of poor bioavailability associated with many active ingredients. Additionally, given their capacity to interact with proteins, RNA, and bioactive substances, herbal vesicles serve as important tools for drug delivery.

In summary, nanomaterials derived from CHMs demonstrate considerable diversity in morphology, size, pharmacological activity, and sources. SHNs enhance the bioavailability of NICHMs, while CDs facilitate the integration of CHMs with medical devices, thereby expanding their application scenarios. CHM‐derived vesicles exhibit unique advantages in penetrating biological barriers, which both aids in their development as therapeutic agents and broadens the scope of nanocarriers. However, the majority of these effective CHM‐derived nanomaterials remain largely confined to the research stage. Their stability is easily compromised when preparation conditions, such as temperature, pH, and feedstock quantities, are altered. Therefore, for these nanomaterials to be developed into pharmaceuticals, their preparation processes must be stabilized and optimized for scale‐up production. Establishing robust quality standards to meet the requirements of subsequent clinical development and application is a critical area that requires further attention and effort.

## 4 Carrier‐Based Nanostructures Employed in CHMs

**Figure 4:**
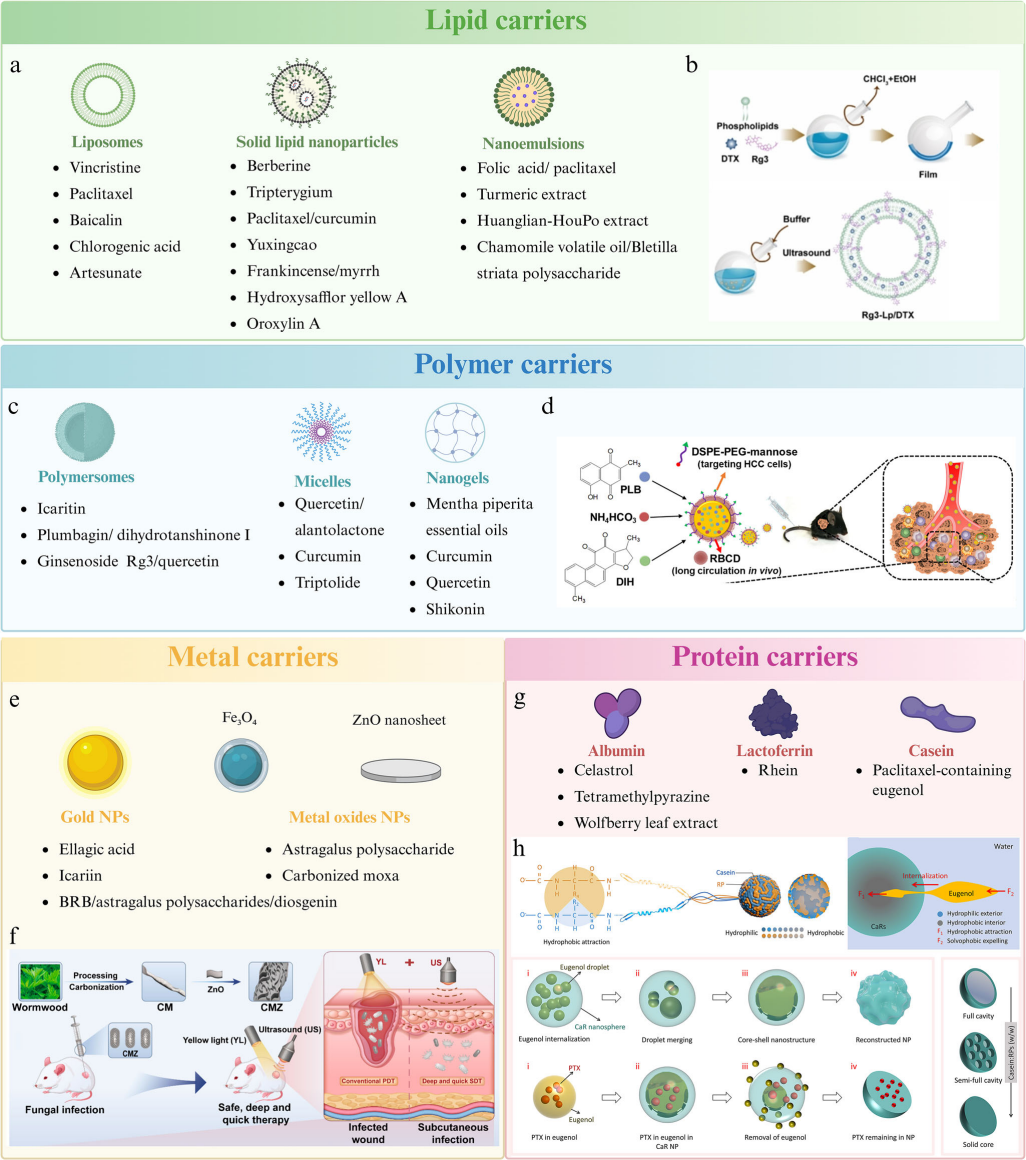
CHMs‐derived nanoparticles with diversified carrier. Lipid carriers. a) Lipid carriers mainly include liposomes, solid lipid nanoparticles, and nanoemulsions. b) A multifunctional Rg3 liposome loaded with docetaxel (Rg3‐Lp/DTX) is prepared using the thin‐film hydration method. This formulation effectively inhibits lung metastasis in triple‐negative breast cancer. Reproduced with permission.^[^ ^123^ ^]^ Copyright 2022, Science. Polymer carriers. c) Polymer carriers include polymersomes, micelles, and nanogels. d) PLGA nanoparticles are applied for co‐encapsulate plumbagin (PLB, the ICD inducer for HCC cells), Dihydrotanshinone I (DIH, the ICD enhancer by generating ROS) and NH_4_HCO_3_ (a pH sensitive adjuvant). These nanoparticles are then coated with the mannose‐inserted erythrocyte membrane to produce a nanoformulation, aimed at reversing the immunosuppressive TME in HCC. Reproduced with permission.^[^ ^124^ ^]^ Copyright 2022, Elsevier. Inorganic carriers. e) Inorganic carriers mainly include gold NPs and metal oxides NPs. f) Carbonized moxa (CM) is used to adjust the bandgap of ZnO, resulting in the formation of carbonized moxa@ZnO (CMZ). It has shown potential in the treatment of open wound and subcutaneous fungal infections. Reproduced with permission.^[^ ^125^ ^]^ Copyright 2024, ACS. Protein carriers. g) Protein carriers include albumin, lactoferrin, and casein. h) Self‐assembly NPs of casein and rice protein (CaRs) from milk and rice are loaded with eugenol. The internalization of eugenol is driven by hydrophobic attractions and solvophobic forces, which include diffusion, coalescence, formation of a core–shell structure, and removal of eugenol. Additionally, PTX can be loaded onto CaR NPs (PTX@CaRs). Reproduced with permission.^[^ ^126^ ^]^ Copyright 2023, Elsevier.

Nanocarriers exhibit diverse forms and compositions, making them suitable for delivering not only NICHMs, but also complex systems, such as CHM extracts. Commonly used nanocarriers for CHM delivery include lipid‐, polymer‐, inorganic‐, and protein‐based carriers. They offer several advantages for enhancing the efficacy of CHM, including improved permeability across biological barriers (e.g., cell membranes, tumor matrices, and BBB), sustained drug release, and enhanced targeting efficiency. Moreover, specific nanocarriers respond to environmental stimuli to enable controlled drug release. In summary, nanocarriers significantly enhanced the therapeutic efficacy of CHMs through multiple mechanisms (**Figure** **4**).

### 4.1 Lipid Carriers

Lipid‐based carriers include liposomes, solid lipid NPs (SLNs), nanostructured lipid carriers (NLCs) and nanoemulsions, composed of natural or synthetic lipids. They can encapsulate drugs with diverse properties; hydrophilic drugs are enclosed within an aqueous core, whereas hydrophobic drugs are embedded in the lipid bilayer.^[^ ^127^ ^]^ This versatility enables lipid‐based carriers to deliver hydrophilic, hydrophobic, and amphiphilic compounds effectively. Besides, these carriers demonstrate high compatibility with human tissues and typically do not provoke immune responses, resulting in excellent biocompatibility (Figure 4a). Based on these structural features, lipid‐based carriers could deliver NICHMs and CHM extracts.

#### 4.1.1 Liposomes

Liposomes,^[^ ^128^ ^]^ a prominent subset of lipid‐based carriers, are composed primarily of phospholipids.^[^ ^129^ ^]^ The use of liposomes for delivering NICHMs began in the 1980s^[^ ^130^ ^]^ with several commercially available liposome‐based drugs, such as paclitaxel.^[^ ^131^ ^]^ Relevant research is ongoing:

*Single‐NICHM delivery*: Functionalization of liposomes with targeting ligands enables drug delivery systems to achieve active targeting. A multi‐functional ginsenoside Rg3 liposome loaded with docetaxel was developed, in which ginsenoside Rg3 integrated into the phospholipid bilayer, exposing the glycosyl group on the liposome surface (Figure 4b).^[^ ^123^ ^]^ This modification results in an enhanced circulating tumor cell capture efficiency through interactions with glucose transporter 1, which was overexpressed on circulating tumor cells. In another study, an ApoE‐functionalized liposomal nanoplatform was developed for the co‐delivery of artesunate‐phosphatidylcholine and temozolomide to resistant glioblastomas, demonstrating efficient BBB traversal via low‐density lipoprotein receptor‐mediated transcytosis and achieving deep intracranial tumor penetration.^[^ ^132^ ^]^ Moreover, this system directly tethers artesunate to phosphatidylcholine, thereby addressing the stability and drug‐loading challenges of conventional liposomes.

*Multi‐NICHM delivery*: Multidrug‐loaded liposomes, encapsulating baicalin, borneol, and cholic acid, were developed to prevent ischemic stroke.^[^ ^133^ ^]^ These liposomes improve drug solubility and permeability, and deliver drugs to the brain via passive diffusion, cell membrane endocytosis, and fusion, while reducing the escape rate of encapsulated drugs. However, a major limitation of conventional liposomes is their rapid recognition by the reticuloendothelial system, which results in a short half‐life and poor stability. Fortunately, this can be addressed by employing surface modification with polyethylene glycol (PEG) to optimize the pharmacokinetic properties and extend the blood circulation time.^[^ ^134^ ^]^ Chlorogenic acid, a promising cancer immunotherapy agent, has entered phase II clinical trials in China in the form of a lyophilized powder for glioma treatment. However, its in vivo instability necessitates daily intramuscular injections, which reduces patient compliance. To overcome this issue, PEGylated liposomes containing chlorogenic acid‐phospholipid complexes have been developed.^[^ ^135^ ^]^ These PEGylated liposomes effectively inhibited tumor growth, even when the administration intervals were extended to four days, thereby reducing the required administration frequency.

#### 4.1.2 SLNs

SLNs are solid colloidal particles with sizes ranging from 10 to 1000 nm.^[^ ^136^ ^]^ They are composed of natural or synthetic solid lipids, such as lecithin and triglycerides, which serve as carrier matrices. SLNs are designed to encapsulate or embed drugs within a lipid core to form a solid lipid‐NP‐based drug delivery system. SLNs can deliver single NICHMs to overcome their limitations, such as SLN‐loaded berberine to address poor gastrointestinal absorption and low plasma levels^[^ ^137^ ^]^ and SLN‐loaded triptolide to mitigate severe liver toxicity.^[^ ^138^ ^]^ In co‐delivery applications, paclitaxel SLNs co‐loaded with curcumin demonstrated synergistic anti‐lung cancer effects both in vitro and in vivo.^[^ ^139^ ^]^ SLNs can also be used to deliver CHM mixtures. For instance, SLNs have been developed for the sustained pulmonary delivery of *Houttuynia cordata* essential oil.^[^ ^140^ ^]^ Three SLN formulations with different particle sizes (SLN‐200, SLN‐400, and SLN‐800) were prepared using Compritol 888 ATO as the lipid and polyvinyl alcohol as the emulsifier, achieving fine particle fractions of 67.4% to 75.8% after nebulization. Similarly, other studies have used SLNs to co‐deliver frankincense and myrrh oils for oral administration, targeting the anti‐tumor efficacy.^[^ ^141^ ^]^ When conventional SLNs fail to fully meet application requirements, they were be optimized. For example, hydroxysafflor yellow A SLNs with a water‐in‐oil‐in‐water structure were prepared using a warm microemulsion process for oral delivery, with the aim of enhancing the absorption of hydroxysafflor yellow A.^[^ ^142^ ^]^ The final optimal formulation contains 0.3 g of hydroxysafflor yellow A, 150 mg of hydroxypropyl methyl cellulose, 5.5 mL of caprylic/capric triglyceride 1.5 mL of Labrafil M 1944 CS and 2.5 g of glyceryl monostearate. The optimized SLNs were spherical, with an average size of 214 nm and an encapsulation efficiency of 55%.

#### 4.1.3 NLCs

NLCs,^[^ ^143^ ^]^ which are composed of a mixture of solid and liquid lipids, represent the second generation of lipid NP‐based drug delivery systems. They combine the advantageous properties of SLNs while addressing their limitations, such as low drug‐loading capacity, drug leakage during storage, and the risk of gelation. A study comparing the microstructure and transdermal delivery characteristics of alkaloids extracted from *Aconitum sinomontanum* loaded into NLCs and SLNs revealed that NLC‐associated alkaloids exhibited greater cumulative skin penetration and a higher area under the concentration‐time curve (AUC)_0‐t_ compared with those of SLN‐associated alkaloids.^[^ ^144^ ^]^ In another study, oroxylin A loaded into NLCs reduced the UV‐induced oxidative stress in the skin.^[^ ^145^ ^]^ Subsequently, oroxylin A‐NLCs were incorporated into a hydrogel matrix to facilitate their application on the dorsal skin of mice.

#### 4.1.4 Nanoemulsions

Nanoemulsions, composed of oil and water encapsulate hydrophobic drugs and form nanoscale emulsions.^[^ ^146^ ^]^ Nanoemulsions can be classified as either oil‐in‐water (O/W) or water‐in‐oil, with O/W emulsions being more commonly used in the delivery of NICHMs or CHM extracts.

*NICHM delivery*: Folic acid‐decorated nanoemulsions co‐delivering paclitaxel and docosahexaenoic acid (1:5) demonstrated potential in cancer therapy by targeting folate receptors, with controlled drug release within 48 h and no burst effects.^[^ ^147^ ^]^

*CHM extract delivery*: Nanoemulsions are increasingly used to deliver CHM‐derived mixtures. A turmeric extract nanoemulsion (containing 59% curcumin, 22% demethoxycurcumin, and 18% bisdemethoxycurcumin) was developed using an emulsification method and photomicrographs revealed smooth, spherical droplets that enhanced its antidepressant effect.^[^ ^148^ ^]^ The *Coptis Root*‐*Officinal Magnolia Bark* extract nanoemulsion, developed for UC treatment, had a droplet size of 65.21 ± 0.82 nm with six phytochemicals (berberine, epiberberine, coptisine, bamatine, magnolol, and honokiol).^[^ ^149^ ^]^ These particles were nearly spheroidal with a brownish‐yellow, milky appearance, demonstrating good stability and controlled release of phytochemicals in simulated gastric and intestinal fluids, effectively withstanding the harsh conditions of the digestive tract. Additionally, for topical applications, nanoemulsions can be incorporated into gels to reduce their flowability. Chamomile volatile oil nanoemulsions were prepared using the phase‐transition method, yielding spherical NPs with a particle size of 19.07 nm, which were then encapsulated in *Bletilla striata* polysaccharides to form chamomile volatile oil nanoemulsion gels for the treatment of atopic dermatitis.^[^ ^150^ ^]^ In this system, *Bletilla striata* polysaccharide exhibited moisturizing properties by forming a uniform film on the skin surface.

### 4.2 Polymer Carriers

Polymeric nanocarriers,^[^ ^151^ ^]^ synthesized from natural or synthetic materials in the form of monomers or polymers, can encapsulate various NICHMs and efficiently release them in response to internal or external stimuli.^[^ ^152^ ^]^ Polymersomes, micelles, and nanogels are commonly used as polymeric nanocarriers to deliver CHMs (Figure 4c).

#### 4.2.1 Polymersomes

Polymersomes^[^ ^153^ ^]^ are constructed from synthetic or naturally derived polymeric materials designed to encapsulate NICHMs within their cores or covalently attach functional groups to them. Poly(D, L‐lactic‐co‐glycolic acid)(PLGA)‐PEG‐aminoethyl anisamide NPs co‐delivering icaritin and doxorubicin remodeled the immunosuppressive tumor microenvironment and triggered a robust immune memory response in a hepatocellular carcinoma mouse model.^[^ ^154^ ^]^ Meanwhile, polymersomes can be modified to satisfy specific delivery requirements. Plumbagin and dihydrotanshinone I were co‐encapsulated into PLGA NPs with NH₄HCO₃ for pH‐sensitive release (Figure 4d).^[^ ^124^ ^]^ In addition, coated with red blood cell vesicles and 1,2‐distearoyl‐sn‐glycero‐3‐phosphoethanolamine (DSPE)‐PEG_2000_‐mannose, these polymersomes can target hepatocellular carcinoma cells, enhancing the drug half‐life and tumor targeting in mice. In another study, folate‐targeted PEG‐modified amphiphilic cyclodextrin NPs were developed to co‐encapsulate ginsenoside Rg3 and quercetin, prolong blood circulation, and improve tumor targeting in an orthotopic colorectal cancer mouse model.^[^ ^155^ ^]^

#### 4.2.2 Micelles

Micelles are formed by the self‐assembly of amphiphilic block copolymers in aqueous solutions above a critical micelle concentration, typically featuring a hydrophobic core and hydrophilic shell.^[^ ^156^ ^]^ These carriers were commonly used to deliver NICHMs. Long‐circulating micellar particles were used to co‐deliver quercetin and alantolactone at a molar ratio of 1:4, considering the hydrophobic nature of both drugs.^[^ ^157^ ^]^ The micelle formulation consisted of two FDA‐approved polymers, TPGS and DSPE‐PEG2000, which are known to be safe adjuvants. A hydroxyethyl starch‐curcumin conjugate was synthesized via the esterification of hydroxyethyl starch and monocarboxylic‐terminated curcumin.^[^ ^158^ ^]^ With an optimal hydrophilic‐to‐hydrophobic ratio, the conjugate self‐assembled into transparent, acid‐responsive micelles that exhibited enhanced antioxidant and anticancer activities compared with those of a heterogeneous free curcumin solution. Another pH‐sensitive micelle for cancer therapy was developed to co‐deliver chlorin e6 and triptolide.^[^ ^159^ ^]^ The system formed flower‐like micelles through mPEG‐poly(β‐amino ester) (PBAE)‐mPEG self‐assembly, with a PEG shell, a PBAE core, and a β‐cyclodextrin layer to prevent premature drug leakage. After accumulating in tumor tissues via the enhanced permeability and retention effect (EPR) effect, the acidic microenvironment triggered controlled drug release.

#### 4.2.3 Nanogels

Nanogels^[^ ^160^ ^]^ are three‐dimensional polymer networks formed through physical or chemical crosslinking that combine the properties of hydrogels^[^ ^161^ ^]^ and NPs, such as a modifiable large surface area, water swelling, adhesion, high drug‐loading capacity, and sustained release, and are classified as conventional or environmentally responsive based on their phase transition mechanisms.^[^ ^162^ ^]^ Chitosan is one of the most widely used polysaccharides for the development of drug carriers.^[^ ^163^ ^]^ These cationic polymers, derived from the deacetylation of chitin to chitosan of varying molecular weights, readily bind to negatively charged compounds through electrostatic interactions, eliminating the need for chemical reactions or extensive modifications. These nanocarriers could be used to deliver both extracts and NICHMs. A chitosan nanogel, synthesized via the sol–gel method using tripolyphosphate as a crosslinking agent, was loaded *Mentha piperita* essential oils, which exhibited inhibitory effects on biofilm formation by *Streptococcus mutans* on dental surfaces.^[^ ^164^ ^]^ A different chitosan nanogel was integrated with poly(*N*‐isopropylacrylamide) for thermo‐responsive delivery of curcumin.^[^ ^165^ ^]^ In this system, poly(*N*‐isopropylacrylamide) acts as a thermally sensitive polymer with a low critical solution temperature of 32 °C, making it particularly suitable for biomedical applications because of its proximity to the body temperature. Another type of thermosensitive hydrogel was prepared using poloxamers^[^ ^166^ ^]^ to co‐deliver quercetin and brain‐derived neurotrophic factor for depression therapy.^[^ ^167^ ^]^ The gelation temperature, suitable for intranasal delivery, was ≈30.3 °C when the ratio of poloxamer 407 to poloxamer 188 was close to 16:2. This nanogel system exhibited a flowing state at 25 °C but transitioned to a non‐flowing state at 37 °C. Additionally, environmentally responsive nanogels can be functionalized for targeted delivery. A sarcoma‐targeting peptide‐modified, reduction‐responsive poly(ethylene glycol)‐poly(L‐phenylalanine‐co‐L‐cysteine) nanogel was designed for the targeted intracellular delivery of shikonin to induce osteosarcoma necroptosis and reduce pulmonary metastasis.^[^ ^168^ ^]^

### 4.3 Inorganic Carriers

Compared with organic NPs, inorganic NPs offer superior chemical stability and exhibit unique optical, magnetic, catalytic, and electrical properties.^[^ ^169^ ^]^ Common inorganic nanocarriers used for NICHM delivery include metallic and oxide nanocarriers (Figure 4e).

#### 4.3.1 Gold NPs (AuNPs)

AuNPs^[^ ^170^ ^]^ exhibit unique properties, including structural diversity and the ability to load NICHMs via non‐covalent interactions or covalent conjugation.^[^ ^171^ ^]^ NICHMs can also effectively serve as reductants in the synthesis of AuNPs for therapeutic applications, eliminating the need for hazardous chemicals. One study presented an efficient method for synthesizing Au‐phenolic core‐shell NPs with a high loading capacity using phenolic compounds, such as epicatechin, catechin, taxifolin, gallic acid, and ellagic acid.^[^ ^172^ ^]^ Ellagic acid‐AuNPs exhibit excellent dispersibility, biocompatibility, and uniform quasi‐spherical shapes with smooth surfaces. Encased in a light‐gray layer, indicative of a thin organic coating, these AuNPs possessed a face‐centered cubic structure with core diameters predominantly ranging from 20 to 40 nm. In another study, multilayer core–shell AuNPs enhanced with ellagic acid showed improved biocompatibility and bioactivity, effectively mitigating myocardial infarction injury while supporting metabolism via desirable excretion pathways without overburdening other organs.^[^ ^173^ ^]^ Functional icariin‐loaded selenium‐Au multishell nanocomposites have been designed for synergistic therapeutic effects.^[^ ^174^ ^]^ Upon near‐infrared light irradiation, the AuNPs generated a significant photothermal effect, inducing the release of selenium NPs and icariin, which effectively inhibited the production of inflammatory factors and accumulation of ROS. AuNPs range in size from 5 to 100 nm, which may impact the intrinsic efficiency of the drug they carry owing to the potential steric hindrance posed by larger materials.^[^ ^175^ ^]^ In contrast, Au nanoclusters, with sizes of ≈2 nm and composed of a small number of atoms, are negligible in size compared to NICHMs. Researchers have synthesized well‐distributed Au nanoclusters incorporating berberine, *Astragalus* polysaccharides, and diosgenin, with an average size of less than 3 nm.

#### 4.3.2 Metal Oxides NPs

Iron (Fe) oxides have diverse biomedical applications owing to their biocompatibility, oxidation resistance, and magnetic properties. Ultra‐small superparamagnetic iron oxide (USPIO) NPs are metabolized in lysosomes into a soluble, non‐magnetic form of Fe, which becomes part of the body's normal iron pool following intracellular uptake.^[^ ^176^ ^]^ Ferumoxytol, a representative USPIO NP, was approved by the FDA in 2009 for treating Fe‐deficiency anemia in adults with chronic kidney disease.^[^ ^177^ ^]^ Additionally, among USPIO NPs, Fe₃O₄ NPs exhibit strong superparamagnetic properties and are promising candidates for use as both an Fe supplement and magnetic resonance imaging contrast agent.^[^ ^178^ ^]^ A novel core‐shell Fe₃O₄ NP material modified with *Astragalus* polysaccharide was developed for treating iron deficiency anemia.^[^ ^179^ ^]^ Fabricated through hydrothermal synthesis and esterification, the water‐soluble Fe₃O₄ NPs had a hydrodynamic diameter of 11 nm, while *Astragalus* polysaccharide‐Fe₃O₄ NPs averaged 29.5 nm, confirming the NICHM coating. In rat models, this material displayed therapeutic effects by combining Fe supplementation with *Astragalus* polysaccharide‐induced hematopoietic cell generation.

ZnO, a classic photoresponsive material, is one of the few antimicrobials approved by the FDA.^[^ ^180^ ^]^ When synthesized at the nanoscale, ZnO NPs exhibit significant antibacterial activity by interacting with the outer bacterial layers and cores through various bactericidal mechanisms.^[^ ^181^ ^]^ *Moxa*, a CHM with excellent biocompatibility and the ability to adjust the bandgap of ZnO, was carbonized and modified with ZnO nanosheets to form carbonized moxa‐ZnO, which exhibited dual responsiveness to yellow light and ultrasound for synergistic antifungal therapy (Figure 4f).^[^ ^125^ ^]^ Prepared via low‐temperature carbonization, the carbonized moxa displayed a flat band structure with regular grooves, and its surface was coated with a wrinkled ZnO nanosheet layer, resulting in a porous, carbonized moxa‐ZnO material.

### 4.4 Protein‐Based Carriers

Proteins are naturally occurring biomacromolecules that exhibit high biocompatibility and are rapidly degraded by enzymes into amino acids and peptides. These breakdown products are safely metabolized and excreted from the body, thereby minimizing the risk of toxicity. They are less likely to be cleared by macrophages, making them a promising foundation for the development of drug carriers with a prolonged circulation time. Thus, protein‐based nanocarriers show substantial potential for CHM delivery (Figure 4g).^[^ ^182^ ^]^

Albumin^[^ ^183^ ^]^ is a widely used protein carrier in drug delivery systems because of its ability to bind to various lipophilic drugs, extend circulation time in the bloodstream, and reduce drug‐related side effects. Inspired by the success of Abraxane, an albumin‐bound NP formulation of doxorubicin used in cancer therapy, recent advancements in protein‐based nanomedicine have enabled the development of novel treatments. For example, a study demonstrated that celastrol‐loaded albumin NPs effectively targeted mesangial cells to treat mesangioproliferative glomerulonephritis.^[^ ^184^ ^]^ In addition, human serum albumin NPs with a size of 95 nm maximize mesangial cell uptake through caveolae‐and clathrin‐mediated pathways and macropinocytosis. These albumin NPs can deliver high concentrations of celastrol to mesangial cells and prolong drug availability at the target site. In another study, HIV‐1‐activated transcription factor‐modified serum albumin NPs were developed for the sustained release of tetramethylpyrazine, and their ability to target spinal cord injuries was enhanced.^[^ ^185^ ^]^ In this system, spherical light‐blue NPs were prepared using the emulsification‐dispersion technique, with an encapsulation efficiency achieved when 30 mg of tetramethylpyrazine was used. What's more, to enhance the stability and bioavailability of polyphenols, wolfberry leaf extract was encapsulated in NPs composed of whey protein isolate and bovine serum albumin via self‐assembly.^[^ ^186^ ^]^ Inside them, phenolics were embedded within the proteins, forming spherical bovine serum albumin‐wolfberry leaf nanocomposites. These nanocomposites exhibited a sparse spherical outer structure with a dense inner core, where hydrophobic amino acids formed the core, and hydrophilic groups on the surface underwent hydrophobic cross‐linking under potentiostatic resistance during self‐assembly.

Lactoferrin,^[^ ^187^ ^]^ a glycoprotein of the transferrin family, functions as a nutrient carrier that transfers iron to cells, and specifically binds to low‐density lipoprotein receptor‐related proteins expressed on inflammatory macrophages.^[^ ^188^ ^]^ Moreover, lactoferrin interacts with its receptors, which are highly expressed in intestinal epithelial cells, making it a promising candidate for drug delivery in UC treatment. Based on this, a dual‐targeting lactoferrin nanoparticle system modified with calcium pectinate and hyaluronic acid, and loaded with Rhein was developed.^[^ ^189^ ^]^ In this nanosystem, calcium pectinate/hyaluronic acid/Rhein NPs were prepared using the dialysis technique, and the formation of three‐layered NPs was driven by electrostatic adsorption and cross‐linking reactions. Another protein‐based nanocarrier, casein,^[^ ^190^ ^]^ is a cheap agricultural product derived from milk with annual production. Self‐assembling NPs prepared from casein and rice proteins exhibited customizable release profiles.^[^ ^126^ ^]^ Paclitaxel‐containing eugenol diffused into the hydrophobic core of these NPs, and subsequent dialysis removed the eugenol, resulting in a sculpted core structure. As the mass ratio of casein to rice protein increased, the NPs became more structurally solid, enhancing tumor growth inhibition in animal models (Figure 4h).

In summary, the diversity of nanocarriers has a huge potential to enhance the performance of CHMs from multiple perspectives (Table 2). Specifically, lipid carriers are capable of delivering both small molecules and extracts from CHMs, and exhibit excellent biocompatibility and biodegradability. Their physicochemical properties can be tailored by changing their lipid composition and they can be functionally modified to achieve active targeting. Polymer carriers are predominantly employed for the delivery of NICHMs and facilitate environmentally responsive drug release. Inorganic carriers, such as metallic and metal oxide NPs, offer a high specific surface area and stability when delivering NICHMs and are applicable for photothermal therapy and magnetic targeting. Protein carriers can be used to deliver both NICHMs and CHM extracts. They can self‐assemble or co‐assemble into nanocarriers through simple preparation processes. At the same time, they are less likely to be cleared by macrophages and have the potential to prolong circulation time. They also possess intrinsic targeting capabilities and bind to specific cell surface receptors. The specific aspects by which these versatile nanocarriers confer benefits for the delivery of CHMs or NICHMs are discussed in the following section.

**Table 2:** The comparison of different nano‐strategies applied for CHMs.

Strategies	Types of nanomaterials		Advantages	Limitations	Application for delivery	Optimization of CHMs	Marketed drugs
Nanocarrier‐free strategy	SHNs	Natural SHNs	Natural sources, multi‐component synergistic effect, simple preparation and 100% high drug loading	Complex composition, low stability, difficult quality control, and size heterogeneity	CHMs (QY305 decoction,^[^ ^48^ ^]^ Qingxuechushi mixture,^[^ ^63^ ^]^ Danggui Buxue decoction,^[^ ^64^ ^]^ etc.)	New insight into the material basis of CHMs	Not applicable
		Artifitial SHNs	Natural sources, multi‐component synergistic effect, well‐defined composition, size‐controllable, simple preparation and 100% high drug loading	Low stability, unclear mechanisms of self‐assembly process, and limited compounds found with the ability to self‐assemble	NICHMs (Berberine, Rhein, glycyrrhizic acid, licorice protein, lentinan, celastrol oleanolic acid, curcumin, etc.)	Improvement of bioavailability, facilitation of target effect, decrease of toxicity, and achievement of drug release	Not applicable
	CDs	–	Bioimaging capabilities, size‐controllable, green synthesis and environmental sustainability	Heterogeneous distribution and limitations in retention of active ingredients	CHMs (*Jujube*,^[^ ^87^ ^]^ *Platyclad cacumen*,^[^ ^88^ ^]^ donkey‐hide gelatin,^[^ ^89^ ^]^ safflower and angelica,^[^ ^90^ ^]^ honeysuckle, taxus leaves and dandelion.[^91^ ^]^ *Persicae semen* and *Carthami flos*,^[^ ^93^ ^]^ Typhae pollen,^[^ ^94^ ^]^ honeysuckle,^[^ ^92^ ^]^ watermelon rind,^[^ ^95^ ^]^ ginger,^[^ ^97^ ^]^ Gynostemma,^[^ ^98^ ^]^ etc.); NICHMs (Glycyrrhizic acid,^[^ ^99^, ^100^ ^]^ ginsenoside,^[^ ^101^ ^]^ Rhein,^[^ ^102^ ^]^ etc.)	New insight into the material basis of carbonized CHMs and acquiring bioimaging functions	Not applicable
	Vesicle‐like NPs	–	Intrinsic targeting capability	Low stability	Fresh CHMs (Ginger,^[^ ^107^, ^108^, ^109^, ^119^ ^]^ garlic,^[^ ^110^, ^116^ ^]^ Pueraria,^[^ ^111^ ^]^ turmeric,^[^ ^112^ ^]^ lemon,^[^ ^114^, ^115^ ^]^ ginseng,^[^ ^117^, ^118^, ^122^ ^]^ *Dandelion*,^[^ ^120^ ^]^ *B. javanica*,^[^ ^121^ ^]^ etc.)	New insight into the material basis of fresh CHMs	Not applicable
Nanocarrier‐based strategy	Lipid carriers	Liposomes	Capable of loading both hydrophilic and lipophilic drugs, excellent biocompatibility, and good biodegradability	Time‐consuming and costly preparation, low stability	NICHMs (Ginsenoside Rg3,^[^ ^123^ ^]^ artesunate,^[^ ^132^ ^]^ baicalin, borneol, and cholic acid,^[^ ^133^ ^]^ chlorogenic acid,^[^ ^135^ ^]^ etc.)	Increasing solubility, enhancing permeability, decrease of toxicity, and environmental responsive	Lipusu Camptosar Marqibo Onivyde
		SLNs			NICHMs (Berberine,^[^ ^137^ ^]^ triptolide,^[^ ^138^ ^]^ paclitaxel and curcumin,^[^ ^139^ ^]^ hydroxysafflor yellow A,^[^ ^142^ ^]^ etc.); CHM extract (*Houttuynia cordata* essential oil,^[^ ^140^ ^]^ frankincense and myrrh oils,^[^ ^141^ ^]^ etc.)	Enhancing the absorption and permeability, and decrease of toxicity	Not applicable
		NLCs			NICHMs (Oroxylin A^[^ ^145^ ^]^) CHM extract (Alkaloids extracted from *Aconitum sinomontanum* ^[^ ^144^ ^]^);	Enhancing the absorption and permeability	
		Nanoemulsions			NICHMs (Paclitaxel^[^ ^147^ ^]^); CHM extract (Turmeric extract,^[^ ^148^ ^]^ *Coptis Root*‐*Officinal Magnolia Bark* extract,^[^ ^149^ ^]^ Chamomile volatile oil,^[^ ^150^ ^]^ etc.)	Enhancing stability and controlled release	Not applicable
	Polymer carriers	Polymersomes	Enhanced drug stability, targetable drug delivery via surface modification, and stimulus‐responsive release	Some polymers exhibit cytotoxicity issues	NICHMs (Icaritin,^[^ ^154^ ^]^ plumbagin and dihydrotanshinone I,^[^ ^124^ ^]^ ginsenoside Rg3 and quercetin,^[^ ^155^ ^]^ etc.)	Prolonging the half‐life (*t*_1/2_), facilitation of target effect, and environmental responsive	Not applicable
		Micells			NICHMs (quercetin and alantolactone,^[^ ^157^ ^]^ curcumin,^[^ ^158^ ^]^ triptolide,^[^ ^159^ ^]^ etc.)	Increasing solubility, prolonging the half‐life (*t*_1/2_), decrease of toxicity, and environmental responsive	Genexol‐PM Apealea
		Nanogels			NICHMs (Curcumin,^[^ ^164^ ^]^ quercetin,^[^ ^167^ ^]^ shikonin,^[^ ^168^ ^]^ etc.); CHM extract (*Mentha piperita* essential oils^[^ ^164^ ^]^)	Decrease of toxicity, environmental responsive, high drug‐loading capacity, and sustained release	Not applicable
	Inorganic carriers	Metal NPs	High specific surface area, drug‐loading capacity, and stability, multifunctionality, good biocompatibility, and unique optical properties suitable for imaging and photothermal therapy	Risk of in vivo accumulation, unclear long‐term toxicity	NICHMs (Ellagic acid,^[^ ^172^, ^173^ ^]^ selenium,^[^ ^174^ ^]^ berberine, *Astragalus* polysaccharides, and diosgenin,^[^ ^175^ ^]^ etc.)	Photothermal therapy	Not applicable
		Metal Oxides NPs			NICHMs (*Astragalus* polysaccharide^[^ ^179^ ^]^); CHMs (Moxa^[^ ^125^ ^]^)	Fe oxides as both a Fe supplement and magnetic resonance imaging contrast agent; ZnO, as both a classic photoresponsive material and an antimicrobial	Not applicable
	Protein carriers		Intrinsic targeting capability, good biodegradability, and excellent biocompatibility	Low stability	CHM extract (wolfberry leaf extract^[^ ^186^ ^]^); NICHMs (Paclitaxel,^[^ ^126^ ^]^ celastrol,^[^ ^184^ ^]^ tetramethylpyrazine,^[^ ^185^ ^]^ Rhein,^[^ ^189^ ^]^ etc.)	Facilitation of target effect, enhancing the absorption and prolong drug availability at the target site, enhance the stability	Abraxane

## 5 Advantages of Pharmaceutical Nanotechnology Applied in CHMs

The relatively mild therapeutic efficacy of CHMs is largely attributed to their poor solubility, limited permeability, low stability, rapid elimination, weak targeting ability, and extensive metabolism, which restricts their clinical application.^[^ ^191^ ^]^ Fortunately, nanomaterials characterized by distinctive physical and chemical properties, including high surface area, quantum effects, and size‐dependent behavior, offer promising solutions to these obstacles.^[^ ^192^ ^]^ In the following sections, we explore the benefits of carrier‐based and carrier‐free nanostrategies in enhancing the therapeutic efficacy of CHMs from four perspectives: improvement of bioavailability, facilitation of target effects, decrease in toxicity, and achievement of controlled release.

### 5.1 Improvement of Bioavailability

Enhancing the bioavailability of CHMs is essential for improving their therapeutic efficacy. Some CHMs, such as *Tripterygium wilfordii*,^[^ ^193^ ^]^ are primarily composed of lipophilic ingredients that inherently limit their solubility in water. Similarly, many NICHMs, particularly flavonoids and terpenoids, contain hydroxyl and carboxyl functional groups. While these groups facilitate hydrogen bond formation, they also reduce water solubility by increasing the intermolecular interactions and lattice energy. Moreover, the stability of the components of CHMs affects their bioavailability. Nanotechnology‐based strategies can enhance the bioavailability of CHMs from multiple perspectives, including improving the water solubility and stability of active components, as well as prolonging their half‐lives. These aspects are described in detail in the following sections.

#### 5.1.1 Bioavailability Improved of Carrier‐Free Nanostructures

**Figure 5:**
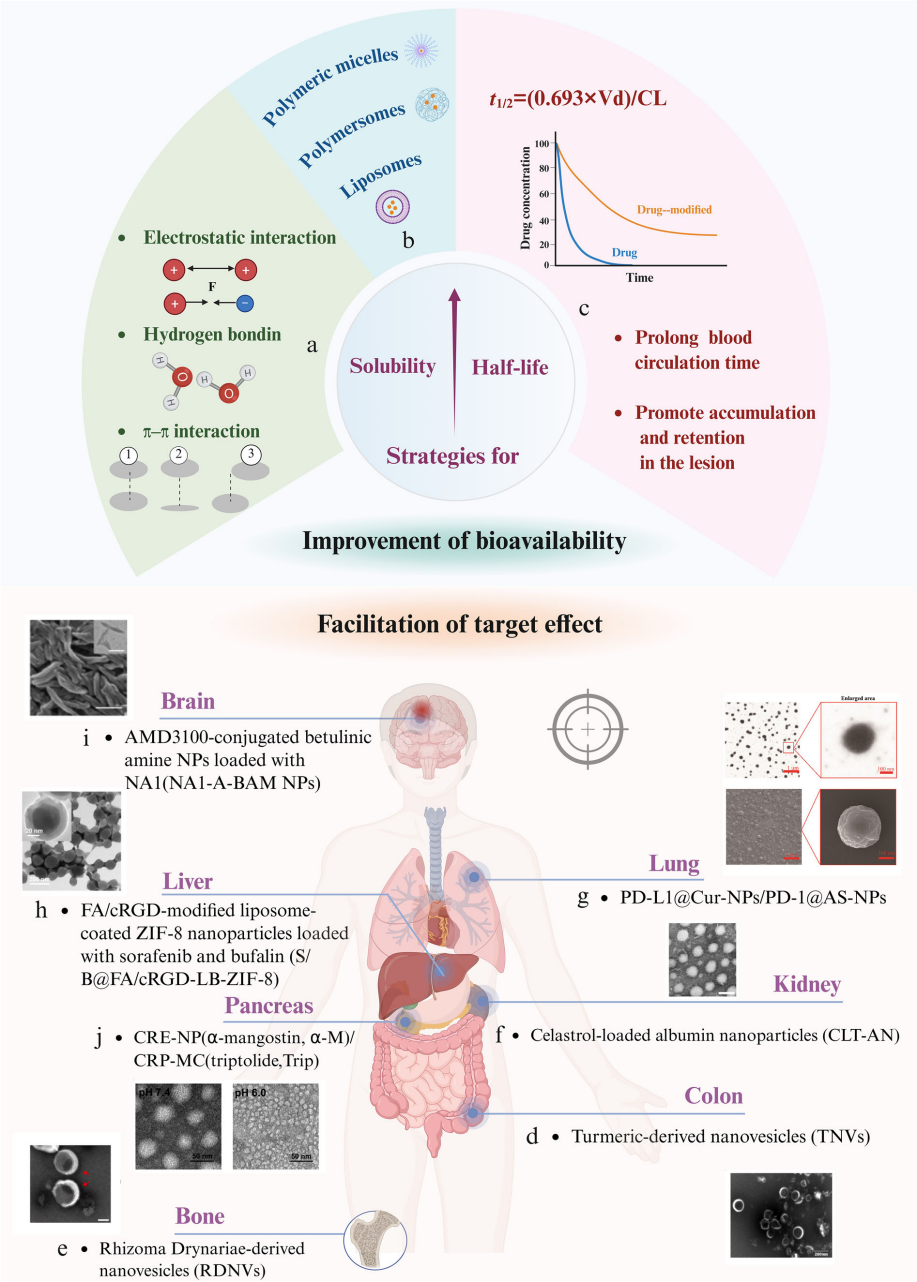
Improvement of bioavailability and targeting effects of CHMs via nanotechnology. Strategies to improve bioavailability mainly include increasing drug solubility and extending the drug's half‐life. a) One method to increase solubility involves the use of π–π stacking, electrostatic forces, and hydrogen bonding intermolecular interactions. b) Another method to increase solubility is by using carriers such as liposomes, polymeric micelles, and polymersomes, which help increase the bioavailability of hydrophobic small molecules. c) Increasing a drug's half‐life also improves bioavailability by prolonging its circulation time in the bloodstream, which enhances drug accumulation and retention at the target site. Targeted therapeutic strategies. d) Turmeric‐derived nanovesicles (TNVs) are produced to improve colitis by restoring the damaged intestinal barrier, modulating the gut microbiota and reprogramming the macrophage phenotype. Reproduced with permission.^[^ ^112^ ^]^ Copyright 2022, Ivyspring. e) *Rhizoma Drynariae*‐derived nanovesicles (RDNVs) are developed for bone tissue‐targeting, promoting the proliferation and osteogenic differentiation of human bone marrow stem cells (hBMSCs) to reverse osteoporosis. Reproduced with permission.^[^ ^202^ ^]^ Copyright 2024, Elsevier. F) Celastrol‐albumin nanoparticles (CLT‐AN) provide an exciting treatment strategy for mesangioproliferative glomerulonephritis by targeting mesangial cells. Reproduced with permission.^[^ ^184^ ^]^ Copyright 2017, Nature. g) PLGA nanoparticles, loaded with curcumin and astragaloside IV, are decorated with PD‐L1/PD‐1 antibody respectively. PD‐L1@Cur‐NPs precisely target lung tumour cells, while PD‐1@AS‐NPs regulate T cells in vivo. Reproduced with permission.^[^ ^204^ ^]^ Copyright 2024, Wiley. h) Zeolitic imidazolate framework‐8 (ZIF‐8) nanoparticles, loaded with bufalin (BFL) and sorafenib (SFN), are coated with FA/cRGD‐modified liposome (S/B@FA/cRGD‐LB‐ZIF‐8), creating a dual‐targeted nanoplatform for liver tumors and blood vessels. Reproduced with permission.^[^ ^205^ ^]^ Copyright 2024, Elsevier. i) A‐BAM NPs, consisting of betulinic amine conjugated with AMD3100 (a CXCR4 antagonist), demonstrate therapeutic effects for stroke. These effects are further enhanced when NA1 is encapsulated. Reproduced with permission.^[^ ^206^ ^]^ Copyright 2022, KeAi. j) A biodegradable polymer nanoparticle, modified with the CREKA peptide and loaded with α‐mangostin (CRE‐NP(α‐M)), targets cancer‐associated fibroblasts. Additionally, a low pH‐triggered micelle, coated with CRPPR peptide and loaded with triptolide (CRP‐MC(Trip)), enhances the therapeutic effect while minimizing organ toxicity. The combination of CRE‐NP(α‐M) pretreatment with CRP‐MC(Trip) shows a powerful antitumor effect in pancreatic tumor model. Reproduced with permission.^[^ ^207^ ^]^ Copyright 2020, Elsevier.

Carrier‐free CHM‐derived NPs primarily enhanced bioavailability by improving solubility (**Figure** **5**a–c). By forming NPs, NICHMs increase the surface area of the compounds and enhance their interaction with water molecules, thereby improving their solubility. Furthermore, certain NICHMs can self‐assemble via noncovalent interactions, including π–π stacking, electrostatic forces, hydrogen bonding, and coordination interactions. These processes promote intermolecular interactions, allowing the insoluble components to form stable NPs in solution. For example, berberine can self‐assemble into structurally stable NPs with components, such as magnolol,^[^ ^69^ ^]^ tannic acid,^[^ ^73^ ^]^ chlorogenic acid,^[^ ^74^ ^]^ and hesperetin.^[^ ^75^ ^]^ These processes were primarily driven by electrostatic attraction between the nitrogen cations of berberine and the ionized hydroxyl groups of other compounds, subsequently coupled with intermolecular π–π stacking, resulting in an increase in its oral bioavailability. Additionally, some NICHMs, such as glycyrrhizic acid, exhibit amphiphilic properties that enhance the solubility of other poorly soluble components. During the boiling process of the decoction, these amphiphilic molecules self‐assemble into NPs, where their hydrophobic regions interact with poorly soluble compounds and hydrophilic regions aid in dispersion in water. For instance, glycyrrhizic acid can self‐assemble into micelles with lipophilic components, such as tanshinone IIA,^[^ ^77^ ^]^ baicalin,^[^ ^78^ ^]^ and norcantharidin.^[^ ^79^ ^]^ It can also co‐assemble with Rhein and berberine to form multi‐component hydrogels.^[^ ^56^ ^]^ The formation increases the effective drug concentration.

#### 5.1.2 Bioavailability Improved of Carrier‐Based Nanostructures

In addition to inheriting the advantages of nanostructures, carriers can offer unique benefits by enhancing the bioavailability of CHMs (Figure 5a–c).

*Increasing solubility*: Liposomes improve the solubility of hydrophobic molecules, such as quercetin,^[^ ^194^ ^]^ ganoderic acid,^[^ ^195^ ^]^ patchouli alcohol,^[^ ^196^ ^]^ and camptothecin^[^ ^197^ ^]^ by encapsulating these compounds within the non‐polar regions of their phospholipid bilayers. Another method involves encapsulation of hesperidin and lenalidomide in mPEG‐PLA‐based polymeric micelles conjugated to polyethylene glycol as acid‐activated prodrugs.^[^ ^198^ ^]^ These micelles were then incorporated into a chitosan/β‐sodium glycerophosphate hydrogel, enhancing the water solubility of hesperidin.

*Prolonging the half‐life (t_1/2_)*: A self‐assembled amphiphilic drug, conjugated camptothecin‐floxuridine forms liposome‐like nanocapsules for effective cancer chemotherapy, with 20 mol% DSPE‐PEG 2000 enhancing hydration to reduce macrophage recognition and prolong blood circulation time.^[^ ^199^ ^]^ The clinical application of tanshinone IIA is limited by its high lipophilicity, low cellular uptake, and short half‐life (*t*_1/2_) of 44 min.^[^ ^200^ ^]^ To address these challenges, a mixed micelle system composed of d‐α‐tocopheryl polyethylene glycol succinate‐graft‐poly(d,l‐lactide‐co‐glycolide) copolymer and Pluronic F68 significantly prolonged the circulation time of tanshinone IIA and improved its bioavailability in pharmacokinetic studies conducted on rats.^[^ ^201^ ^]^

*Enhancing permeability*: Nanocarriers can also help NICHMs overcome permeation barriers, such as the BBB. An ApoE‐functionalized liposomal nanoplatform incorporating artesunate‐phosphatidylcholine and encapsulating temozolomide was developed to traverse the BBB via low‐density lipoprotein receptor‐mediated transcytosis and achieve deep intracranial tumor penetration.^[^ ^132^ ^]^

### 5.2 Facilitation of Target Effect

CHM‐derived nanostructures can achieve passive targeting owing to their nano size. In contrast, by modification with nanocarriers or specific targeting ligands, such as antibodies, ligands, or small molecular recognition agents, these structures can actively bind to receptors in diseased tissues, enabling active targeting.

#### 5.2.1 Target Facilitated of Carrier‐Free Nanostructures

The targeting capabilities of these nanomaterials primarily stem from the EPR effect facilitated by their nano size. For instance, turmeric‐derived vesicles showed potential for targeted therapy of UC, with drug concentrations peaking in the colonic tissues of mice 6 h after oral administration (Figure 5d).^[^ ^112^ ^]^ Celastrol and erianin self‐assemble into nanodrugs for breast cancer treatment^[^ ^81^ ^]^ Following the intravenous injection of these NPs, the EPR effect resulted in improved targeting and accumulation at tumor sites, and activity‐based protein profiling was used to identify annexin A2 as the target of celastrol in 4T1 cells. Additionally, the chemical composition of nanomaterials can affect their targeting abilities. *Rhizoma Drynariae*‐derived vesicles were developed for the treatment of postmenopausal osteoporosis, with naringin playing a key role in enhancing the osteogenic differentiation of human bone marrow mesenchymal stem cells by targeting estrogen receptor‐alpha (Figure 5e).^[^ ^202^ ^]^

#### 5.2.2 Target Facilitated of Carrier‐Based Nanostructures

The targeting capabilities of carrier‐based nanomaterials are primarily derived from the synergistic effects of the functional modifications of nanocarriers, chemical structures of natural compounds, and EPR effect, with functional carriers playing a dominant role. Carriers can achieve active targeting by using various designs. For example, albumin NPs loaded with celastrol effectively targeted the mesangial cells by crossing the fenestrated endothelium and accumulating in these cells, demonstrating their therapeutic efficacy against mesangioproliferative glomerulonephritis (Figure 5f).^[^ ^184^ ^]^ Similarly, a targeted delivery system using peptide‐coupled celastrol‐phospholipid NPs efficiently delivered celastrol to damaged endothelial cells and podocytes in the glomerulus, showing potential for treating chronic kidney disease.^[^ ^203^ ^]^ A hyaluronic acid‐coated berberine/tannic acid NPs facilitated targeted colon delivery by exploiting specific interactions between hyaluronic acid and CD44 receptors.^[^ ^73^ ^]^ Maleimide‐modified PLGA NPs loaded with curcumin and astragaloside IV with anti‐PD‐L1 and anti‐PD‐1 antibodies conjugated to their surfaces targeted lung cancer cells to induce apoptosis and activate T cell‐mediated anti‐tumor immunity (Figure 5g).^[^ ^204^ ^]^ Peptide‐modified, liposome‐coated ZIF‐8 NPs loaded with bufalin and sorafenib targeted both tumor and vascular cells, enhancing hepatocellular carcinoma treatment sensitivity, which is often reduced by prolonged sorafenib use (Figure 5h).^[^ ^205^ ^]^ Engineered betulinic acid NPs were designed to enable preferential drug release in acidic ischemic tissues by chemically converting betulinic acid to betulinic amine for stroke treatment (Figure 5i).^[^ ^206^ ^]^ Additionally, targeted drug delivery was facilitated by the surface conjugation of AMD3100, a CXCR4 antagonist. Triptolide was encapsulated in tumor acidity‐sensitive micelles modified with the CRPPR peptide to specifically target the neuropilin‐1 receptor in pancreatic cancer cells (Figure 5j).^[^ ^207^ ^]^

### 5.3 Decrease of Toxicity

The hepatotoxicity and nephrotoxicity of certain CHMs were concerning. Fortunately, nanotechnologies can reduce toxic effects by modifying them. First, nanostructures can be designed for targeted accumulation in diseased areas or specific tissues, thereby minimizing their exposure to healthy tissues. Second, by encapsulating herbal components, nanocarriers allow controlled and slow release, avoiding sharp spikes in blood concentrations that can cause acute toxic reactions.

#### 5.3.1 Toxicity Decreased of Carrier‐Free Nanostructures

**Figure 6:**
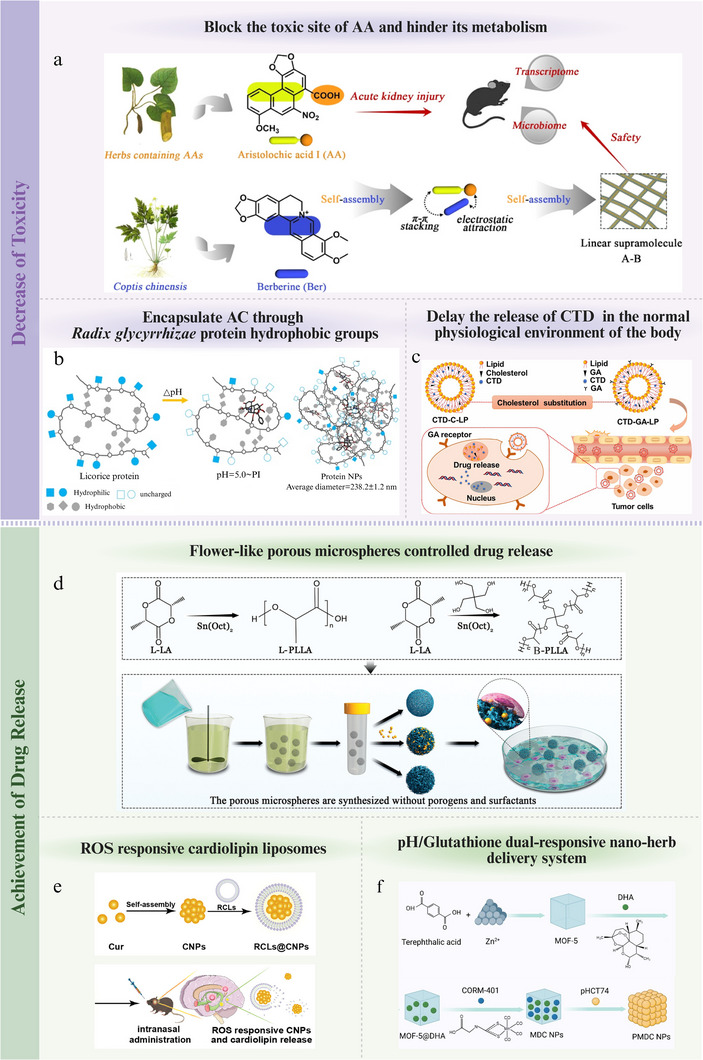
Decrease of toxicity and achievement of drug release via nanotechnology. Decrease of toxicity. a) Ber and aristolochic acid (AA) can self‐assemble into linear structure through π–π stacking and electrostatic attraction. This strategy helps maintain gut microbiota homeostasis and may block the toxic sites of AA, thereby hindering its metabolism. Reproduced with permission.^[^ ^76^ ^]^ Copyright 2021, ACS. b) *Radix glycyrrhizae* reduces the toxicity of aconite primarily through the self‐assembly of *Radix glycyrrhizae* protein (GP) and aconitine (AC) into GP‐AC NPs. In aqueous solution, the polypeptide segment of GP is charged, while the hydrophobic groups are hidden inside the molecule. Near the isoelectric point (pH = 5.0), the protein surface becomes partially uncharged, causing GP to aggregate. During this process, the water‐insoluble AC binds to the hydrophobic regions of GP and is encapsulated in the protein nanoparticles, forming GP‐AC NPs. Reproduced with permission.^[^ ^80^ ^]^ Copyright 2015, Springer. c) Glycyrrhizic acid (GA) is used as a cholesterol substitute to develop GA liposomes loaded with cantharidin (CTD), referred to as CTD–GA–LP. This formulation reduces CTD toxicity through delaying its release in the body's normal physiological environment. Reproduced with permission.^[^ ^216^ ^]^ Copyright 2024, ACS. Achievement of drug release. d) For‐arm poly (L‐lactic acid) (B‐PLLA) is synthesized and then polymerized via LA assisted by Sn (Oct)_2_ to form flower‐like porous microspheres (CFPM), loaded with curcumin. Bone mesenchymal stem cells (BMSC) loaded with CFPM (BMSC@CFPM) deliver a large amount of BMSCs and curcumin, providing controlled drug release. Reproduced with permission.^[^ ^217^ ^]^ Copyright 2023, Wiley. e) CNPs (nanoparticles formed by curcumin self‐assembly) are loaded into cardiolipin liposomes, designed for intranasal administration. These liposomes decompose in response to the oxidative microenvironment in Alzheimer's disease, releasing CNPs and cardiolipin. Reproduced with permission.^[^ ^220^ ^]^ Copyright 2024, Wiley. f) Dihydroartemisinin (DHA) and carbon monoxide‐releasing molecule 401 (CORM‐401) are loaded into metal‐organic framework‐5 (MOF‐5) to develop MDC NPs. The pHCT74 peptide is then modified on the surface of MDC NPs, resulting in PMDC NPs, a dual‐responsive nano‐herb delivery system. Reproduced with permission.^[^ ^222^ ^]^ Copyright 2024, Elsevier.

Self‐assembled NPs can use component combinations to neutralize their potential toxicity. Berberine and aristolochic acid self‐assembled into linear heterogeneous supramolecules through electrostatic attraction and π–π stacking, with hydrophobic groups oriented outward and hydrophilic groups inward, during drug combination (**Figure** **6**a).^[^ ^76^ ^]^ Unlike the use of aristolochic acid alone, this self‐assembly strategy may shield the toxic sites of aristolochic acid and hinder its metabolism, while preserving gut microbiota homeostasis. NPs self‐assembled from celastrol and erianin were designed to reduce in vivo toxicity and enhance therapeutic efficacy.^[^ ^81^ ^]^ Owing to the gastrointestinal irritation and damage caused by celastrol, mice treated with celastrol alone experienced weight loss. However, following the intravenous injection of these NPs, the ERP effect resulted in improved targeting and accumulation at tumor sites, thereby reducing systemic toxicity in mice. *Licorice* reduced the toxicity of *Radix aconiti* while enhancing its efficacy (Figure 6b).^[^ ^80^ ^]^ Purified licorice protein self‐assembled into NPs with aconitine, resulting in mild, recoverable toxicity without mortality, whereas free aconitine caused 100% mortality.

#### 5.3.2 Toxicity Decreased of Carrier‐Based Nanostructures

Nanocarriers facilitate the distribution and cellular uptake of NICHMs in pathological tissues, thereby mitigating the toxic effects of harmful components. Camptothecin is a monoterpene indole alkaloid with anti‐tumor activity;^[^ ^208^ ^]^ however, it is associated with non‐specific toxicity to normal tissues.^[^ ^209^ ^]^ Immunogenic camptothesome vesicles, comprising sphingomyelin‐derived camptothecin bilayers, were designed for cancer immunochemotherapy.^[^ ^210^ ^]^ These vesicles improved the pharmacokinetics and lactone stability of camptothecin, mitigated systemic toxicity, deeply penetrated tumors, and outperformed the anti‐tumor efficacy of onivyde. Triptolide, a diterpenoid triepoxide derived from the herb, *Tripterygium wilfordii*, exhibits potent anticancer^[^ ^211^ ^]^ and anti‐inflammatory^[^ ^212^ ^]^ properties, while its clinical application is limited because of its extreme toxicity.^[^ ^213^ ^]^ To address this issue, triptolide was encapsulated in a tumor pH‐sensitive nanogel.^[^ ^214^ ^]^ Compared to free triptolide, nanogel‐treated mice maintained a stable body weight and exhibited lower liver and renal toxicity, and histopathological analysis further confirmed enhanced biocompatibility. Celastrol, a pentacyclic triterpene, is another toxic compound derived from *T. wilfordii*. One study successfully encapsulated celastrol into PEG‐PCL NPs to form drug‐loaded micelles, which alleviated inflammation and metabolic disorders in obese mice while preventing gastrointestinal irritation and damage commonly associated with celastrol monotherapy.^[^ ^215^ ^]^ Cantharidin, derived from the poisonous CHM *Mylabris*, is effective against hepatocellular carcinoma. A nanosystem was developed in which cantharidin was loaded into glycyrrhizic acid liposomes (with glycyrrhizic acid serving as a cholesterol substitute) (Figure 6c).^[^ ^216^ ^]^ This system selectively exhibited higher toxicity toward HepG_2_ cells and lower toxicity toward LO‐2 hepatocytes, promoting the accumulation of cantharidin in tumor tissues, reducing the accumulation of cantharidin in normal organs, and prolonging the survival of tumor‐bearing mouse models.

### 5.4 Achievement of Drug Release

The formation of nanostructures or encapsulation within nanocarriers slows the degradation and excretion of NICHMs, thereby prolonging drug action. Additionally, the environmental responsiveness of nanocarriers enables precise drug release in specific lesion microenvironments, such as acidic conditions in tumors or high enzyme concentrations in inflamed areas. This controlled‐release mechanism maintains stable concentrations of NICHMs at the target site, reduces dosing frequency, and improves patient compliance.

#### 5.4.1 Non‐Responsive Nanostructures

These CHM‐derived NPs generally lack sensitivity to environmental changes; hence, the release of drugs is typically controlled by the physical degradation of the nanostructure or diffusion of drugs from the particle surface. For instance, flower‐like porous microspheres, designed by combining phase‐inversion emulsification with thermally induced phase separation using four‐arm poly(L‐lactic acid), showed a well‐defined surface topography and inner structure, ensuring a high surface area for incorporating and delivering a large amount of curcumin for sustainable release (Figure 6d).^[^ ^217^ ^]^ Similarly, hyaluronic acid was incorporated into diphenylalanine conjugated with various aromatic moieties through a one‐pot reaction, enabling the dipeptide derivatives to self‐assemble into composite hydrogels with a uniform distribution and excellent mechanical properties; the structure of finer nanofibers and honeycomb networks could facilitate a prolonged release of curcumin.^[^ ^218^ ^]^ Moreover, *Gastrodia elata*, a high‐value dual‐purpose herb and food material containing ≈70% starch by dry weight, was modified with octenyl succinate, a representative hydrophobic reagent, via esterification to produce an amphiphilic hydrocolloid, enabling the starch aggregates to effectively co‐encapsulate and control the release of β‐carotene and curcumin.^[^ ^219^ ^]^

#### 5.4.2 Environmental Responsive Nanostructures

In addition to physical degradation and diffusion, responsive CHM‐derived NPs can control drug release through stimulus‐responsive chemical or physical changes, such as ROS, pH, glutathione, temperature, and other factors. Cardiolipin liposomes loaded with curcumin NPs were developed to promote efficient microglial polarization and decomposition after intranasal administration in response to the oxidative microenvironment of Alzheimer's disease to release curcumin and cardiolipin (Figure 6e).^[^ ^220^ ^]^ Celastrol and glycyrrhetinic acid, two natural anti‐tumor agents, have been used in combination for tumor treatment. Hyaluronic acid was conjugated with celastrol to form an amphiphilic prodrug, and glycyrrhetinic acid self‐assembled into polymer micelles, enabling redox‐responsive co‐delivery for tumor combination therapy.^[^ ^221^ ^]^ NPs assembled from berberine and chlorogenic acid without carriers exhibited pH‐responsiveness (pH = 5.8) and sustained‐release properties, enabling efficient inhibition of multidrug‐resistant *S. aureus*.^[^ ^74^ ^]^ A pH/glutathione dual‐responsive nano‐herb delivery system was developed for targeted dihydroartemisinin delivery with simultaneous abundant CO release, in which the dual‐responsive behavior of metal‐organic framework‐5 facilitated rapid drug release in the acidic tumor microenvironment upon reaching the tumor site (Figure 6f).^[^ ^222^ ^]^ An immunotherapeutic strategy for colon cancer treatment was developed by combining the innate immune activator astragaloside III with the photodynamic therapy (PDT) reagent, chlorin e6, which is effectively releases at tumor sites and promotes immune cell infiltration into the tumor.^[^ ^223^ ^]^ Certain thermosensitive gels can deliver NICHMs through temperature‐responsive mechanisms.^[^ ^165^, ^167^ ^]^

## 6 New Directions of CHMs Integrated with Pharmaceutical Nanotechnologies

Despite the considerable progress achieved by pharmaceutical nanotechnology in addressing the two key barriers for the CHMs mentioned above, several challenges remain to be addressed for their modernization:

- The application of CHMs is guided by the specific theories. However, understanding these theories has long been hindered by barriers arising from cultural differences between the East and West. Therefore, it is essential to provide scientific evidence supporting our findings.
- Although the therapeutic efficacy of TCM is gradually gaining recognition, continuous optimization is required to meet the demands of modern lifestyles and the changing spectrum of human diseases.
- The rich and diverse components of CHMs impose a substantial workload on drug screening for specific diseases, necessitating the development of novel and efficient drug‐screening methodologies.
- CHM formulations are predominantly oral, with limited dosage forms and issues related to formula stability, standardization, and regulatory barriers. These challenges should be addressed while identifying innovative pathways to reveal the potential of CHMs.

### 6.1 Clarification of CHM Theories

The theories of CHMs serve as vital guiding principles for their clinical application. The scientification of these traditional theories represents a crucial part of the modernization of CHMs. Currently, pharmaceutical nanotechnology demonstrates effectiveness in promoting the scientification of CHM theories.

#### 6.1.1 Synergistic Theory

**Figure 7:**
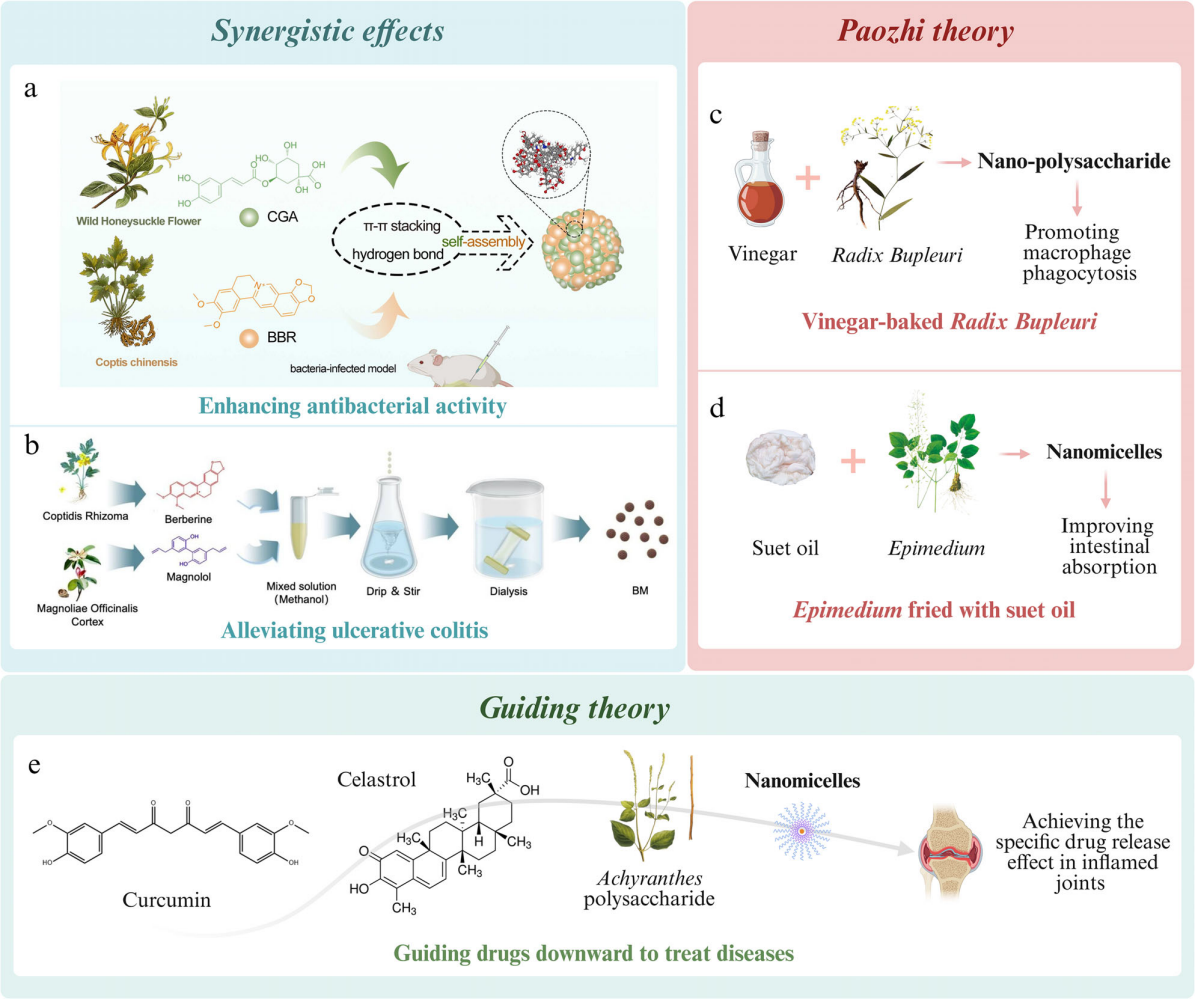
Clarification of TCM Theories. Synergistic effects. a) Berberine (BBR) and chlorogenic acid (CGA) are self‐assembled by π–π stacking and hydrogen bonding, resulting in higher inhibitory effect than single BBR or CGA. Reproduced with permission.^[^ ^74^ ^]^ Copyright 2024, Elsevier. b) BBR and magnolol (MAG) are self‐assembled into nanostructures by π–π stacking and charge interactions, which exhibit superior effects in improving in vivo biodistribution and relieving colitis. Reproduced with permission.^[^ ^69^ ^]^ Copyright 2024, BMC. c,d) *Paozhi* theory. Vinegar‐baked *Radix Bupleuri* forms nano‐polysaccharide that promote macrophage phagocytosis.^[^ ^67^ ^]^ *Epimedium* fried with suet oil enhances the solubility of icariin and improves its intestinal absorption through the formation of self‐assembled nanomicelles.^[^ ^226^ ^]^ e) Guiding theory. A dual‐responsive nano‐delivery system based on *Achyranthes* polysaccharides delivers curcumin and celastrol for the treatment of rheumatoid arthritis.^[^ ^227^ ^]^

CHM formulae consist of multiple components to produce synergistic effects in addressing various diseases.^[^ ^224^ ^]^ Likewise, nanostructures formed by several compounds from different herbs demonstrate superior therapeutic effects compared with those of single ingredients, providing scientific evidence for the synergistic theory of CHMs. For example, SHNs composed of berberine and chlorogenic acid achieved a 99.06% inhibition rate against multidrug‐resistant *S. aureus*, surpassing the effects of berberine or chlorogenic acid alone (**Figure** **7**a).^[^ ^74^ ^]^ In addition, self‐assembled SHNs composed of berberine and magnolol exhibited a synergistic effect in UC mouse models (Figure 7b).^[^ ^69^ ^]^

#### 6.1.2 Paozhi Theory

*Paozhi* enhances the therapeutic effects of raw herbs and reduces their side effects, distinguishing CHMs from other natural remedies.^[^ ^225^ ^]^ Recent studies have reported that through *paozhi*, the components of CHMs can undergo chemical or structural changes, resulting in the formation of nanostructures.^[^ ^59^ ^]^ For instance, the VBCP2.5 micelles have been isolated from vinegar‐baked *Radix Bupleuri* (Figure 7c). Other examples, including *Epimedium* fried with sugar oil, facilitate the self‐assembly of icariin into stable micelles with higher entrapment efficiency, enhanced solubility, and intestinal absorption of icariin (Figure 7d).^[^ ^226^ ^]^

#### 6.1.3 Guiding Theory

Some CHMs possess can directly guide other CHMs to the site of pathogenesis. *Achyranthes*, first recorded in *Shen Nong's Materia Medica*, is known for its ability to guide other herbs to treat diseases. In one study, curcumin‐prodrug micelles were designed using *Achyranthes* polysaccharide as the hydrophilic component and combined with amphiphilic polymers to form mixed micelles for delivering celastrol and curcumin (Figure 7e).^[^ ^227^ ^]^ In these micelles, *Achyranthes* polysaccharide exhibited its “guiding” property, enhancing targeting and increasing the concentrations of celastrol and curcumin at the lesion site, providing evidence from nanostructures to support the role of *Achyranthes* in guiding other medications.

### 6.2 Combination with Topical Treatment of TCM

Topical TCM therapies, such as acupuncture and patches, regulate health through external stimuli. The modification of medical devices with pharmaceutical nanotechnology has the potential to enhance their therapeutic efficacy.

#### 6.2.1 Acupuncture

**Figure 8:**
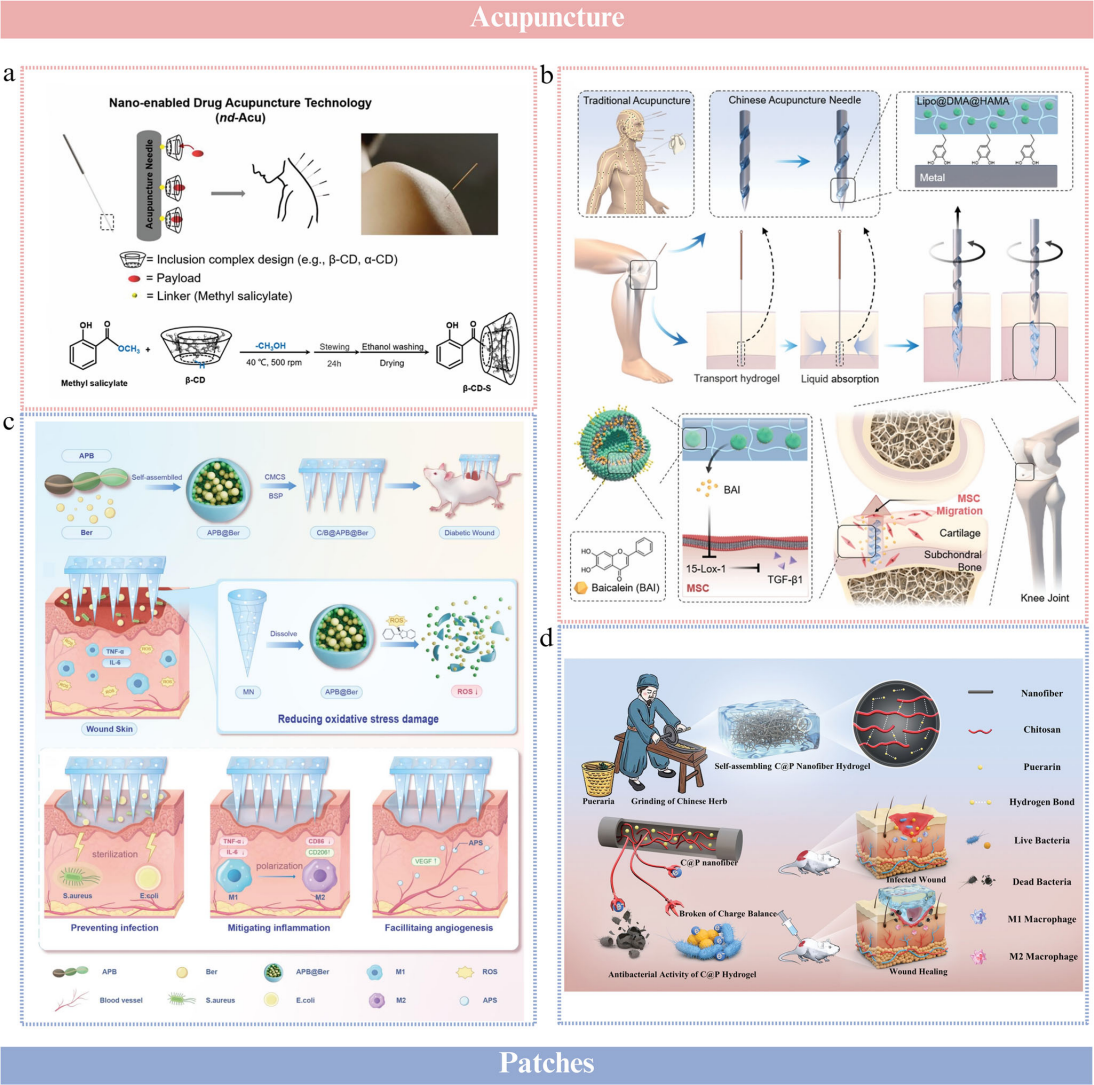
Combination with Topical Treatment of TCM. a) Nano‐enabled drug delivery acupuncture technology (nd‐Acu). In this system, the stainless surface is attached by β‐CD derivative. It facilitates drug encapsulation through inclusion complexation, enabling nano‐enabled drug delivery via acupuncture. Reproduced with permission.^[^ ^229^ ^]^ Copyright 2023, Wiley. b) Chinese acupuncture needles (CA‐needles) with a screw‐thread structure (ST‐needles). The ST‐needles are designed with a screw‐thread structure at the tip to penetrate the subchondral bone. These needles transport a hydrogel loaded with baicalein (BAI), which is continuously released to regulate cytokine secretion at the lesion site. Reproduced with permission.^[^ ^230^ ^]^ Copyright 2022, Wiley. c) Multifunctional traditional Chinese medicine microneedle patch. This microneedle patch is based on the effective components, baicalein (Bai) and berberine (Ber). These components are loaded into ROS‐sensitive nanoparticles of Astragalus polysaccharides (APS), forming a composite nanoparticle formulation (APB@Ber). They are incorporated into multifunctional traditional Chinese medicine composite microneedles (C/B@APB@Ber), which can prevent infection, mitigate inflammation and facilitate angiogenesis. Reproduced with permission.^[^ ^231^ ^]^ Copyright 2024, Elsevier. d) Hydrogel dressings with antibacterial abilities and immune‐regulation properties. Inspired by the grinding treatment of CHMs, mechanical force is applied to enhance molecular collision and accelerate the self‐assembly of chitosan (CS) and puerarin (PUE). This process creates the hydrogel with antibacterial properties, aiding in infection prevention and promoting wound healing. Reproduced with permission.^[^ ^232^ ^]^ Copyright 2022, Wiley.

NPs encapsulating triptolide in human serum albumin ensured the prolonged and controlled release of therapeutic agents when administered via needles.^[^ ^228^ ^]^ Another study confirmed that nanomedicines can enhance the efficacy of acupuncture. A nano‐enabled drug delivery acupuncture needle was developed using an electrochemical procedure to attach methyl salicylate‐modified cyclodextrin, whose sugar rings encapsulated lidocaine, amplifying the treatment of knee osteoarthritis in mouse models (**Figure** **8**a).^[^ ^229^ ^]^ Additionally, an innovative hydrogel‐acupuncture system was developed (Figure 8b).^[^ ^230^ ^]^ The hydrogel with an adhesive polymer interface, prepared via the photo‐crosslinking of *N*‐[2‐(3,4‐dihydroxyphenyl)ethyl]‐2‐methylprop‐2‐enamide and hyaluronic acid methacrylate, was embedded with baicalein carried by liposomes to enable sustained drug release and needle adhesion. Its interface interacted with the metal, was protected by the thread groove of the needle, and remained intact during puncture. At the lesion site, the hydrogel swelled, adhered to surrounding tissues, and remained in place after needle withdrawal, enabling continuous drug release for targeted therapy.

#### 6.2.2 Patches

Several novel microneedle patches incorporating NICHM delivery have been developed, with microneedle tips that disrupt biofilm integrity, facilitate drug diffusion and augment physiological activity compared to traditional patches. The microneedle patch, fabricated with white peony polysaccharide and carboxymethyl chitosan, and loaded with ROS‐sensitive NPs composed of *Astragalus* polysaccharides, baicalein, and berberine, promoted cell proliferation, angiogenesis, and diabetic wound healing by improving the adhesion, biofilm penetration, and dispersion of drugs (Figure 8c).^[^ ^231^ ^]^ Patch dressings have also been optimized, particularly for hydrogel formulations. Inspired by the grinding method of CHMs, mechanical force enhanced the molecular interactions between chitosan and puerarin, promoting their self‐assembly into hydrogel dressings with synergistic antimicrobial and immunomodulatory properties (Figure 8d).^[^ ^232^ ^]^ The design, with varying puerarin ratios, enabled precise control over hydrogel formation, nanofiber structure, and viscoelastic, physicochemical, and biological properties, while its antibacterial activity stemmed from the nanofiber structure and enhanced zeta potential owing to the alignment of amino groups in chitosan. Moreover, synergistic non‐covalent interactions between flavonoid compounds and supramolecular hydrogel agents have been used to form multi‐component dressings, with chain–chain interactions transferring chirality from the composite to the chitosan/polyvinyl alcohol hydrogel, resulting in matched mechanical properties and enhanced therapeutic effects for advanced wound management.^[^ ^233^ ^]^

### 6.3 Utilization for Drug Screening

The identification of the active compounds in CHMs is a vital pathway for contemporary drug development.^[^ ^234^ ^]^ On the one hand, artificial intelligence (AI)‐driven drug screening and molecular dynamics (MD) simulations for self‐assembly prediction hold great potential in drug screening of CHMs. AI technologies can efficiently screen active components from CHMs by employing machine learning and deep learning algorithms^[^ ^235^ ^]^ to analyze vast amounts of chemical composition data of NICHMs, thereby rapidly identifying molecules with potential pharmaceutical value.^[^ ^236^ ^]^ MD simulations are capable of modeling the dynamic changes of molecules under various conditions, assisting researchers in understanding their self‐assembly mechanisms and subsequently optimizing drug delivery systems.^[^ ^237^ ^]^ On the other hand, nanotechnology has considerably broadened the scope of therapeutic approaches. Nano‐modified CHMs offer opportunities for nanodrug development. More importantly, NPs modified with target molecules on their surfaces, including magnetic, metallic, and porous NPs, have recently provided a method for screening bioactive compounds in CHMs.

#### 6.3.1 Magnetic NPs

**Figure 9:**
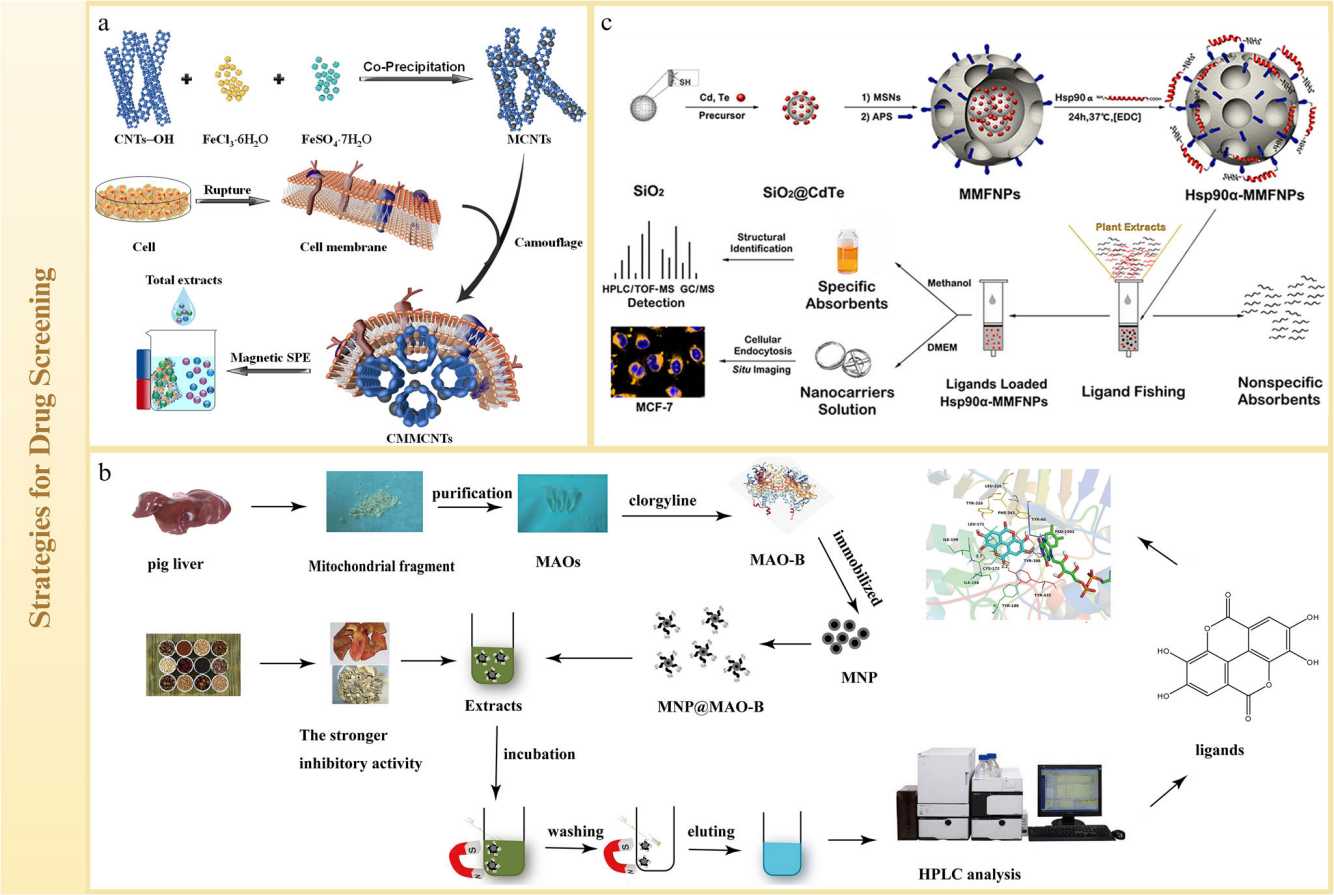
Utilization for Drug Screening. a) Cell membrane camouflaged magnetic carbon nanotubes (CMMCNTs) for screening α1A‐AR antagonists. The positive iron ions are introduced to the surface of CNTs‐OH via electrostatic attraction, followed by in situ generation of Fe₃O₄ upon the addition of ammonia. Then, a cell membrane expressing high levels of α1A‐AR is camouflaged on the MCNTs, resulting in CMMCNTs. These CMMCNTs are then used to screen potential α1A‐AR antagonists from *Radix aconiti*. Reproduced with permission.^[^ ^238^ ^]^ Copyright 2019, Elsevier. b) Magnetic nanoparticle‐based screening of MAO‐B inhibitors. MAO‐B is prepared and immobilized onto magnetic Fe_3_O_4_ nanoparticles (MNP). The MNP‐@MAO‐B is then used for screening MAO‐B inhibitors from *Cistanche fraxini* and *Punica granatum*, coupled with HPLC‐MS and molecular docking analysis. Reproduced with permission.^[^ ^239^ ^]^ Copyright 2019, Elsevier. c) Hsp 90α–MMFNPs for ligand screening. MMFNPs consist of a silica core with fluorescent CdTe QD and a mesoporous silica shell. These nanoparticles are functionalized with Hsp90α in the presence of a cross‐linking agent (Hsp 90α–MMFNPs). Hsp90α–MMFNPs are used to screen for specific ligands by affinity extraction targeting Hsp90 from *Tripterygium wilfordii*. The results are identified through HPLC/TOF‐MS, GC/MS analysis, and morphological imaging. Reproduced with permission.^[^ ^240^ ^]^ Copyright 2018, ACS.

A bifunctional carbon nanotube system was developed by modified with magnetic NPs and overexpressing α1A‐adrenergic receptors in cell membranes (**Figure** **9**a).^[^ ^238^ ^]^ Using this system, mesaconitine and benzoylmesaconitine were identified as potential α1A‐adrenergic receptor antagonists. In addition, magnetic NPs immobilized with monoamine oxidase B, a protein strongly associated with Parkinson's disease, were used to screen for related inhibitors. Calceolarioside B and ellagic acid from *Cistanche fraxini* and *Punica granatum*, respectively, were identified as potential inhibitors (Figure 9b).^[^ ^239^ ^]^

#### 6.3.2 Fluorescent NPs

Quantum dots (QDs) offer the potential to integrate drug screening and in situ imaging capabilities. For example, SiO_2_‐coated QDs, comprising a CdTe QD core and mesoporous SiO_2_ shell functionalized with immobilized heat shock protein 90 (Hsp90), efficiently identified demecolcine and wilforine from *T. wilfordii* as potential Hsp90 inhibitors (Figure 9c).^[^ ^240^ ^]^ Additionally, the formation of G‐quadruplexes in human telomeric DNA inhibits telomerase activity.^[^ ^241^ ^]^ Based on this, a label‐free fluorescence strategy used DNA‐Cu NPs to identify 13 types of NICHMs, including emodin, aloe‐emodin, Rhein, quercetin, luteolin, and kaempferol, as potential telomere‐binding ligands. These advancements demonstrate the potential of pharmaceutical nanotechnology in facilitating drug screening and advancing the discovery of new therapeutics for CHMs.

### 6.4 Diversification of Dosage Forms

CHMs are primarily orally administered. Although the safety of CHM injections has drawn substantial attention, certain well‐established drugs have shown efficacy in treating life‐threatening diseases.^[^ ^242^ ^]^ For example, Xuebijing injection, an herbal‐based intravenous preparation composed of five herbs, significantly reduced mortality in patients with sepsis,^[^ ^243^ ^]^ indicating CHM‐related injections are potentially promising. Moreover, the development of new dosage forms can better enable CHMs to meet modern clinical requirements for therapeutic efficacy, safety, stability, and patient medication adherence, and facilitate the establishment of stricter quality control standards, thereby ensuring the safety and consistency of CHM formulations.

Several nanomedicines derived from CHMs have demonstrated the potential for disease treatment, particularly cancer therapy via intravenous injection.^[^ ^244^ ^]^ Paclitaxel liposome, developed by Luye Pharma and approved in China in 2003, are used as the first‐line treatment for ovarian cancer. Abraxane, developed by Abraxis BioScience, received FDA approval in 2005 for the treatment of various cancers, including bladder cancer. Genexol‐PM for injection, developed by Samyang, were the first approved micelle formulation for metastatic breast cancer, non‐small cell lung cancer, and ovarian cancer and launched in Korea in 2007. Some nanodrugs, such as liposomal vinorelbine tartrate injections, have completed clinical trials for patients with advanced malignancies (NCT02925000). Several formulations are currently undergoing clinical trials, including curcumin and doxorubicin, marketed as Imx‐110, for the treatment of advanced solid tumors (NCT03382340). In addition to injectable formulations, a 1% nano‐curcumin gel is being evaluated for the treatment of oral aphthous ulcers (NCT04385979). Exosomes derived from CHMs have also entered clinical trials. For example, plant‐derived exosomes have been investigated for their potential to deliver curcumin to normal and colon cancer tissues (NCT01294072). Besides, exosomes derived from ginger and aloe plants are being studied for their therapeutic effects in treating and improving polycystic ovary syndrome (NCT03493984).

Notably, in recent years, inhalation formulations have garnered increasing attention. Inhalation therapy delivers drugs directly to the lungs, offering several advantages over systemic drug administration, including rapid onset of action, excellent efficacy, and a favorable safety profile. These characteristics establish inhalation therapy as a clinically irreplaceable modality. Currently, inhalation therapy is applicable for the treatment of most respiratory diseases, including acute asthma attacks, acute exacerbations of chronic obstructive pulmonary disease, acute laryngeal obstruction, acute, subacute, or chronic worsening of cough, and pulmonary infections, among others. Additionally, inhalation therapy has demonstrated clinical utility in treating other systemic diseases; for instance, inhaled loxapine has been used for the management of depression. Thus, inhalation formulations hold substantial potential for broad clinical application. Moreover, inhalation nanomedicines occupy a significant position among nanodrug products in the global pharmaceutical market. A notable example is ARIKAYCE (amikacin liposome inhalation suspension), which was approved by the FDA in 2018. In clinical practice, drugs used for inhalation therapy are predominantly Western pharmaceuticals, while formulations derived from CHMs are rarely employed. Given the excellent safety profile of inhalation therapy, the development of CHM nanoinhalation formulations presents a promising opportunity for the transformation of CHM injections. Therefore, nanoinhalation formulations derived from CHMs are poised for extensive development and application in the future.

## 7 Conclusion

Knowledge on CHMs was accumulated over thousands of years. CHMs have rich pharmacological properties and exert therapeutic effects on a wide range of diseases through multi‐component, multi‐target, and multi‐pathway synergistic actions. Recently, nanomodified CHMs have attracted increasing attention. These nanomaterials constitute a novel and unique class of bioactive substances, characterized by their environmentally friendly and renewable nature, high biocompatibility, and superior therapeutic efficacy, with great potential for the convergence of medicine and engineering.

Nanomaterials derived from CHMs are highly diverse and have been designed to overcome the inherent limitations of the original CHMs. However, there is a lack of a comprehensive summary of this field. Nanocarrier‐free and nanocarrier‐based strategies were presented in this review. We systematically summarized the common structures and characteristics of these two categories. Additionally, we reviewed the advantages and disadvantages of CHMs, and the benefits of nanomodification. Finally, we outlined the remaining challenges in the modernization of CHMs and potential contributions of pharmaceutical nanotechnology, including clarifying CHM theories, enhancing topical therapies, contributing to drug discovery, and promoting the diversification of dosage forms.

However, several issues remain to be resolved. First, the size, surface effects, physical properties, and chemical composition of CHM‐derived nanomaterials result in unique biokinetic processes. Meanwhile, their absorption, distribution, metabolism, and excretion in the human body are not yet fully understood, posing challenges for the determination of drug dosage, design of dosing regimens, and evaluation of safety. Second, research on the therapeutic targets of these nanomaterials is limited. Using advanced technological approaches, such as multi‐omics techniques and molecular imaging, to elucidate targets and mechanisms of action will provide theoretical support for clinical applications and new drug development. Third, the clinical translation of these valuable nanomaterials is important. Unfortunately, it is difficult to simultaneously ensure the purity and yield of synthesized CHMs‐derived nanomaterials, and the purification processes are often complex and costly. Thus, establishing comprehensive quality standards and control systems to strictly regulate and monitor key indicators of these nanomaterials, such as particle size, morphology, purity, stability, and drug loading, is essential to ensure the quality consistency and safety of the products. Overall, nanostructures derived from CHMs represent emerging systems with considerable potential for further development.

## Conflict of Interest

The authors declare no conflict of interest.

## References

[1] a) F. Cheung , Nature 2011, 480, S82; 22190085 10.1038/480S82a

[2] a) H. Gou , H. Su , D. Liu , C. C. Wong , H. Shang , Y. Fang , X. Zeng , H. Chen , Y. Li , Z. Huang , M. Fan , C. Wei , X. Wang , X. Zhang , X. Li , J. Yu , Gastroenterology 2023, 165, 1404; 37704113 10.1053/j.gastro.2023.08.052

[3] Y. Tu , Nat. Med. 2011, 17, 1217. 21989013 10.1038/nm.2471

[4] Y. Liu , J.‐J. Jiang , S.‐Y. Du , L.‐S. Mu , J.‐J. Fan , J.‐C. Hu , Y. Ye , M. Ding , W.‐Y. Zhou , Q.‐H. Yu , Y.‐F. Xia , H.‐Y. Xu , Y.‐J. Shi , S.‐W. Qian , Y. Tang , W. Li , Y.‐J. Dang , X. Dong , X.‐Y. Li , C.‐J. Xu , Q.‐Q. Tang , Science 2024, 384, 5382. 10.1126/science.adk5382 38870290

[5] H. Zhang , P. N. Thai , R. V. Shivnaraine , L. Ren , X. Wu , D. H. Siepe , Y. Liu , C. Tu , H. S. Shin , A. Caudal , S. Mukherjee , J. Leitz , W. T. L. Wen , W. Liu , W. Zhu , N. Chiamvimonvat , J. C. Wu , Cell 2024, 187, 7143. 39413786 10.1016/j.cell.2024.09.034 PMC11645214

[6] B. Jiang , L. Gao , H. Wang , Y. Sun , X. Zhang , H. Ke , S. Liu , P. Ma , Q. Liao , Y. Wang , H. Wang , Y. Liu , R. Du , T. Rogge , W. Li , Y. Shang , K. N. Houk , X. Xiong , D. Xie , S. Huang , X. Lei , J. Yan , Science 2024, 383, 622. 38271490 10.1126/science.adj3484

[7] Z. Xu , Y. Tian , J. Wang , Y. Ma , Q. Li , Y. Zhou , W. Zhang , T. Liu , L. Kong , Y. Wang , Z. Xie , Z. An , B. Zheng , Y. Zhang , C. Cao , C. Liu , L. Tian , C. Fan , J. Liu , H. Yao , J. Song , B. Duan , H. Liu , R. Gao , W. Sun , S. Chen , Sci. Adv. 2024, 10, 3596. 10.1126/sciadv.ads3596 PMC11606445 39612339

[8] X. Liu , E. Lai‐Han Leung , Y. Wang , J. Shi , M. Hu , L. Liu , Z. Liu , Science 2015, 350, S79

[9] A. Lu , Innovation (Camb) 2025, 6, 100811. 40470319 10.1016/j.xinn.2025.100811 PMC12130980

[10] Y. Song , L. Bugada , R. Li , H. Hu , L. Zhang , C. Li , H. Yuan , K. Kumari Rajanayake , N. A. Truchan , F. Wen , W. Gao , D. Sun , Sci. Transl. Med. 2022, 14, l3649. 10.1126/scitranslmed.abl3649 PMC9589917 35507675

[11] C. Feng , P. Tan , G. Nie , M. Zhu , Exploration 2023, 3, 20210263. 37933383 10.1002/EXP.20210263 PMC10624393

[12] Z. Cao , J. Liu , X. Yang , Exploration (Beijing) 2024, 4, 20230037. 39439489 10.1002/EXP.20230037 PMC11491306

[13] A. D. Bangham , R. W. Horne , J. Mol. Biol. 1964, 8, 660. 14187392 10.1016/s0022-2836(64)80115-7

[14] L. J. Kubiatowicz , A. Mohapatra , N. Krishnan , R. H. Fang , L. Zhang , Exploration 2022, 2, 20210217. 36249890 10.1002/EXP.20210217 PMC9539018

[15] E. Callaway , M. Naddaf , Nature 2023, 622, 228. 37783956 10.1038/d41586-023-03046-x

[16] Y. Jia , Y. Jiang , Y. He , W. Zhang , J. Zou , K. T. Magar , H. Boucetta , C. Teng , W. He , Pharmaceutics 2023, 15, 774. 36986635 10.3390/pharmaceutics15030774 PMC10059816

[17] L. Rao , Y. Yuan , X. Shen , G. Yu , X. Chen , Nat. Nanotechnol. 2024, 19, 1769. 39362960 10.1038/s41565-024-01753-8

[18] X. Li , Y. Hu , X. Zhang , X. Shi , W. J. Parak , A. Pich , Nat. Commun. 2024, 15, 8172. 39289401 10.1038/s41467-024-52416-0 PMC11408679

[19] F. Fang , X. Chen , ACS Nano 2024, 18, 23827. 39163559 10.1021/acsnano.4c09027

[20] Y.‐L. Zhang , Y.‐L. Wang , K. Yan , Q.‐Q. Deng , F.‐Z. Li , X.‐J. Liang , Q. Hua , Nanoscale Horiz. 2023, 8, 976. 37278697 10.1039/d3nh00120b

[21] J. Xiang , Y. Meng , M. Zhao , Z. Li , Q. Zhang , N. Wang , Z. Ao , D. Han , Nano Res. 2025, 18, 94907094.

[22] B. Zhao , H. Lin , X. Jiang , W. Li , Y. Gao , M. Li , Y. Yu , N. Chen , J. Gao , Theranostics 2024, 14, 4598. 39239509 10.7150/thno.97096 PMC11373634

[23] a) J.‐F. Tu , G.‐X. Shi , S.‐Y. Yan , G.‐X. Ni , F.‐T. Yu , G.‐W. Cai , Z.‐S. Liu , C.‐Y. Ma , L.‐Q. Wang , J.‐W. Yang , X.‐Q. Zhou , X.‐L. Meng , H.‐Y. Fu , J. Li , W.‐J. Wan , T.‐H. Sun , X.‐Z. Wang , C.‐Z. Liu , JAMA Intern. Med. 2024, 184, 1417; 39401008 10.1001/jamainternmed.2024.5463 PMC11581490

[24] X. Guo , W. Luo , L. Wu , L. Zhang , Y. Chen , T. Li , H. Li , W. Zhang , Y. Liu , J. Zheng , Y. Wang , Adv. Sci. (Weinh) 2024, 11, 2403388. 39033533 10.1002/advs.202403388 PMC11425287

[25] S. Li , X. Chen , H. Shi , M. Yi , B. Xiong , T. Li , Mol. Cancer 2025, 24, 27. 39838407 10.1186/s12943-024-02213-6 PMC11749133

[26] Z. Xi , R. Dai , Y. Ze , X. Jiang , M. Liu , H. Xu , Mol. Cancer 2025, 24, 57. 40001110 10.1186/s12943-025-02245-6 PMC11863959

[27] W. Yu , X. Li , Q. Sun , S. Yi , G. Zhang , L. Chen , Z. Li , J. Li , L. Luo , Food Chem. 2024, 441, 138388. 38219368 10.1016/j.foodchem.2024.138388

[28] Q. Lv , G. Chen , H. He , Z. Yang , L. Zhao , K. Zhang , C. Y. Chen , Signal Transduction Targeted Ther. 2023, 8, 127. 10.1038/s41392-023-01339-1 PMC10063611 36997527

[29] J. Xu , S. Guo , X. Yin , M. Li , H. Su , X. Liao , Q. Li , L. Le , S. Chen , B. Liao , H. Hu , J. Lei , Y. Zhu , X. Qiu , L. Luo , J. Chen , R. Cheng , Z. Chang , H. Zhang , N. C. Wu , Y. Guo , D. Hou , J. Pei , J. Gao , Y. Hua , Z. Huang , S. Chen , Acta Pharm. Sin. B 2023, 13, 2234. 37250171 10.1016/j.apsb.2022.11.015 PMC10213816

[30] X. Zhong , Z. Di , Y. Xu , Q. Liang , K. Feng , Y. Zhang , L. Di , R. Wang , Chin. Med. 2022, 17, 21. 35144660 10.1186/s13020-022-00577-9 PMC8830990

[31] Z. Cui , C. Li , P. Chen , H. Yang , Theranostics 2022, 12, 1829. 35198076 10.7150/thno.68804 PMC8825594

[32] X. Zhai , Q. Wang , M. Li , Lancet 2016, 387, 1722. 10.1016/S0140-6736(16)30261-6 27116281

[33] Y. Jiang , X. Shen , F. Zhi , Z. Wen , Y. Gao , J. Xu , B. Yang , Y. Bai , Cell Death Discovery 2023, 9, 266. 37500645 10.1038/s41420-023-01558-z PMC10374529

[34] a) Y. Dong , K. Jiang , Z. Li , Y. Zhou , B. Ju , L. Min , Q. He , P. Fan , W. Hu , H. Qu , H. Wu , C. Pan , Y. Cao , X. Lou , G. Zhang , J. Zhang , F. Hu , Q. Dong , X. Zhao , R. Xu , L. Guo , X. Zhuang , Y. Zhu , R. Shao , S. Chen , J. She , C. Lu , C. Yan , Q. Wei , W. Hong , et al., JAMA Network Open 2024,7, 2433463;

[35] J. L. Ren , L. Yang , S. Qiu , A. H. Zhang , X. J. Wang , Trends Endocrinol. Metab. 2023, 34, 146. 36710216 10.1016/j.tem.2023.01.005

[36] P. Fan , S. Zhang , Y. Wang , T. Li , H. Zhang , P. Zhang , S. Huang , Nat. Commun. 2024, 15, 1970. 38443335 10.1038/s41467-024-45543-1 PMC10915175

[37] M. Wu , T. Ma , Y. Zhu , H. Ren , L. Fu , Proc. Natl. Acad. Sci. U. S. A. 2024, 121, 2400812121. 10.1073/pnas.2400812121 PMC11572975 39508765

[38] M. Liao , Q. Xie , Y. Zhao , C. Yang , C. Lin , G. Wang , B. Liu , L. Zhu , Pharmacol. Res. 2022, 176, 106077. 35026404 10.1016/j.phrs.2022.106077

[39] P. Wang , T. He , R. Zheng , Y. Sun , R. Qiu , X. Zhang , Y. Xing , H. Shang , J. Ethnopharmacol. 2021, 278, 114214. 34033900 10.1016/j.jep.2021.114214

[40] B. Guo , C. Zhao , C. Zhang , Y. Xiao , G. Yan , L. Liu , H. Pan , Pharmacol. Res. 2022, 175, 106000. 34838694 10.1016/j.phrs.2021.106000

[41] a) Y. Wang , X. Wang , Y. Li , Z. Xue , R. Shao , L. Li , Y. Zhu , H. Zhang , J. Yang , Pharmacol. Res. 2022, 176, 106083; 35033647 10.1016/j.phrs.2022.106083 PMC8757644

[42] J. Wang , W. Liu , Y. Huang , G. Wang , X. Guo , D. Shi , T. Sun , C. Xiao , C. Zhang , B. Jiang , Y. Guo , J. Li , Adv. Sci. (Weinh) 2024, 11, 2401862. 39073681 10.1002/advs.202401862 PMC11423240

[43] a) Y. Zhang , X. Lv , J. Qu , X. Zhang , M. Zhang , H. Gao , Q. Zhang , R. Liu , H. Xu , Q. Li , K. Bi , Acta Pharm. Sin. B 2020, 10, 557; 32140399 10.1016/j.apsb.2019.10.008 PMC7049611

[44] a) J. Wu , S. Deng , X. Yu , Y. Wu , X. Hua , Z. Zhang , Y. Huang , Phytomedicine 2024, 123, 155201; 37976693 10.1016/j.phymed.2023.155201

[45] Q. Lyu , W. Xue , R. Liu , Q. Ma , V. B. Kasaragod , S. Sun , Q. Li , Y. Chen , M. Yuan , Y. Yang , B. Zhang , A. Nie , S. Jia , C. Shen , P. Gao , W. Rong , C. Yu , Y. Bi , C. Zhang , F. Nan , G. Ning , Z. Rao , X. Yang , J. Wang , W. Wang , Nature 2024, 634, 936. 39261733 10.1038/s41586-024-07929-5

[46] J. Zou , Z.‐C. Qiu , Q.‐Q. Yu , J.‐M. Wu , Y.‐H. Wang , K.‐D. Shi , Y.‐F. Li , R.‐R. He , L. Qin , X.‐S. Yao , X.‐L. Wang , H. Gao , ACS Cent. Sci. 2024, 10, 628. 38559293 10.1021/acscentsci.3c01414 PMC10979506

[47] J. Zhang , M. Zhang , W.‐H. Zhang , Q.‐M. Zhu , X.‐K. Huo , C.‐P. Sun , X.‐C. Ma , H.‐T. Xiao , Phytomedicine 2022, 107, 154380. 36150346 10.1016/j.phymed.2022.154380

[48] Y. L. Zhang , Y. L. Wang , K. Yan , H. Li , X. Zhang , J. Milon Essola , C. Ding , K. Chang , G. Qing , F. Zhang , Y. Tan , T. Peng , X. Wang , M. Jiang , X.‐J. Liang , Q. Hua , Adv. Sci. (Weinheim, Ger.) 2024, 11, 2306140. 10.1002/advs.202306140 PMC10837375 38044276

[49] W. Qian , B. Zhang , M. Gao , Y. Wang , J. Shen , D. Liang , C. Wang , W. Wei , X. Pan , Q. Yan , D. Sun , D. Zhu , H. Cheng , J. Pharm. Anal. 2025, 15, 101056. 39974618 10.1016/j.jpha.2024.101056 PMC11835567

[50] a) Q. Liu , Y. Yang , M. Pan , K. Shi , D. Mo , Y. Li , M. Wang , L. Guo , Z. Qian , Bioact. Mater. 2024, 41, 413; 39184827 10.1016/j.bioactmat.2024.07.032 PMC11342206

[51] B. Das , A. T. K. Baidya , A. T. Mathew , A. K. Yadav , R. Kumar , Bioorg. Med. Chem. 2022, 56, 116614. 35033884 10.1016/j.bmc.2022.116614

[52] C. W. Ogle , S. Dai , J. C. Ma , Am. J.Chin. Med. 1976, 04, 147.

[53] a) J. Zhu , Z. Zhang , R. Wang , K. Zhong , K. Zhang , N. Zhang , W. Liu , F. Feng , W. Qu , ACS Appl. Nano Mater. 2022, 5, 3146;

[54] S. C. Lenaghan , L. Xia , M. Zhang , J. Biomed. Nanotechnol. 2009, 5, 472. 20201420 10.1166/jbn.2009.1056

[55] Q. Mao , J. Min , R. Zeng , H. Liu , H. Li , C. Zhang , A. Zheng , J. Lin , X. Liu , M. Wu , Theranostics 2022, 12, 6088. 36168633 10.7150/thno.72509 PMC9475452

[56] W. Luo , Z. Yang , J. Zheng , Z. Cai , X. Li , J. Liu , X. Guo , M. Luo , X. Fan , M. Cheng , T. Tang , J. Liu , Y. Wang , ACS Nano 2024, 18, 28894. 39383335 10.1021/acsnano.4c09097

[57] B. G. Bag , S. S. Dash , Langmuir 2015, 31, 13664. 26671722 10.1021/acs.langmuir.5b03730

[58] a) J. Jiang , Nature 2011, 480, S93; 22190090 10.1038/480S93a

[59] B. Yang , Z. Zhang , J. Song , T. Qi , J. Zeng , L. Feng , X. Jia , 2024, Chin. Med., 19, 14. 38238801 10.1186/s13020-024-00887-0 PMC10797928

[60] Y. Zhuang , J. Yan , W. Zhu , L. Chen , D. Liang , X. Xu , J. Ethnopharmacol. 2008, 117, 378. 18400430 10.1016/j.jep.2008.02.017

[61] Y. Xu , J. Guan , Q. Wang , R. Xue , Z. He , X. Lu , J. Fan , H. Yu , C. Turghun , W. Yu , Z. Li , S. Abay , W. Chen , B. Han , ACS Appl. Mater. Interfaces. 2023, 15, 15946. 36940092 10.1021/acsami.2c21672

[62] J. Fan , H. Yu , X. Lu , R. Xue , J. Guan , Y. Xu , Y. Qi , L. He , W. Yu , S. Abay , Z. Li , S. Huo , L. Li , M. Lv , W. Li , W. Chen , B. Han , ACS Appl. Mater. Interfaces 2023, 15, 8854. 36757908 10.1021/acsami.2c19065

[63] Y. Li , D. Zhang , T. Shi , Y. Yu , Y. Tian , Q. Xie , J. Shi , L. Kong , C. Yang , Z. Zhang , Nano Res. 2023, 16, 5279.

[64] P. Liang , T. Bi , Y. Zhou , Y. Ma , X. Liu , W. Ren , S. Yang , P. Luo , ACS Appl. Mater. Interfaces 2023, 15, 47939. 37791782 10.1021/acsami.3c09494 PMC10591233

[65] a) Y. Xiong , L. Chen , J. Man , Y. Hu , X. Cui , J. Ginseng Res. 2019, 43, 385; 31308810 10.1016/j.jgr.2017.11.004 PMC6606817

[66] X.‐Y. Zhang , J.‐D. Xu , Y. Wang , C.‐Y. Wu , J. Zhou , H. Shen , Y.‐T. Zou , J.‐H. Zhu , S.‐S. Zhou , S.‐L. Li , J. Xu , F. Long , J. Ethnopharmacol. 2023, 311, 116424. 37003400 10.1016/j.jep.2023.116424

[67] X. Wang , Y. Zhao , Y. Wu , L. Liu , M. Liang , M. Han , P. Li , Z. Chen , H. Yan , R. Zhao , Arabian J. Chem. 2022, 15, 104008.

[68] X. Zhang , X. Dong , R. Zhang , D. Hao , J. Zhang , Y. Shen , X. Chai , H. Wang , Y. Wang , Y. Wang , Mater. Des. 2023, 225, 111546.

[69] Y. Xu , Z. Chen , W. Hao , Z. Yang , M. Farag , C. T. Vong , Y. Wang , S. Wang , J. Nanobiotechnol. 2024, 22, 538. 10.1186/s12951-024-02804-x PMC11373475 39227962

[70] J. Zheng , R. Fan , H. Wu , H. Yao , Y. Yan , J. Liu , L. Ran , Z. Sun , L. Yi , L. Dang , P. Gan , P. Zheng , T. Yang , Y. Zhang , T. Tang , Y. Wang , Nat. Commun. 2019, 10, 1604. 30962431 10.1038/s41467-019-09601-3 PMC6453967

[71] Z. Wang , J. Liu , Q. Chen , Y. Wu , Y. Li , M. Ou , S. Tang , Z. Deng , L. Liu , C. Jiang , H. Zhu , Q. Liu , B. Yang , 2025, Adv. Sci. (Weinheim, Ger.), 12, 2412581. 10.1002/advs.202412581 PMC11848569 39783908

[72] T. Li , P. Wang , W. Guo , X. Huang , X. Tian , G. Wu , B. Xu , F. Li , C. Yan , X.‐J. Liang , H. Lei , ACS Nano 2019, 13, 6770. 31135129 10.1021/acsnano.9b01346

[73] S. Chen , Z. Chen , Y. Wang , W. Hao , Q. Yuan , H. Zhou , C. Gao , Y. Wang , X. Wu , S. Wang , J. Adv. Res. 2022, 40, 263. 36100331 10.1016/j.jare.2021.11.017 PMC9481968

[74] S. Fu , X. Yi , Y. Li , Y. Li , X. Qu , P. Miao , Y. Xu , J. Hazard. Mater. 2024, 473, 134680. 38795486 10.1016/j.jhazmat.2024.134680

[75] S. Gao , H. Zheng , S. Xu , J. Kong , F. Gao , Z. Wang , Y. Li , Z. Dai , X. Jiang , X. Ding , H. Lei , Adv. Healthcare Mater. 2023, 12, 2301826. 10.1002/adhm.202301826 37681364

[76] P. Wang , W. Guo , G. Huang , J. Zhen , Y. Li , T. Li , L. Zhao , K. Yuan , X. Tian , X. Huang , Y. Feng , H. Lei , A. Xu , ACS Appl. Mater. Interfaces 2021, 13, 32729. 34247476 10.1021/acsami.1c06968

[77] J. Cui , X. Wang , J. Li , A. Zhu , Y. Du , W. Zeng , Y. Guo , L. Di , R. Wang , ACS Nano 2023, 17, 1464. 10.1021/acsnano.2c10219 36626296

[78] G. You , T. Feng , G. Zhang , M. Chen , F. Liu , L. Sun , M. Wang , X. Ren , Int. J. Pharm. 2021, 601, 120546. 33794322 10.1016/j.ijpharm.2021.120546

[79] W. Pi , L. Wu , J. Lu , X. Lin , X. Huang , Z. Wang , Z. Yuan , H. Qiu , J. Zhang , H. Lei , P. Wang , Bioact. Mater. 2023, 29, 98. 37456579 10.1016/j.bioactmat.2023.06.018 PMC10345197

[80] L.‐j. Ke , G.‐z. Gao , Y. Shen , J.‐w. Zhou , P.‐f. Rao , Nanoscale Res. Lett. 2015, 10, 449. 26586149 10.1186/s11671-015-1155-1 PMC4653129

[81] J. Tian , K. Chen , Q. Zhang , C. Qiu , H. Tong , J. Huang , M. Hao , J. Chen , W. Zhao , Y.‐K. Wong , L. Gao , P. Luo , J. Wang , Q. Du , Chem. Eng. J. 2024, 499, 155709.

[82] Q.‐W. Luo , L. Yao , L. Li , Z. Yang , M.‐M. Zhao , Y.‐Z. Zheng , F.‐F. Zhuo , T.‐T. Liu , X.‐W. Zhang , D. Liu , P.‐F. Tu , K.‐W. Zeng , Small 2023, 19, 2205531. 10.1002/smll.202205531 36549896

[83] R. Huang , W. Sun , W. Li , R. Hu , R. Meng , Z. Peng , R. Yang , T. Huang , J. Du , L. Shang , C. Xie , Chem. Eng. J. 2024, 500, 156869.

[84] Y. Liu , L. Zhang , H. Cai , X. Qu , J. Chang , G. I. Waterhouse , S. Lu , Sci. Bull. 2024, 69, 3127. 10.1016/j.scib.2024.08.011 39183109

[85] J. Sun , L. Zhong , L. Dong , J. Chen , Spectrochim. Acta, Part A 2024, 314, 124244. 10.1016/j.saa.2024.124244 38579425

[86] R. Khamsi , Nature 2020, 582, S19.

[87] Y. Xu , B. Wang , M. Zhang , J. Zhang , Y. Li , P. Jia , H. Zhang , L. Duan , Y. Li , Y. Li , X. Qu , S. Wang , D. Liu , W. Zhou , H. Zhao , H. Zhang , L. Chen , X. An , S. Lu , S. Zhang , Adv. Mater. 2022, 34, 2200905.

[88] P. Liang , T. Bi , Y. Zhou , C. Wang , Y. Ma , H. Xu , H. Shen , W. Ren , S. Yang , Small 2023, 19, 2303498. 10.1002/smll.202303498 37607318

[89] C. Wang , J. Li , K. Liu , J. Li , F. Zhang , X. Ma , Y. Li , C. Zhang , X. Liu , Y. Qu , M. Zhao , W. Li , W. Huang , Y.‐Q. Li , ACS Nano 2025, 19, 2922. 39772431 10.1021/acsnano.4c16766

[90] R. Qiang , H. Huang , J. Chen , X. Shi , Z. Fan , G. Xu , H. Qiu , ACS Appl. Mater. Interfaces 2023, 15, 38653. 37535012 10.1021/acsami.3c06188

[91] R. Nie , J. Zhang , Q. Jia , Y. Li , W. Tao , G. Qin , X. Liu , Y. Tao , Y. Zhang , P. Li , ACS Nano 2024, 18, 22055. 39116283 10.1021/acsnano.4c05266

[92] Z. Deng , Y. Zhang , R. Li , Y. Zhu , C. Xu , B. Gao , W. Wang , C. Ding , B. He , X. Zhu , M. Yang , T. Liang , M. Zhang , Adv. Funct. Mater. 2025, 24, 18683.

[93] W. Luo , L. Zhang , X. Li , J. Zheng , Q. Chen , Z. Yang , M. Cheng , Y. Chen , Y. Wu , W. Zhang , T. Tang , Y. Wang , Nano Res. 2022, 15, 9274.

[94] X. Wang , T. Wu , Y. Yang , L. Zhou , S. Wang , J. Liu , Y. Zhao , M. Zhang , Y. Zhao , H. Qu , H. Kong , Y. Zhang , J. Nanobiotechnol. 2023, 21, 63. 10.1186/s12951-023-01795-5 PMC9946873 36814298

[95] J. Zhou , Z. Sheng , H. Han , M. Zou , C. Li , Mater. Lett. 2012, 66, 222.

[96] S. Zia , M. R. Khan , M. A. Shabbir , R. M. Aadil , Trends Food Sci. Technol. 2021, 114, 275.

[97] J. Li , W. Fu , X. Zhang , Q. Zhang , D. Ma , Y. Wang , W. Qian , D. Zhu , Carbon 2023, 208, 208.

[98] X. Wei , L. Li , J. Liu , L. Yu , H. Li , F. Cheng , X. Yi , J. He , B. Li , ACS Appl. Mater. Interfaces 2019, 11, 9832. 30758177 10.1021/acsami.9b00074

[99] C. Li , P. Han , H. Mao , C. Lv , K. Huang , M. Jin , ACS Appl. Mater. Interfaces 2023, 15, 10441. 36789721 10.1021/acsami.2c21319

[100] T. Tong , H. Hu , J. Zhou , S. Deng , X. Zhang , W. Tang , L. Fang , S. Xiao , J. Liang , Small 2020, 16, 1906206. 32077621 10.1002/smll.201906206 PMC7169479

[101] Y. Jiang , L. Xiao , J. Wang , T. Tian , G. Liu , Y. Zhao , J. Guo , W. Zhang , J. Wang , C. Chen , W. Gao , B. Yang , J. Nanobiotechnol. 2023, 21, 244. 10.1186/s12951-023-02023-w PMC10386222 37507785

[102] J. Xia , J. Wang , F. Liu , Z. Chen , C. Chen , X. Cheng , Y. Chao , Y. Wang , T. Deng , Adv. Healthcare Mater. 2024, 13, 2304674. 10.1002/adhm.202304674 38501303

[103] J. Feng , Q. Xiu , Y. Huang , Z. Troyer , B. Li , L. Zheng , Adv. Mater. 2023, 35, 2207826. 10.1002/adma.202207826 36592157

[104] I. K. Herrmann , M. J. A. Wood , G. Fuhrmann , Nat. Nanotechnol. 2021, 16, 748. 34211166 10.1038/s41565-021-00931-2

[105] a) W. Wang , K. T. K. Nguyen , C. Zhao , H.‐c. Hung , Sci. Adv. 2023, 9, 5517; 10.1126/sciadv.adh5517 PMC10361603 37478176

[106] M. F. Mahomoodally , M. Z. Aumeeruddy , K. R. R. Rengasamy , S. Roshan , S. Hammad , J. Pandohee , X. Hu , G. Zengin , Semin. Cancer Biol. 2021, 69, 140. 31412298 10.1016/j.semcancer.2019.08.009

[107] Y. Teng , Y. Ren , M. Sayed , X. Hu , C. Lei , A. Kumar , E. Hutchins , J. Mu , Z. Deng , C. Luo , K. Sundaram , M. K. Sriwastva , L. Zhang , M. Hsieh , R. Reiman , B. Haribabu , J. Yan , V. R. Jala , D. M. Miller , K. Van Keuren‐Jensen , M. L. Merchant , C. J. McClain , J. W. Park , N. K. Egilmez , H.‐G. Zhang , Cell Host Microbe 2018, 24, 637. 30449315 10.1016/j.chom.2018.10.001 PMC6746408

[108] C. Cui , M. Du , Y. Zhao , J. Tang , M. Liu , G. Min , R. Chen , Q. Zhang , Z. Sun , H. Weng , ACS Appl. Mater. Interfaces 2024, 16, 53460. 39303016 10.1021/acsami.4c10562

[109] X. Wang , R. Tian , C. Liang , Y. Jia , L. Zhao , Q. Xie , F. Huang , H. Yuan , Biomaterials 2025, 313, 122804. 39236631 10.1016/j.biomaterials.2024.122804

[110] K. Sundaram , Y. Teng , J. Mu , Q. Xu , F. Xu , M. K. Sriwastva , L. Zhang , J. W. Park , X. Zhang , J. Yan , S. Q. Zhang , M. L. Merchant , S.‐Y. Chen , C. J McClain , G. W Dryden , H.‐G. Zhang , Small 2024, 20, 2308680. 10.1002/smll.202308680 PMC11102339 38225709

[111] W. Zhan , M. Deng , X. Huang , D. Xie , X. Gao , J. Chen , Z. Shi , J. Lu , H. Lin , P. Li , J. Controlled Release 2023, 364, 644. 10.1016/j.jconrel.2023.11.020 37967723

[112] C. Gao , Y. Zhou , Z. Chen , H. Li , Y. Xiao , W. Hao , Y. Zhu , C. T. Vong , M. A. Farag , Y. Wang , S. Wang , Theranostics 2022, 12, 5596. 35910802 10.7150/thno.73650 PMC9330521

[113] H. A. Kordan , Science 1959, 129, 779.13635013

[114] V. Tinnirello , M. G. Zizzo , A. Conigliaro , M. Tabone , N. R. Ganji , A. Cicio , C. Bressa , M. Larrosa , F. Rappa , G. Vergilio , R. Gasparro , A. Gallo , R. M. Serio , R. Alessandro , S. Raimondo , Biomed. Pharmacother. 2024, 174, 116514. 38574618 10.1016/j.biopha.2024.116514

[115] O. Urzì , M. Cafora , N. R. Ganji , V. Tinnirello , R. Gasparro , S. Raccosta , M. Manno , A. M. Corsale , A. Conigliaro , A. Pistocchi , S. Raimondo , R. Alessandro , Iscience 2023, 26, 107041. 37426343 10.1016/j.isci.2023.107041 PMC10329147

[116] J. Xu , Y. Yu , Y. Zhang , H. Dai , Q. Yang , B. Wang , Q. Ma , Y. Chen , F. Xu , X. Shi , Z. Liu , C. Wang , Nat. Nanotechnol. 2024, 19, 1569. 39054386 10.1038/s41565-024-01722-1

[117] Y. Lv , M. Li , L. Weng , H. Huang , Y. Mao , D. A. Yang , Q. Wei , M. Zhao , Q. Wei , K. Rui , X. Han , W. Fan , X. Cai , P. Cao , M. Cao , J. Exp. Clin. Cancer Res. 2023, 42, 322. 38012650 10.1186/s13046-023-02888-7 PMC10683135

[118] X. Han , Q. Wei , Y. Lv , L. Weng , H. Huang , Q. Wei , M. Li , Y. Mao , D. Hua , X. Cai , M. Cao , P. Cao , Mol. Ther. 2022, 30, 327. 34450250 10.1016/j.ymthe.2021.08.028 PMC8753455

[119] Z. Qiao , K. Zhang , J. Liu , D. Cheng , B. Yu , N. Zhao , F.‐J. Xu , Nat. Commun. 2022, 13, 7164. 36418895 10.1038/s41467-022-34883-5 PMC9684156

[120] S. Tan , Z. Liu , M. Cong , X. Zhong , Y. Mao , M. Fan , F. Jiao , H. Qiao , J. Controlled Release 2024, 368, 355. 10.1016/j.jconrel.2024.02.045 38432468

[121] G. Yan , Q. Xiao , J. Zhao , H. Chen , Y. Xu , M. Tan , L. Peng , J. Controlled Release 2024, 367, 425. 10.1016/j.jconrel.2024.01.060 38295998

[122] X.‐H. Xu , T.‐J. Yuan , H. A. Dad , M.‐Y. Shi , Y.‐Y. Huang , Z.‐H. Jiang , L.‐H. Peng , Nano Lett. 2021, 21, 8151. 34586821 10.1021/acs.nanolett.1c02530

[123] J. Xia , S. Ma , X. Zhu , C. Chen , R. Zhang , Z. Cao , X. Chen , L. Zhang , Y. Zhu , S. Zhang , Sci. Adv. 2022, 8, 1262. 10.1126/sciadv.abj1262 PMC8836824 35148178

[124] S. Han , S. Bi , T. Guo , D. Sun , Y. Zou , L. Wang , L. Song , D. Chu , A. Liao , X. Song , Z. Yu , J. Guo , J. Controlled Release 2022, 348, 250. 10.1016/j.jconrel.2022.05.057 35660631

[125] Z. Weng , Q. Wei , C. Ye , Y. Xu , J. Gao , W. Zhang , L. Liu , Y. Zhang , J. Hu , Q. Zhong , J. Sun , X. Wang , ACS Nano 2024, 18, 5180. 38299982 10.1021/acsnano.3c13164

[126] H. Zhang , R. Wang , C. Wu , W. Feng , Q. Zhong , X. Chen , T. Wang , C. Mao , Biomaterials 2023, 295, 122027. 36805237 10.1016/j.biomaterials.2023.122027

[127] Y. Li , Z. Ye , H. Yang , Q. Xu , Acta Pharm. Sin. B 2022, 12, 2624. 35755280 10.1016/j.apsb.2022.04.013 PMC9214058

[128] K. Y. Wong , Y. Liu , M. S. Wong , J. Liu , Exploration 2024, 4, 20230008. 39175889 10.1002/EXP.20230008 PMC11335462

[129] Z. Zhang , Z. Feng , X. Zhao , D. Jean , Z. Yu , E. R. Chapman , Nat. Commun. 2023, 14, 5256. 37644062 10.1038/s41467-023-41013-2 PMC10465589

[130] Y. Liu , N. Feng , Adv. Colloid Interface Sci. 2015, 221, 60. 25999266 10.1016/j.cis.2015.04.006

[131] X. Wang , W. Zheng , Q. Shen , Y. Wang , Y. Tseng , Z. Luo , X. Wang , L. Shi , C. Li , J. Liu , Signal Transduction Targeted Ther. 2021, 6, 33. 10.1038/s41392-020-00390-6 PMC7840929 33504772

[132] M. Ismail , W. Yang , Y. Li , T. Chai , D. Zhang , Q. Du , P. Muhammad , S. Hanif , M. Zheng , B. Shi , Biomaterials 2022, 287, 121608. 35690021 10.1016/j.biomaterials.2022.121608

[133] S. Yu , D. Li , A. Shi , Y. Long , J. Deng , Y. Ma , X. Li , J. Wen , Y. Hu , X. He , Y. Wu , N. Li , M. Zhao , Biomed. Pharmacother. 2023, 162, 114542. 36989725 10.1016/j.biopha.2023.114542

[134] K. Jiang , K. Tian , Y. Yu , E. Wu , M. Yang , F. Pan , J. Qian , C. Zhan , Nat. Commun. 2024, 15, 6136. 39033145 10.1038/s41467-024-50568-7 PMC11271521

[135] Y. Zhang , Y. Yang , J. Ye , Y. Gao , H. Liao , J. Zhou , Y. Feng , D. Liu , Y. Meng , X. Chen , L. Gao , Y. Liu , Sci. China: Life Sci. 2021, 64, 1097. 33009993 10.1007/s11427-020-1739-6

[136] T. Smith , K. Affram , E. L. Nottingham , B. Han , F. Amissah , S. Krishnan , J. Trevino , E. Agyare , Sci. Rep. 2020, 10, 16989. 33046724 10.1038/s41598-020-73218-6 PMC7552424

[137] a) M. Xue , M.‐X. Yang , W. Zhang , X.‐M. Li , D.‐H. Gao , Z.‐M. Ou , Z.‐P. Li , X.‐J. Li , S.‐H. Liu , S.‐Y. Yang , Int. J. Nanomed. 2013, 8, 4677; 10.2147/IJN.S51262 PMC3862509 24353417

[138] M. Xue , Z.‐Z. Jiang , T. Wu , J. Li , L. Zhang , Y. Zhao , X.‐J. Li , L.‐Y. Zhang , S.‐Y. Yang , Phytomedicine 2012, 19, 998. 22884304 10.1016/j.phymed.2012.06.006

[139] C. Pi , W. Zhao , M. Zeng , J. Yuan , H. Shen , K. Li , Z. Su , Z. Liu , J. Wen , X. Song , R. J. Lee , Y. Wei , L. Zhao , Drug Delivery 2022, 29, 1878. 35748365 10.1080/10717544.2022.2086938 PMC9246235

[140] Y. Zhao , Y.‐X. Chang , X. Hu , C.‐Y. Liu , L.‐H. Quan , Y.‐H. Liao , Int. J. Pharm. 2017, 516, 364. 27884712 10.1016/j.ijpharm.2016.11.046

[141] F. Shi , J.‐H. Zhao , Y. Liu , Z. Wang , Y.‐T. Zhang , N.‐P. Feng , Int. J. Nanomed. 2012, 7, 2033. 10.2147/IJN.S30085 PMC3356207 22619540

[142] B. Zhao , S. Gu , Y. Du , M. Shen , X. Liu , Y. Shen , Int. J. Pharm. 2018, 535, 164. 29107614 10.1016/j.ijpharm.2017.10.040

[143] a) E. Nance , S. H. Pun , R. Saigal , D. L. Sellers , Nat. Rev. Mater. 2022, 7, 314; 38464996 10.1038/s41578-021-00394-w PMC10923597

[144] T. Guo , Y. Zhang , J. Zhao , C. Zhu , N. Feng , J. Nanobiotechnol. 2015, 13, 47. 10.1186/s12951-015-0107-3 PMC4496826 26156035

[145] T. Liu , S. Zhu , Y. Yang , W. Qin , Z. Wang , Z. Zhao , T. Liu , X. Wang , T. Duan , Y. Liu , Y. Liu , Q. Xia , H. Zhang , N. Li , Biomed. Pharmacother. 2024, 171, 116110. 38198955 10.1016/j.biopha.2023.116110

[146] T. Sheth , S. Seshadri , T. Prileszky , M. E. Helgeson , Nat. Rev. Mater. 2020, 5, 214.

[147] B. Li , T. Tan , W. Chu , Y. Zhang , Y. Ye , S. Wang , Y. Qin , J. Tang , X. Cao , Drug Delivery 2022, 29, 75. 34964421 10.1080/10717544.2021.2018523 PMC8735879

[148] L. Sheng , Y. Wei , C. Pi , J. Cheng , Z. Su , Y. Wang , T. Chen , J. Wen , Y. Wei , J. Ma , Int. J. Nanomed. 2023, 18, 7965. 10.2147/IJN.S430769 PMC10757808 38162571

[149] X. Wang , L. Fu , W. Cheng , J. Chen , H. Zhang , H. Zhu , C. Zhang , C. Fu , Y. Hu , J. Zhang , Drug Delivery 2023, 30, 2204207. 37139554 10.1080/10717544.2023.2204207 PMC10332195

[150] J. Xu , H. Hu , X. Qian , D. Zhang , G. Chen , F. Zhang , X. Huang , S. Ma , B. Chen , Q. Zhou , G. Chen , Int. J. Biol. Macromol. 2024, 277, 134404. 39111460 10.1016/j.ijbiomac.2024.134404

[151] Y. Guo , Y. Li , M. Zhang , R. Ma , Y. Wang , X. Weng , J. Zhang , Z. Zhang , X. Chen , W. Yang , Nat. Commun. 2024, 15, 8586. 39362879 10.1038/s41467-024-53010-0 PMC11450208

[152] H. Yuan , C. Qiu , X. Wang , P. Wang , L. Yi , X. Peng , X. Xu , W. Huang , Y. Bai , J. Wei , J. Ma , Y. K. Wong , C. Fu , W. Xiao , C. Chen , Y. Long , Z. Li , J. Wang , Adv. Mater. 2025, 37, 2406662. 10.1002/adma.202406662 39629527

[153] A. C. Wauters , J. F. Scheerstra , M. M. T. van Leent , A. J. P. Teunissen , B. Priem , T. J. Beldman , N. Rother , R. Duivenvoorden , G. Prévot , J. Munitz , Y. C. Toner , J. Deckers , Y. van Elsas , P. Mora‐Raimundo , G. Chen , S. A. Nauta , A. V. D. Verschuur , A. W. Griffioen , D. P. Schrijver , T. Anbergen , Y. Li , H. Wu , A. F. Mason , M. H. M. E. van Stevendaal , E. Kluza , R. A. J. Post , L. A. B. Joosten , M. G. Netea , C. Calcagno , Z. A. Fayad , et al., Nat. Nanotechnol. 2024, 19, 1735. 39085390 10.1038/s41565-024-01727-w PMC11567884

[154] Z. Yu , J. Guo , M. Hu , Y. Gao , L. Huang , ACS Nano 2020, 14, 4816. 32188241 10.1021/acsnano.0c00708

[155] D. Sun , Y. Zou , L. Song , S. Han , H. Yang , D. Chu , Y. Dai , J. Ma , C. M. O'Driscoll , Z. Yu , J. Guo , Acta Pharm. Sin. B 2022, 12, 378. 35127393 10.1016/j.apsb.2021.06.005 PMC8799998

[156] a) M. J. Mitchell , M. M. Billingsley , R. M. Haley , M. E. Wechsler , N. A. Peppas , R. Langer , Nat. Rev. Drug Discovery 2021, 20, 101; 33277608 10.1038/s41573-020-0090-8 PMC7717100

[157] J. Zhang , L. Shen , X. Li , W. Song , Y. Liu , L. Huang , ACS Nano 2019, 13, 12511. 31664821 10.1021/acsnano.9b02875

[158] S. Chen , J. Wu , Q. Tang , C. Xu , Y. Huang , D. Huang , F. Luo , Y. Wu , F. Yan , Z. Weng , S. Wang , Carbohydr. Polym. 2020, 228, 115398. 31635734 10.1016/j.carbpol.2019.115398

[159] Y. Wu , J. Li , X. Zhong , J. Shi , Y. Cheng , C. He , J. Li , L. Zou , C. Fu , M. Chen , Asian J Pharm. Sci. 2022, 17, 206. 35582637 10.1016/j.ajps.2021.12.003 PMC9091603

[160] Z. Li , Y. Zhu , H. Zeng , C. Wang , C. Xu , Q. Wang , H. Wang , S. Li , J. Chen , C. Xiao , X. Yang , Z. Li , Nat. Commun. 2023, 14, 1437. 36918575 10.1038/s41467-023-37150-3 PMC10015032

[161] J. Ding , T. Wang , Z. Lin , Z. Li , J. Yang , F. Li , Y. Rong , X. Chen , C. He , Nat. Commun. 2025, 16, 1222. 39890820 10.1038/s41467-025-56137-w PMC11785995

[162] Q.‐Y. Duan , Y.‐X. Zhu , H.‐R. Jia , S.‐H. Wang , F.‐G. Wu , Prog. Mater. Sci. 2023, 139, 101167.

[163] X. Du , L. Wu , H. Yan , Z. Jiang , S. Li , W. Li , Y. Bai , H. Wang , Z. Cheng , D. Kong , L. Wang , M. Zhu , Nat. Commun. 2021, 12, 4733. 34354068 10.1038/s41467-021-24972-2 PMC8342549

[164] B. Ashrafi , M. Rashidipour , A. Marzban , S. Soroush , M. Azadpour , S. Delfani , P. Ramak , Carbohydr. Polym. 2019, 212, 142. 30832841 10.1016/j.carbpol.2019.02.018

[165] J. A. Luckanagul , C. Pitakchatwong , P. Ratnatilaka Na Bhuket , C. Muangnoi , P. Rojsitthisak , S. Chirachanchai , Q. Wang , P. Rojsitthisak , Carbohydr. Polym. 2018, 181, 1119. 29253940 10.1016/j.carbpol.2017.11.027

[166] H. Madry , L. Gao , A. Rey‐Rico , J. K. Venkatesan , K. Müller‐Brandt , X. Cai , L. Goebel , G. Schmitt , S. Speicher‐Mentges , D. Zurakowski , M. D. Menger , M. W. Laschke , M. Cucchiarini , Adv. Mater. 2020, 32, 1906508. 10.1002/adma.201906508 31763733

[167] D. Xu , L.‐N. Gao , X.‐J. Song , Q.‐W. Dong , Y.‐B. Chen , Y.‐L. Cui , Q. Wang , J. Nanobiotechnol. 2023, 21, 379. 10.1186/s12951-023-02150-4 PMC10583373 37848975

[168] S. Li , T. Zhang , W. Xu , J. Ding , F. Yin , J. Xu , W. Sun , H. Wang , M. Sun , Z. Cai , Theranostics 2018, 8, 1361. 29507626 10.7150/thno.18299 PMC5835942

[169] a) H. Yuan , F. Wang , Z. Wang , D. Gu , W. Huang , C. Fu , X. Wang , J. Ma , Z. Li , L. Dai , X. Zhang , W. Xiao , J. Wang , ACS Mater. Lett. 2023, 5, 2807;

[170] H. Arami , S. Kananian , L. Khalifehzadeh , C. B. Patel , E. Chang , Y. Tanabe , Y. Zeng , S. J. Madsen , M. J. Mandella , A. Natarajan , E. E. Peterson , R. Sinclair , A. S. Y. Poon , S. S. Gambhir , Nat. Nanotechnol. 2022, 17, 1015. 35995855 10.1038/s41565-022-01189-y PMC9649331

[171] M. I. Setyawati , Q. Wang , N. Ni , J. K. Tee , K. Ariga , P. C. Ke , H. K. Ho , Y. Wang , D. T. Leong , Nat. Commun. 2023, 14, 4269. 37460554 10.1038/s41467-023-40015-4 PMC10352264

[172] P. Yuan , L. Liu , A. Aipire , Y. Zhao , S. Cai , L. Wu , X. Yang , A. Aimaier , J. Lu , J. Li , Int. J. Biol. Macromol. 2023, 227, 1015. 36460244 10.1016/j.ijbiomac.2022.11.277

[173] X. Yu , J. Wang , T. Wang , S. Song , H. Su , H. Huang , P. Luo , J. Nanobiotechnol. 2024, 22, 554. 10.1186/s12951-024-02796-8 PMC11389385 39261890

[174] Y. Li , X. Feng , Y. Li , Q. Song , N. Long , X. Fu , Y. Wang , Y. He , H. Yan , C. Li , L. Feng , C. Fan , M. Li , Y. Han , D. Sun , Chem. Eng. J. 2024, 499, 156421.

[175] Z. Zhou , D. Li , X. Fan , S. Lin , Y. Yuan , P. Zhuang , H. Hu , M. Ge , S. Chen , X. Mei , Mater. Des. 2022, 215, 110465.

[176] D. A. J. J. Driessen , P. Zámecnik , T. Dijkema , S. A. H. Pegge , A. C. H. van Engen‐van Grunsven , R. P. Takes , J. H. A. M. Kaanders , T. W. J. Scheenen , Invest. Radiol. 2022, 57, 810. 35776432 10.1097/RLI.0000000000000902 PMC9653098

[177] a) M. Long , Y. Li , H. He , N. Gu , Adv. Healthcare Mater. 2024, 13, 2302773; 10.1002/adhm.202302773 37931150

[178] S. Zanganeh , G. Hutter , R. Spitler , O. Lenkov , M. Mahmoudi , A. Shaw , J. S. Pajarinen , H. Nejadnik , S. Goodman , M. Moseley , L. M. Coussens , H. E. Daldrup‐Link , Nat. Nanotechnol. 2016, 11, 986. 27668795 10.1038/nnano.2016.168 PMC5198777

[179] K. Wang , L. Li , X. Xu , L. Lu , J. Wang , S. Wang , Y. Wang , Z. Jin , J. Z. Zhang , Y. Jiang , ACS Appl. Mater. Interfaces 2019, 11, 10452. 30801182 10.1021/acsami.8b18648

[180] Y. Qian , Y. Cheng , J. Song , Y. Xu , W. E. Yuan , C. Fan , X. Zheng , Small 2020, 16, 2000796. 10.1002/smll.202000796 32633072

[181] A. A Ansari , R. Lv , S. Gai , A. K. Parchur , P. R. Solanki , Archana , Z. A. Ansari , M. Dhayal , P. Yang , M. K. Nazeeruddin , M. M. Tavakoli , Coord. Chem. Rev. 2024, 515, 215942.

[182] H. Cabral , J. Li , K. Miyata , K. Kataoka , Nat. Rev. Bioeng. 2024, 2, 214.

[183] R. Li , T. S. C. Ng , S. J. Wang , M. Prytyskach , C. B. Rodell , H. Mikula , R. H. Kohler , M. A. Garlin , D. A. Lauffenburger , S. Parangi , D. M. Dinulescu , N. Bardeesy , R. Weissleder , M. A. Miller , Nat. Nanotechnol. 2021, 16, 830. 33958764 10.1038/s41565-021-00897-1 PMC8491539

[184] L. Guo , S. Luo , Z. Du , M. Zhou , P. Li , Y. Fu , X. Sun , Y. Huang , Z. Zhang , Nat. Commun. 2017, 8, 878. 29026082 10.1038/s41467-017-00834-8 PMC5638829

[185] Y. Lin , Y. Wan , X. Du , J. Li , J. Wei , T. Li , C. Li , Z. Liu , M. Zhou , Z. Zhong , J. Nanobiotechnol. 2021, 19, 28. 10.1186/s12951-020-00766-4 PMC7819157 33478501

[186] Z.‐J. Ni , C.‐B. Liu , Y. Xue , H. Huang , Y.‐L. Ma , K. Thakur , Y.‐F. Shang , M. R. Khan , Z.‐J. Wei , Food Chem. 2025, 463, 141506. 39368202 10.1016/j.foodchem.2024.141506

[187] Y. Lin , S. Song , H. Guo , Curr. Opin. Food Sci. 2025, 63, 101285.

[188] M. Sienkiewicz , A. Jaśkiewicz , A. Tarasiuk , J. Fichna , Crit. Rev. Food Sci. Nutr. 2022, 62, 6016. 33685299 10.1080/10408398.2021.1895063

[189] R. Luo , M. Lin , C. Fu , J. Zhang , Q. Chen , C. Zhang , J. Shi , X. Pu , L. Dong , H. Xu , N. Ye , J. Sun , D. Lin , B. Deng , A. McDowell , S. Fu , F. Gao , Carbohydr. Polym. 2021, 263, 117998. 33858583 10.1016/j.carbpol.2021.117998

[190] B. Brophy , G. Smolenski , T. Wheeler , D. Wells , P. L'Huillier , G. Laible , Nat. Biotechnol. 2003, 21, 157. 12548290 10.1038/nbt783

[191] a) Y. Yao , Z. Xu , H. Ding , S. Yang , B. Chen , M. Zhou , Y. Zhu , A. Yang , X. Yan , C. Liang , X. Kou , B. Chen , W. Huang , Y. Li , J. Nanobiotechnol. 2025, 23, 108; 10.1186/s12951-025-03146-y PMC11827262 39953594

[192] a) J. Gao , Z. Xia , S. Gunasekar , C. Jiang , J. M. Karp , N. Joshi , Nat. Rev. Mater. 2024, 9, 567; 10.1038/s41583-024-00829-7 38898231

[193] N. L. Hansen , L. Kjaerulff , Q. K. Heck , V. Forman , D. Staerk , B. L. Møller , J. Andersen‐Ranberg , Nat. Commun. 2022, 13, 5011. 36008399 10.1038/s41467-022-32667-5 PMC9411204

[194] K. Kandemir , M. Tomas , D. J. McClements , E. Capanoglu , Trends Food Sci. Technol. 2022, 119, 192.

[195] M. Rahman , S. A. Al‐Ghamdi , K. S Alharbi , S. Beg , K. Sharma , F. Anwar , F. A. Al‐Abbasi , V. Kumar , Drug Delivery 2019, 26, 782. 31357897 10.1080/10717544.2019.1606865 PMC6711158

[196] Y. Li , R. Liu , J. Yang , G. Ma , Z. Zhang , X. Zhang , Biomaterials 2014, 35, 9731. 25189519 10.1016/j.biomaterials.2014.08.022

[197] P. Botella , E. Rivero‐Buceta , J. Controlled Release 2017, 247, 28. 10.1016/j.jconrel.2016.12.023 28027948

[198] X. Du , Y. Lin , Z. Shuai , J. Duan , C. Wang , J. Liu , J. Jiang , J. Wu , M. Zhou , Z. Zhang , Z. Liu , X. Zhou , P. Jing , X. Sun , Z. Zhong , Chem. Eng. J. 2023, 476, 146270.

[199] X. Liang , C. Gao , L. Cui , S. Wang , J. Wang , Z. Dai , Adv. Mater. 2017, 29, 1703135. 10.1002/adma.201703135 28891273

[200] J. Cao , P. Yang , P. Wang , S. Xu , Y. Cheng , K. Qian , M. Xu , D. Sheng , Y. Li , Y. Wei , Q. Zhang , Biomaterials 2021, 269, 120620. 33421709 10.1016/j.biomaterials.2020.120620

[201] J. Zhang , Y. Li , X. Fang , D. Zhou , Y. Wang , M. Chen , Int. J. Pharm. 2014, 476, 185. 25223472 10.1016/j.ijpharm.2014.09.017

[202] Q. Zhao , J. Feng , F. Liu , Q. Liang , M. Xie , J. Dong , Y. Zou , J. Ye , G. Liu , Y. Cao , Z. Guo , H. Qiao , L. Zheng , K. Zhao , Acta Pharm. Sin. B 2024, 14, 2210. 38799625 10.1016/j.apsb.2024.02.005 PMC11119514

[203] Q. Wu , J. Wang , Y. Wang , L. Xiang , Y. Tan , J. Feng , Z. Zhang , L. Zhang , Nano Res. 2022, 15, 3556. 34925707 10.1007/s12274-021-3894-x PMC8666268

[204] Z. Guo , H. Zheng , T. Wang , N. Han , H. Zhang , J. Li , X. Cheng , J. Ye , S. Du , P. Li , Small 2024, 21, 2405752. 10.1002/smll.202405752 39544164

[205] Z. Li , J. Lan , Y. Wu , L. Chen , D. Gu , L. Sun , S. Yang , Y. Shen , T. Zhang , Y. Ding , Chem. Eng. J. 2024, 500, 157231.

[206] S. Zhang , B. Peng , Z. Chen , J. Yu , G. Deng , Y. Bao , C. Ma , F. Du , W. C. Sheu , W. T. Kimberly , Bioact. Mater. 2022, 16, 57. 35386312 10.1016/j.bioactmat.2022.02.033 PMC8958421

[207] J. Feng , M. Xu , J. Wang , S. Zhou , Y. Liu , S. Liu , Y. Huang , Y. Chen , L. Chen , Q. Song , J. Gong , H. Lu , X. Gao , J. Chen , Biomaterials 2020, 241, 119907. 32120315 10.1016/j.biomaterials.2020.119907

[208] M. Kang , R. Fu , P. Zhang , S. Lou , X. Yang , Y. Chen , T. Ma , Y. Zhang , Z. Xi , J. Liu , Nat. Commun. 2021, 12, 3531. 34112794 10.1038/s41467-021-23872-9 PMC8192753

[209] Z. Liu , Y. Yuan , N. Wang , P. Yu , Y. Teng , Eur. J. Med. Chem. 2024, 279, 116872. 39298971 10.1016/j.ejmech.2024.116872

[210] Z. Wang , N. Little , J. Chen , K. T. Lambesis , K. T. Le , W. Han , A. J. Scott , J. Lu , Nat. Nanotechnol. 2021, 16, 1130. 34385682 10.1038/s41565-021-00950-z PMC8855709

[211] N. Mujumdar , T. N. Mackenzie , V. Dudeja , R. Chugh , M. B. Antonoff , D. Borja‐Cacho , V. Sangwan , R. Dawra , S. M. Vickers , A. K. Saluja , Gastroenterology 2010, 139, 598. 20434451 10.1053/j.gastro.2010.04.046 PMC3587769

[212] L. He , Z. Liang , F. Zhao , L. Peng , Z. Chen , Cell Mol. Immunol. 2015, 12, 515. 25308753 10.1038/cmi.2014.92 PMC4496545

[213] Z.‐L. Zhou , Y.‐X. Yang , J. Ding , Y.‐C. Li , Z.‐H. Miao , Nat. Prod. Rep. 2012, 29, 457. 22270059 10.1039/c2np00088a

[214] D. Ling , H. Xia , W. Park , M. J. Hackett , C. Song , K. Na , K. M. Hui , T. Hyeon , ACS Nano 2014, 8, 8027. 25093274 10.1021/nn502074x

[215] J. Zhao , D. Luo , Z. Zhang , N. Fan , Y. Wang , H. Nie , J. Rong , J. Controlled Release 2019, 310, 188. 10.1016/j.jconrel.2019.08.026 31454532

[216] Y. Wang , W. Wang , H. Yao , J. Wang , F. Yang , Y. Zhang , G. Du , G. Han , ACS Appl. Nano Mater. 2024, 7, 1030.

[217] X. Yuan , W. Yang , Y. Fu , Z. Tao , L. Xiao , Q. Zheng , D. Wu , M. Zhang , L. Li , Z. Lu , Y. Wu , J. Gao , Y. Li , Adv. Healthcare Mater. 2023, 12, 2301486. 10.1002/adhm.202301486 37556132

[218] L. Wang , J. Li , Y. Xiong , Y. Wu , F. Yang , Y. Guo , Z. Chen , L. Gao , W. Deng , ACS Appl. Mater. Interfaces 2021, 13, 58329. 34860513 10.1021/acsami.1c16738

[219] Z. Wu , X. Tang , S. Liu , S. Li , X. Zhao , Y. Wang , X. Wang , H. Li , Food Res. Int. 2023, 172, 113136. 37689900 10.1016/j.foodres.2023.113136

[220] Q. Feng , X. Zhang , X. Zhao , J. Liu , Q. Wang , Y. Yao , H. Xiao , Y. Zhu , W. Zhang , L. Wang , Small 2024, 20, 2405781. 10.1002/smll.202405781 39370581

[221] L. Fu , Z. Su , S. Wu , Y. Cheng , C. Hu , J. Zhang , Chin. Chem. Lett. 2024, 36, 110227.

[222] C. Yang , H. Ming , B. Li , S. Liu , L. Chen , T. Zhang , Y. Gao , T. He , C. Huang , Z. Du , J. Controlled Release 2024, 376, 659. 10.1016/j.jconrel.2024.10.043 39442888

[223] X. Wu , H. Yang , X. Chen , J. Gao , Y. Duan , D. Wei , J. Zhang , K. Ge , X.‐J. Liang , Y. Huang , S. Feng , R. Zhang , X. Chen , J. Chang , Biomaterials 2021, 269, 120654. 33434712 10.1016/j.biomaterials.2021.120654

[224] L. Wang , G.‐B. Zhou , P. Liu , J.‐H. Song , Y. Liang , X.‐J. Yan , F. Xu , B.‐S. Wang , J.‐H. Mao , Z.‐X. Shen , S.‐J. Chen , Z. Chen , Proc. Natl. Acad. Sci. U. S. A. 2008, 105, 4826. 18344322 10.1073/pnas.0712365105 PMC2290784

[225] H. Sheridan , B. Kopp , L. Krenn , D. Guo , J. Sendker , Science 2015, 350, S64

[226] L. Cui , E. Sun , Z.‐H. Zhang , X.‐B. Tan , Y.‐J. Wei , X. Jin , X.‐B. Jia , Molecules (Basel, Switzerland) 2012, 17, 12984. 23117437 10.3390/molecules171112984 PMC6268372

[227] C. Guo , N. Diao , D. Zhang , M. Cao , W. Wang , H. Geng , M. Kong , D. Chen , Int. J. Biol. Macromol. 2023, 234, 123677. 36796562 10.1016/j.ijbiomac.2023.123677

[228] S. Ren , H. Liu , X. Wang , J. Bi , S. Lu , C. Zhu , H. Li , W. Kong , R. Chen , Z. Chen , J. Nanobiotechnol. 2021, 19, 409. 10.1186/s12951-021-01157-z PMC8650546 34876139

[229] W. Xu , Y. Xiao , M. Zhao , J. Zhu , Y. Wang , W. Wang , P. Wang , H. Meng , Adv. Sci. 2023, 10, 2302586. 10.1002/advs.202302586 PMC10558644 37555294

[230] F. Lin , Z. Wang , L. Xiang , L. Wu , Y. Liu , X. Xi , L. Deng , W. Cui , Adv. Sci. 2022, 9, 2200079. 10.1002/advs.202200079 PMC9189641 35404511

[231] X. Liu , C. Guo , W. Yang , W. Wang , N. Diao , M. Cao , Y. Cao , X. Wang , X. Wang , H. Pei , Y. Jiang , M. Kong , D. Chen , Carbohydr. Polym. 2024, 345, 122574. 39227108 10.1016/j.carbpol.2024.122574

[232] B. Chen , H. Zhang , J. Qiu , S. Wang , L. Ouyang , Y. Qiao , X. Liu , Small 2022, 18, 2201766. 10.1002/smll.202201766 35491505

[233] M. Sun , S. Peng , C. Zhao , J. Huang , J. Xia , D. Ye , X. Dou , W. Hou , C. Feng , Adv. Funct. Mater. 2022, 32, 2204291.

[234] J. W.‐H. Li , J. C. Vederas , Science 2009, 325, 161. 19589993 10.1126/science.1168243

[235] A. Marchand , S. Buckley , A. Schneuing , M. Pacesa , M. Elia , P. Gainza , E. Elizarova , R. M. Neeser , P.‐W. Lee , L. Reymond , Y. Miao , L. Scheller , S. Georgeon , J. Schmidt , P. Schwaller , S. J. Maerkl , M. Bronstein , B. E. Correia , Nature 2025, 639, 522. 39814890 10.1038/s41586-024-08435-4 PMC11903328

[236] K. Zhang , X. Yang , Y. Wang , Y. Yu , N. Huang , G. Li , X. Li , J. C. Wu , S. Yang , Nat. Med. 2025, 31, 45. 39833407 10.1038/s41591-024-03434-4

[237] N. Zheng , Y. Cai , Z. Zhang , H. Zhou , Y. Deng , S. Du , M. Tu , W. Fang , X. Xia , Nat. Commun. 2025, 16, 604. 39799136 10.1038/s41467-025-55944-5 PMC11724889

[238] Q. Hu , Y. Bu , X. Zhen , K. Xu , R. Ke , X. Xie , S. Wang , Chem. Eng. J. 2019, 364, 269.

[239] X. Jiang , Y. Yuan , L. Chen , Y. Liu , M. Xiao , Y. Hu , Z. Chun , X. Liao , Microchem. J. 2019, 146, 1181.

[240] Y. Hu , Z.‐Y. Miao , X.‐J. Zhang , X.‐T. Yang , Y.‐Y. Tang , S. Yu , C.‐X. Shan , H.‐M. Wen , D. Zhu , Anal. Chem. 2018, 90, 5678. 29644847 10.1021/acs.analchem.7b05295

[241] L. Yang , Y. Wang , B. Li , Y. Jin , Biosens. Bioelectron. 2017, 87, 915. 27664411 10.1016/j.bios.2016.09.055

[242] T. Lan , S. Jiang , J. Zhang , Q. Weng , Y. Yu , H. Li , S. Tian , X. Ding , S. Hu , Y. Yang , W. Wang , L. Wang , D. Luo , X. Xiao , S. Piao , Q. Zhu , X. Rong , J. Guo , Hepatology 2022, 76, 155. 34717002 10.1002/hep.32221 PMC9299589

[243] S. Liu , C. Yao , J. Xie , H. Liu , H. Wang , Z. Lin , B. Qin , D. Wang , W. Lu , X. Ma , Y. Liu , L. Liu , C. Zhang , L. Xu , R. Zheng , F. Zhou , Z. Liu , G. Zhang , L. Zhou , J. Liu , A. Fei , G. Zhang , Y. Zhu , K. Qian , R. Wang , Y. Liang , M. Duan , D. Wu , R. Sun , Y. Wang , et al., JAMA Intern. Med. 2023, 183, 647. 37126332 10.1001/jamainternmed.2023.0780 PMC10152378

[244] X. Shan , X. Gong , J. Li , J. Wen , Y. Li , Z. Zhang , Acta Pharm. Sin. B 2022, 12, 3028. 35865096 10.1016/j.apsb.2022.02.025 PMC9293719

